# Characterization of repetitive DNA landscape in wheat homeologous group 4 chromosomes

**DOI:** 10.1186/s12864-015-1579-0

**Published:** 2015-05-12

**Authors:** Ingrid Garbus, José R Romero, Miroslav Valarik, Hana Vanžurová, Miroslava Karafiátová, Mario Cáccamo, Jaroslav Doležel, Gabriela Tranquilli, Marcelo Helguera, Viviana Echenique

**Affiliations:** CERZOS (CCT – CONICET Bahía Blanca) and Universidad Nacional del Sur, Bahía Blanca, Argentina; Institute of Experimental Botany, Centre of the Region Haná for Biotechnological and Agricultural Research, Šlechtitelů 31, CZ-78371 Olomouc, Czech Republic; The Genome Analysis Centre (TGAC), Norwich Research Park, Norwich, NR4 7UH UK; Instituto Recursos Biológicos, Instituto Nacional de Tecnología Agropecuaria (INTA), Hurlingham, Buenos Aires Argentina; Estación Experimental Agropecuaria Marcos Juárez, Instituto Nacional de Tecnología Agropecuaria (INTA), Marcos Juárez, Córdoba Argentina

## Abstract

**Background:**

The number and complexity of repetitive elements varies between species, being in general most represented in those with larger genomes. Combining the flow-sorted chromosome arms approach to genome analysis with second generation DNA sequencing technologies provides a unique opportunity to study the repetitive portion of each chromosome, enabling comparisons among them. Additionally, different sequencing approaches may produce different depth of insight to repeatome content and structure. In this work we analyze and characterize the repetitive sequences of *Triticum aestivum* cv. Chinese Spring homeologous group 4 chromosome arms, obtained through Roche 454 and Illumina sequencing technologies, hereinafter marked by subscripts 454 and I, respectively.

Repetitive sequences were identified with the RepeatMasker software using the interspersed repeat database mips-REdat_v9.0p. The input sequences consisted of our 4DS_454_ and 4DL_454_ scaffolds and 4AS_I_, 4AL_I_, 4BS_I_, 4BL_I_, 4DS_I_ and 4DL_I_ contigs, downloaded from the International Wheat Genome Sequencing Consortium (IWGSC).

**Results:**

Repetitive sequences content varied from 55% to 63% for all chromosome arm assemblies except for 4DL_I_, in which the repeat content was 38%. Transposable elements, small RNA, satellites, simple repeats and low complexity sequences were analyzed. SSR frequency was found one per 24 to 27 kb for all chromosome assemblies except 4DL_I_, where it was three times higher. Dinucleotides and trinucleotides were the most abundant SSR repeat units. (GA)n/(TC)n was the most abundant SSR except for 4DL_I_ where the most frequently identified SSR was (CCG/CGG)n. Retrotransposons followed by DNA transposons were the most highly represented sequence repeats, mainly composed of CACTA/En-Spm and *Gypsy* superfamilies, respectively. This whole chromosome sequence analysis allowed identification of three new LTR retrotransposon families belonging to the *Copia* superfamily, one belonging to the *Gypsy* superfamily and two TRIM retrotransposon families. Their physical distribution in wheat genome was analyzed by fluorescent *in situ* hybridization (FISH) and one of them, the Carmen retrotransposon, was found specific for centromeric regions of all wheat chromosomes.

**Conclusion:**

The presented work is the first deep report of wheat repetitive sequences analyzed at the chromosome arm level, revealing the first insight into the repeatome of *T. aestivum* chromosomes of homeologous group 4.

**Electronic supplementary material:**

The online version of this article (doi:10.1186/s12864-015-1579-0) contains supplementary material, which is available to authorized users.

## Background

Wheat (*Triticum aestivum* L. em Thell, 2n = 42; AABBDD) has an allohexaploid genome structure that arose from two polyploidization events. The first brought together the genomes of two diploid species related to the wild species *Triticum urartu* (2n = 2× = 14; A^u^A^u^) and a species related to *Aegilops speltoides* (2n = 14; SS) [1]. This hybridization formed the allotetraploid *Triticum turgidum* (2n = 4x = 28; AABB) that suffered the second hybridization event with a diploid grass species, *Aegilops tauschii* (DD), producing the ancestral allohexaploid *T. aestivum* (2n = 6x = 42; AABBDD) [1]. Thus, the hexaploid wheat genome is characterized by its large size (~17 Gb) and complexity, with repetitive sequences accounting for ~ 80% of the genome [2,3].

The number and complexity of repetitive elements varies between species, and those with larger genomes generally have more repetitive elements [4]. Repetitive sequences can be divided into three main classes: transposable elements, tandem repeats, and high copy number genes, such as ribosomal or histone genes. Transposable elements (TEs) are the best-defined class and constitute the most abundant component of many genomes, ranging from 10% to 85% [5]. Based on transposition mechanism, TEs can be subdivided into two classes. Class I, retrotransposons, move via so-called “copy and paste” mechanisms using RNA intermediates, and is mainly composed of long terminal repeat (LTR) retrotransposons and non-LTR retrotransposons, such as LINEs and SINEs (long and short interspersed nuclear elements, respectively) [6]. Class II DNA transposons replicate without an RNA intermediate, either by a cut-and-paste mechanism (terminal inverted repeats; TIRs), by rolling-circle DNA replication (helitrons), or by mechanisms that remain unknown [5,6].

Tandem repeats represent a second class of repetitive sequences that can account for a large portion of genomic DNA, comprising any sequence found in consecutive copies along a DNA strand, arranged in tandem arrays of the monomeric unit [7]. Typically localized to specialized chromosome regions such as centromeres, telomeres, and heterochromatic knobs of many eukaryotes [8], tandem repeats can be categorized according to the size of the repeated units. Microsatellites or simple sequence repeats consist of 1–6 nucleotides, minisatellites are 10–60 nucleotides, and satellites include more than 60 nucleotides. Satellites are the main class of tandem repeats and are thought to play a role in organizing and stabilizing the specialized chromosome regions in which they are found, which are important for chromosome behavior during cell division [9]. Whereas some satellite repeats are chromosome-specific, others are more broadly distributed [9,10].

Repetitive sequences have a large influence on genome structure, function and evolution but, at the same time, complicate genomic analysis. These highly variable genome components, especially TEs, are subject of dynamic evolution mainly due to insertions, illegitimate and unequal recombination, and interchromosomal and tandem duplications [11]. In bread (hexaploid) wheat, polyploidization and the prevalence of TEs has resulted in massive gene duplication and movement. From a practical point of view, repetitive sequences constitute a potential source of a wide range of markers useful for genome diversity and evolution analysis, genetic mapping and marker-assisted selection. Among them we can find markers based on short tandem repeats, such as Sequence Tagged Microsatellite Sites (STMS) [12] and Simple Sequence Repeats (SSRs) (reviewed in [13]), or markers based on transposable elements like: sequence-specific amplification polymorphism (SSAP) [14], retrotransposon based insertion polymorphism (RBIP) [15], interretrotransposon amplified polymorphism (IRAP) and retrotransposon-microsatellite amplified polymorphism (REMAP) [16], repeat junction– junction marker (RJJM) [17], insertion-site-based polymorphism (ISBP) [18,19], and repeat junction marker (RJM) [20].

The complete characterization of TEs, as well as the elucidation of their distribution across genomes and the mechanisms responsible for that distribution, constitutes essential information for understanding the nature and consequences of genome size variations between different species, as well as the large-scale organization and evolution of plant genomes. However, this type of analysis is hindered by the large genome and the polyploid nature of bread wheat. The International Wheat Genome Sequencing Consortium (IWGSC, [21] has adopted the flow-sorted chromosome arms genomic approach to the analysis of the wheat genome, achieving a great reduction in complexity [22]. The combination of second generation sequencing technologies and DNA from flow-sorted chromosomes and chromosome arms became base for survey sequencing of all chromosome arms of wheat [3]. With some limitations in the building of contigs/scaffolds, this survey sequences provides unique opportunity to study the repetitive portion of each chromosome individually, enables comparisons among different chromosomes [23], and may enable identification of chromosome or genome specific sequences.

As members of IWGSC [21], our laboratory obtained a survey sequence of wheat chromosome arms 4DS and 4DL and, through a combination of different approaches, a virtual map including 1973 syntenic genes was built and ~5,700 genes were predicted on bread wheat chromosome 4D [24]. An even distribution of repetitive elements was also reported in both arms [24], but the repeat fraction of this chromosome was not characterized. Here, we focused on chromosome 4D repeatome and analyzed and characterized the repetitive sequences of chromosome 4D arms obtained through Roche 454 sequencing technology (JROL0000000, [24] and compared it with the 4A, 4B and 4D sequences obtained through Illumina sequencing technology [3], hereinafter differenced with the subscripts 454 and I, respectively. Identified transposable elements were analyzed and sorted by class and classified to families. Novel LTR subfamilies were identified, analyzed, and characterized using specific bioinformatics tools. Their physical localization and distribution along the whole wheat genome was assessed by fluorescent *in situ* hybridization (FISH).

## Results and discussion

### Quantification of repetitive sequences from wheat homeologous group 4 chromosome arms

The repetitive elements were assessed through homology-based comparison with the MIPS Repeat Element Database using the assemblies obtained from Roche 454 survey sequences of chromosome arms of wheat chromosome 4D (4DS_454_ and 4DL_454_) and Illumina sequences of all chromosome arms of wheat chromosome group 4. Computational identification, classification and masking of repetitive elements, including low complexity regions using the RM software yielded 67.4% and 65.6% for 4DS_454_ and 4DL_454_ and 55.0% and 38.7% for 4DS_I_ and 4DL_I_ masked bases, respectively (Table 1; Additional file 1: Tables S1, Additional file 2: Table S2 and Additional file 3: Table S3). On the other hand, 4AS_I_, 4AL_I_, 4BS_I_ and 4BL_I_ were composed of 63.8%, 56.6%, 59.8% and 57.0% of repetitive sequences, respectively (Table 1; Additional file 1: Tables S1, Additional file 2: Table S2 and Additional file 3: Table S3). Similarly, repetitive DNA contents of the diploid A and D genome contributors of hexaploid wheat, *T. urartu* and *Ae. tauschii,* were reported to be 67% [25] and 66% [26], respectively.

**Table 1:** **Repetitive elements identified in** ***Triticum aestivum*** **(var. Chinese Spring) homeologous group 4 chromosome arms**

	**4AS**_**I**_			**4BS**_**I**_			**4DS**_**I**_			**4DS**_**454**_		
	**#**	**Length**	**%**	**#**	**Length**	**%**	**#**	**Length**	**%**	**#**	**Length**	**%**
Retroelements	315566	167160901	59.21	268357	164853117	53.49	112608	69191801	48.69	20607	19344019	50.12
DNA transposons	38628	11506157	4.08	49240	15027055	4.88	25899	8060073	5.67	9835	5969965	15.47
Unclassified:	1117	194275	0.07	1303	311827	0.1	707	161819	0.11	1849	539049	1.40
Small RNA:	265	47072	0.02	319	51039	0.02	184	37472	0.03	69	22419	0.06
Satellites:	819	144631	0.05	3929	2914989	0.95	569	116493	0.08	21	2468	0.01
Simple repeats:	10379	583006	0.21	12326	709023	0.23	5394	329739	0.23	1106	47817	0.12
Low complexity:	9628	497086	0.18	9794	510460	0.17	5487	286086	0.2	1293	83621	0.22
**Total**	**376402**	**180133128**	**63.82**	**345268**	**184377510**	**59.84**	**150848**	**78183483**	**55.01**	**34780**	**26009358**	**67.4**
	**4AL**_**I**_			**4BL**_**I**_			**4DL**_**I**_			**4DL**_**454**_		
	**#**	**Length**	**%**	**#**	**Length**	**%**	**#**	**Length**	**%**	**#**	**Length**	**%**
Retroelements	343182	180465664	49.86	269612	124807259	50.19	328691	116699122	33.57	15107	14212381	52.61
DNA transposons	71092	21753200	6.01	51423	14335731	5.77	53046	12099355	3.48	6296	3030966	11.22
Unclassified:	2334	561199	0.16	1405	345580	0.14	1622	328062	0.09	1258	342679	1.27
Small RNA:	535	101456	0.03	273	42903	0.02	349	46900	0.01	55	14356	0.05
Satellites:	1695	299708	0.08	3051	1041847	0.42	2128	296676	0.09	16	2622	0.01
Simple repeats:	13225	727067	0.2	10237	571582	0.23	47444	3205296	0.92	750	31119	0.12
Low complexity:	14467	772219	0.21	8455	453240	0.18	35801	1723366	0.5	1104	74281	0.27
**Total**	**446530**	**204680513**	**56.55**	**344456**	**141598142**	**56.95**	**469081**	**134398777**	**38.66**	**24586**	**17708404**	**65.55**

The chromosome arms are expressed by a number that indicates the homeologous group followed by a letter that indicates the genome (A, B or D) and the chromosome arm (S: short; L: long). The subscripts refer to the technology used for sequencing (I: Illumina; 454: Roche 454). For each element class the number of elements (#), the length of the sequence occupied by these elements (length) and the percentage of the sequence that is covered by repetitive elements (%) are given.

Comparisons among the percentage of the repeat fraction of chromosome arms reported using Roche 454 sequencing technology [19,27-31] and the ones obtained through Illumina [21], suggest that the first ones constitute a better representation on the real status (Table 2). This assumption is further supported by previous estimations of 75-90% repetitive sequence content of bread wheat genome [19,20]. It has been reported that the longer Roche 454 reads as compared to the Illumina reads, results in an improvement in mapping of repetitive regions [32]. In addition to the sequencing platform, it is necessary to take into account that the estimation of repetitive DNA content depends on the method used. Using mathematically defined repeats (MDRs) [33] it was estimated that the assembly of all chromosome survey sequences of wheat contains 76.6% of MDRs - 20mers with abundance over 1000 copies [3]. The difference observed could be caused by the limited representation in the databases used for masking.

**Table 2:** **Comparison of the repeat content of** ***T. aestivum*** **chromosomes and chromosome arms obtained through different sequencing technologies**

**Chromosome**	**454**		**Illumina**	
	**Repeat content**	**Coverage**	**Repeat content**	**Coverage**
3AS	79% [28]	3.2%	62%	56%
3B	86% [19]		-	64%
4AS	80% [29]	-	63%	89%
4AL	73% [29]	-	56%	66%
4BS	-		60%	97%
4BL	-		57%	58%
4DS	67%	44%	55%	62%
4DL	66%	29%	39%	83%
5AS	76% [27]	21%	67%	67%
5AL	82% [27]		64%	60%
5BS	72% [31]	6%	60%	60%
5BL	71% [31]	8%	55%	72%
6BS	77% [30]	55.6%	58%	51%
6BL	86% [30]	54.9%	59%	52%

The compared chromosomes and chromosome arms are listed on the first column. The following columns show the repeat content, expressed as the percentage of the total sequenced bases and the chromosome coverage calculated as the ratio between the available chromosome or chromosome arm length and the predicted one [74] using as input 454 or Illumina sequences. When data was obtained from literature, the references are cited.

The repetitive regions of 4DS_454_ and 4DL_454_ were almost homogeneously distributed along both chromosome arms [24] what, likely, may be due to limitations of repetitive sequences assembly, used genetic map and GenomeZipper which are positively biased toward the gene-containing regions [34].

### Classification of repetitive sequences from wheat homeologous group 4 chromosome arms

**Figure 1:**
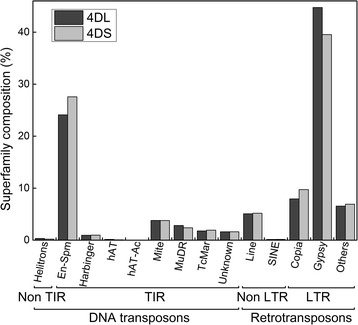
Distribution of DNA and RNA transposon superfamilies on 4D chromosome arms. Bars represent the percentage of each superfamily with respect to the total DNA or RNA transposable elements, for 4DL (dark grey bars) and 4DS (light grey bars). Classification was performed as suggested in [6].

The RM software was further used to classify recognizable repeat families. RNA retrotransposons were the most highly represented sequence repeats, accounting for ~50% of all chromosome arms except for 4DL_I_, in which it was estimated to be 33% of the total sequenced length (Table 1). However, retroelements represented between 74% to 92% of the total repeat fraction in the eight datasets of the homeologous group 4 chromosome arms. The retroelements were followed in abundance by DNA elements, which comprised between 7% to 23% of total repeats. Thus, the DNA and RNA transposons represented more than 96% of the repeat fraction for all the chromosome arms. DNA transposons and retrotransposons were further subclassified, according to the Wicker’s criteria [6], revealing that for 4D chromosome arms, CACTA/En-Spm DNA-transposons and *Gypsy* retrotransposons were the most abundant superfamilies (Figure 1).

**Figure 2:**
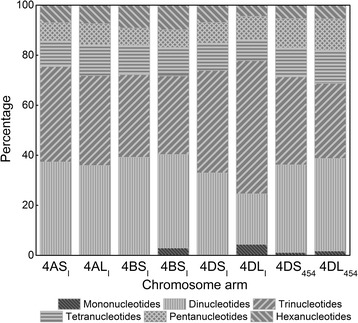
SSR classification according to the size of repeat units. The graph illustrates the frequency of the different sizes of the SSRs repeat units classified as dinucleotides, trinucleotides, tetranucleotides, pentanucleotides and hexanucleotides across the homeologous group 4 chromosome arms from *T. aestivum* obtained through Illumina (4AS_I_, 4AL_I_, 4BS_I_, 4BL_I_, 4DS_I_ and 4DL_I_) and 454 (4DS_454_ and 4DL_454_) sequencing technologies.

The analysis and characterization of satellites, simple repeats and low complexity regions were performed along the wheat homeologous group 4 chromosome arms sequences included in this study (Table 1; Additional file 1: Table S1, Additional file 2: Table S2 and Additional file 3: Table S3). Small RNA, satellites, simple repeats and low complexity sequences represented only small proportions of assemblies of all chromosome arms (Table 1). This finding is not surprising, because these loci derive from repetitive AT and GC-rich sequences that may be collapsed or represented by uneven read coverage in Illumina sequences [35]. This assumption is corroborated by the finding that the GAA microsatellite is not observed within the most abundant microsatellites detected (Additional file 2: Table S2), although its presence has been previously evidenced by using FISH, where the GAA designed probe creates large blocks of signal on all chromosomes [36]. On the other hand several of the trinucleotide SSRs identified as the most abundant in the survey sequences provide on FISH weak disperse signals (Kubaláková, personal communication). The presence of SSRs was analyzed in order to search for new putative markers for physical and genetic mapping. The frequency of SSRs ranged from one SSR per 24 to 27 kb for 4AS_I_, 4AL_I_, 4BS_I_, 4BL_I_, 4DS_I_, 4DS_454_ and 4DL_454_ chromosome arms. For 4DL_I_ assemblies, the frequency was notably higher reaching one SSR per 7.2 kb. Variable SSR frequency has been reported among other chromosomes or chromosome arms. For example, the frequency reported for 5BS and 5BL was one SSR per 19 and 23 kb, respectively, whereas 3B and 3AS chromosomes have a SSR frequency of one SSR per 6.1 and 10.4 kb, respectively [19,28]. The SSR frequencies are higher in transcribed regions than in non-coding regions, being the SSR frequency inversely related to the proportion of repetitive DNA [37]. Thus, the lower repeat content of 4DL_I_ respect either to 4DL_454_ or to the other chromosome arms from the homeologous group 4 agrees with the higher frequency of SSR. This is also in agreement with the gene content calculated for chromosome 4D [24]. The analysis of SSR motifs according to the size of repeat units revealed that dinucleotides and trinucleotides were the most abundant SSRs (Figure 2). In comparison, the trinucleotide constitutes the most frequent SSR motif in *Brachypodium*, rice and maize whereas papaya shows a higher frequency of dinucleotide motifs and soybean a higher frequency of tetranucleotide motifs [38]. Microsatellites were further divided into three classes, AT-rich (greater than 50% A or T in the motif), AT/GC-balanced (limited to di- and tetra-nucleotide motifs that fit this criterion) and GC-rich motifs. The analysis revealed the predominance of AT/GC balanced motifs on the chromosome arms 4AS_I_, 4AL_I_, 4BS_I_, 4BL_I_ and 4DS_454_ whereas the most abundant on 4DL_454_, 4DS_I_ and 4DL_I_ were the GC-rich motifs (Table 3). The combination of both analysis, i.e., the size and nucleotide composition of the repeat unit, revealed that more than the 80% of the dinucleotides belongs to the AT/GC-balanced class, mainly (GA)n/(TC)n, which is also the most abundant dinucleotide motif in *Brachypodium* and *Arabidopsis* while in papaya and soybean the most abundant dinucleotide is AT/TA [38]. Except for 4DL_454_, the GC-rich are the prevalent trinucleotide motif in all chromosome arms as it was described for *Brachypodium* and rice whereas in maize, wheat, papaya, *Arabidopsis* and soybean AT-rich trinucleotide repeats were the majority [38]. Interestingly, trinucleotides motifs represent ~50% of the 4DL_I_ SSRs mainly composed of CCG/CGG motifs (~75%). Finally, the AT-rich tetranucleotide SSRs prevail in the majority of the group 4 homeologous chromosome arm survey sequences. These vast data were used to identify SSR motifs specific for the tested chromosome arms or whole chromosomes and we tested this information by their physical localization on metaphase chromosomes. Identification of SSRs with chromosome distinct pattern may have practical implications.

**Table 3:** **SSR classification according to the classes**

	**4AS**_**I**_	**4BS**_**I**_	**4DS**_**I**_	**4DS**_**454**_	**4AL**_**I**_	**4BL**_**I**_	**4DL**_**I**_	**4DL**_**454**_
**AT-rich**	32,4	25,4	25,7	36,4	28,7	23,9	10,2	23,0
**GC-rich**	29,0	28,5	37,4	30,1	29,3	29,8	66,8	40,4
**AT/GC balanced**	38,5	46,1	36,8	33,5	41,9	46,3	23,0	36,6

Microsatellites were classified into three classes, AT-rich: greater than 50% A or T in the motif; GC-rich: greater than 50% G or C in the motif; AT/GC-balanced: equal amount of GC and AT, along the six chromosome arms obtained through Illumina and the two obtained through 454 sequencing technologies.

The SSR markers still have potential for whole genome or sub-genome mapping [39,40] and breeding [13]. Additionally, some of the SSRs were found very useful as physical markers for cytogenetic mapping, metaphase chromosome identification [41] and enhancing chromosome sorting by FISHIS [42]. Since most of the di and tri-nucleotide SSRs were already localized [43] we focused on SSRs with longer subunit. The comparison of sequence occurrence of unique SSR motifs among chromosomes and chromosome arms allowed identification of SSRs suggestive to be putative arm-specific (Additional file 2: Table S2). The (CAGCG)n/(CGCTG)n and (CCGTA)n/(TACGG)n motifs showed specificity for 4DL and (CGTAG)n/(CTACG)n showed specificity for 4BL. Additionally, (TTACG)n/(CGTAA)n was found specific for chromosome 4D. FISH localization on metaphase chromosomes showed that microsatellites produced weak dispersed signals on almost all chromosomes (data not shown). These findings suggest that quantitative assessment of SSRs in the survey sequence assembly may not be representative due to, already above discussed, the possibility of collapsing of highly repetitive tandem repeats in assemblies of short sequencing reads, but catalog of available microsatellites and other repeats can provide useful information for marker candidate sequence identification and marker development.

### Identification and annotation of novel LTR retrotransposons

LTR retrotransposons account for a significant fraction of many genomes and even are the predominant component of some large genomes [6]. Typical structural characteristics include: 1) two highly similar LTR sequences; 2) target site duplications; 3) a primer binding site and a polypurine tract; 4) protein-coding domains for enzymes important to retrotransposition [6]. Additionally, non-autonomous LTR retrotransposons have been described in plants as large retrotransposon derivatives (LARDs) and terminal repeat retrotransposons in miniature (TRIMs), both of which have the typical features of LTR retrotransposons but lack protein-coding capability in their internal domain [44,45].

**Figure 3:**
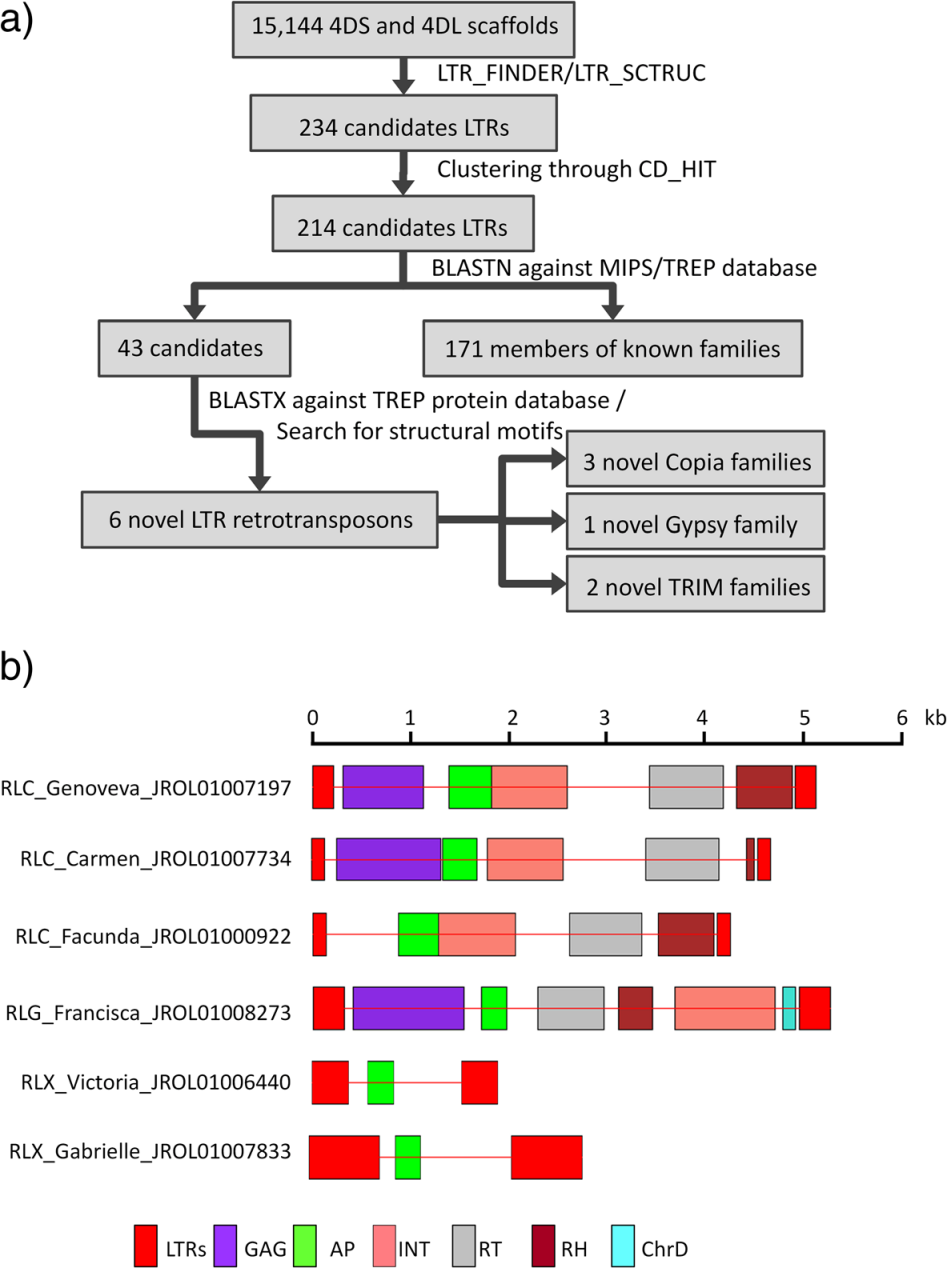
Annotation of novel LTR retrotransposons. **a)** The scheme depicts the steps followed for the identification of novel TEs, starting from the assembled 4DS and 4DL scaffolds, according to the criteria proposed by Wicker et al. [6]. **b)** Graphic representation of the structural features identified in each LTR retrotransposon drawn to scale. GAG: capsid proteins; AP: aspartic proteinase; INT: integrase; RT: reverse transcriptase; RH: RNase H. ChrD: Chromodomain.

The whole 4DS and 4DL scaffolds were further scanned for LTR retrotransposons using the bioinformatics tools LTR_FINDER [46] and LTR_STRUC [47]. The mentioned subset of data was chosen for novel LTR identification due to the larger size when compared to the Illumina contigs, as revealed by size frequency histograms (Additional file 4: Figure S1). The LTR_FINDER and LTR_STRUC outputs lead to 234 candidate sequences (Figure 3a), that were clustered using the CD-HIT interface [48], resulting in 214 unique LTR retrotransposon candidates. After manually search for previously defined elements against MIPS database following the criteria of [6], 171 putative retrotransposons were excluded (Figure 3a). The remaining 43 candidate elements were analyzed for the presence of LTR retrotransposon features using BLASTX searches at NCBI and GyDB [49], reducing the number of candidates for newly identified retrotransposons to six (Table 4). The BLASTX analysis also revealed that likely complete transposon-related proteins were present in four out of the six candidates (JROL01007197, JROL01007734, JROL01000922 and JROL01008273), as judged by the coverage of the alignments with reported proteins, whereas the other two, JROL01006440 and JROL01007833, showed small protein fragments and thus non-coding capacity. The fact that two out of four retrotransposon protein coding regions lack stop codons whereas the other two showed only one indicate that such candidates could encode functional protein sequences. Notice that the presence of few stop codons may not directly imply the absence of functionality of a TE family since only recently inserted elements have not been subject to mutations and could be taken as functional. The identity and coverage of the alignments demonstrate that the novel LTR retrotransposons are members of known superfamilies but constitute novel LTR retrotransposon families (Table 5). Their classification was carried out following the current proposed system [6], revealing that three of the newly identified LTR retrotransposons belonged to the *Copia* superfamily, one was *Gypsy* and the other two were non autonomous terminal repeat retrotransposons in miniature (TRIMs) and thus designations were assigned to the six new families (Tables 5 and 6, Figure 3b). The insertion time of the six newly identified LTR retrotransposons was estimated based on the assumption that the sequences of the two LTRs were identical at the time of integration and accumulated point mutations independently with time. Thus, the nucleotide substitution rate between the two LTRs, considered to reflect the time elapsed since the insertion event, was estimated to be in the range of 0.27 10^6^ to 6.11 10^6^ years (Table 4).

**Table 4:** **Description of the 6 LTR retrotransposon candidates identified on 4D chromosome scaffolds**

**LTR retrotranspoon**	**program**	**LTR retrotransposon** **size**	**# in genome**	**LTR region similarity**	**5′-LTR size**	**3′-LTR size**	**Insertion time (years x 10** ^**6**^ **)**	**TSD**	**PBS**	**PPT**
RLC_Genoveva_JROL01007197	STRUC/FINDER	5132	30	0.978	215	215	0.70	GAGGC	Lys_TT	GCCTCCCTCTTCCTC
RLC_Carmen_JROL01007734	FINDER	4597	40	0.977	131	131	0.27	-	SerTGA	CCATCTTCTTCCTCC
RLC_Facunda_JROL01000922	FINDER	4842	757	0.942	138	139	4.65	-	MetCAT	GATACTGCGGGGGGA
RLG_Francisca_JROL01008273	STRUC/FINDER	5279	21	0.969	321	321	0.70	CTGTC	SerGCT	TCTCCTGGTCCTCCC
RLX_Victoria_JROL01006440	FINDER	1898	121	0.960	375	375	0.97	-	MetCAT	TCATCCTCTCGCCCT
RLX_Gabrielle_JROL01007833	STRUC	2644	33	0.935	685	680	6.11	ACATT	MetCAT	ATAGCTTCGTTCCAAGAAGGAGGGGA

The designations on the new LTR retrotransposons are indicated in column 1. The number of genomic repetitions for each candidate LTR retrotransposon was estimated by searching against the *T. aestivum* chromosome arm contigs deposited in the URGI database (# in genome). LTR: Long terminal repeat; TSD: target site duplication; PBS: primer binding site; PPT: polypurine tract. The last column indicates the presence (+) or absence (-) of retrotransposon proteins when BLAST searched against the TREP protein database.

**Table 5:** **BLASTX alignment of coding sequences encoded by the novel LTR retrotransposons with TREP database**

**LTR retrotransposon**	**TREP protein code** ^**1**^	**LTR retrotransposon associated** ^**2**^	**Score** ^**3**^ **(bits)**	**Identity** ^**4**^	**Conservative substitutions** ^**5**^	**Coverage** ^**6**^
RLC_Genoveva_JROL01007197	PTREP238	TREP3154	1130	64%	78%	84%
	(1515 aa)	Copia, RLC_Olivia_42j2-1				
RLC_Carmen_JROL01007734	PTREP238	TREP3154	879	54%	67%	75%
	(1515 aa)	Copia, RLC_Olivia_42j2-1				
RLC_Facunda_JROL01000922	PTREP120	TREP2012	1154	58%	74%	85%
	(1121 aa)	Copia, RLC_Zenia_AY853252-1				
RLG_Francisca_JROL01008273	PTREP249	TREP3203	387	32%	49%	93%
	(1536 aa)	Gypsy, RLG_Latidu_10k23-1				
RLX_Victoria_JROL01006440	PTREP64	TREP99	130	37%	58%	20%
	(1717 aa)	Gypsy,RLG_Cereba_AY040832-2				
RLX_Gabrielle_JROL01007833	PTREP63	TREP98	104	32%	51%	22%
	(1520 aa)	Gypsy, RLG_Cereba_AY040832-1				

^1^code of the protein that showed the highest identity to the scaffold. Its length is indicated in parenthesis; ^2^the code and name of the retrotransposon associated with the mentioned proteins; ^3^ maximal score of the alignments expressed in bits; ^4^percentage of identity of the alignments; ^5^percentage of conservative substitutions, i.e., the aligned amino acids are not identical but both side chains have similar biochemical properties. ^6^percentage of the protein sequences that aligned with the scaffold sequence.

**Table 6:** **Description of the novel LTR retrotransposons taxonomy and family members**

**Sequence accession**	**JROL01007197**	**JROL01007734**	**JROL01000922**	**JROL01008273**	**JROL01006440**	**JROL01007833**
Family	Genoveva	Carmen	Facunda	Francisca	Victoria	Gabrielle
Superfamily	*Copia*	*Copia*	*Copia*	*Gypsy*		
Class	Retrotransposon	Retrotransposon	Retrotransposon	Retrotransposon	Retrotransposon	Retrotransposon
Order	LTR retrotransposon	LTR retrotransposon	LTR retrotransposon	LTR retrotransposon	LTR retrotransposon	LTR retrotransposon
Insertion	RLC_Genoveva_JROL01007197	RLC_Carmen_JROL01007734	RLC_Facunda_JROL01000922	RLG_Francisca_JROL01008273	RLX_Victoria_JROL01006440	RLX_Gabrielle_JROL01007833
Structural description	Autonomous retrotransposon	Autonomous retrotransposon	Autonomous retrotransposon	Autonomous retrotransposon	Non autonomous retrotransposon (TRIM)	Non autonomous retrotransposon (TRIM)
Others members	See Additional file 5: Tables S4 and Additional file 6: Table S5	See Additional file 5: Tables S4 and Additional file 6: Table S5	See Additional file 5: Tables S4 and Additional file 6: Table S5	See Additional file 5: Tables S4 and Additional file 6: Table S5	See Additional file 5: Tables S4 and Additional file 6: Table S5	See Additional file 5: Tables S4 and Additional file 6: Table S5

### Identification of members of the novel LTR retrotransposon families

The presence of full-length copies of the novel LTR retrotransposon in genome was tested, using the candidate LTR retrotransposons as probes against the *T. aestivum* chromosome arm assemblies acquired from the IWGSC database, following the criteria proposed by [6]. There were identified 21 to 757 copies for each candidate, being RLC_Facunda_JROL01000922-1 the most abundant one (Table 4).

However, such values are probably miscalculated due to the short length of the sequences deposited in the databases; thus, a single unique large LTR retrotransposon could give rise to several hits. To address this, we adopted an additional approach consisting of BLASTN searches against the *T. aestivum* (WGS project accession CALP000000000; [50]) and *Ae. tauschii* (WGS project accession AOCO000000000; [51]) whole genome shotgun sequence (wgs) databases using the six candidate LTR retrotransposons as probes. The resulting sequences were used as input for the LTR_FINDER and LTR_STRUC programs and the output sequences were extracted from the wgs and manually analyzed to verify the identity with the probed LTR retrotransposon. Such procedure allowed identification of one member of the LTR retrotransposon family for candidates RLC_Genoveva_JROL01007197-1 and RLX_Gabrielle_JROL01007833-1, two for RLC_Facunda_JROL01000922-1 and RLG_Francisca_JROL01008273-1 and three for RLC_Carmen_JROL01007734-1 (Additional file 5: Table S4). Interestingly, thirty one new LTR retrotransposons were identified when probed with RLX_Victoria_JROL01006440-1 (Additional file 5: Table S4).

Finally, all the positive hits obtained through BLASTN search of *T. aestivum* and *Ae. tauschii* wgs probed with the six candidate LTR retrotransposons were additionally BLAST searched against a local database, constructed by adding to the MIPs database the six novel LTR retrotransposons. The alignments among the wgs and the local database were manually analyzed. To be considered a candidate LTR retrotransposon copy the wgs needed to: i) show identity to the candidate exclusively or, b) exhibit remarkably higher identity to the probed LTR retrotransposon than to any other LTR retrotransposon. Sequences that fulfilled these parameters were extracted from wgs. This approach showed that at least one strong-hit copy was present in the wgs database for each of the six candidates, together with several partial copies (Table 6; Additional file 6: Table S5).

**Figure 4:**
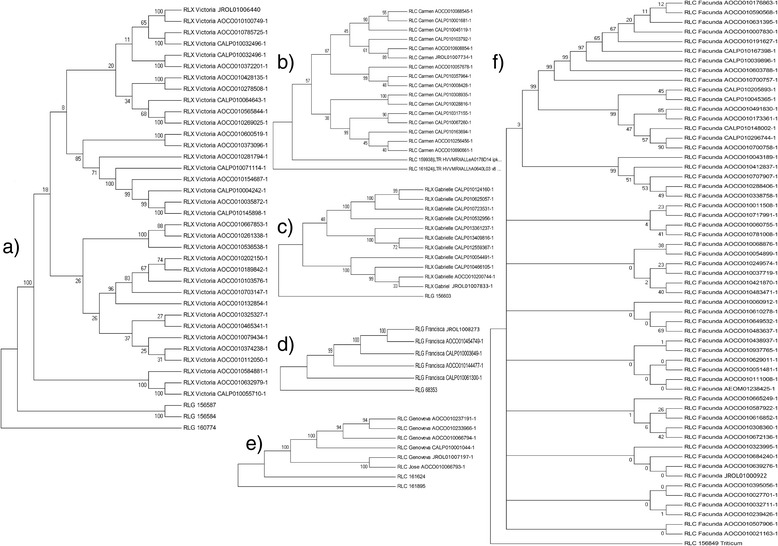
Molecular phylogenetic analysis. Evolutionary relationships among members of the six novel LTR families. *Aegilops tauschii* and *Triticum aestivum* whole genome shotgun sequences deposited at NCBI were searched using the six novel LTRs identified in wheat 4D chromosome as probes. Phylogenetic analyses were conducted in MEGA4 [75]. The number of positions that resulted parsimony informative out of the total positions in the final dataset, were **a)** 1015/1194 (Victoria), **b)** 80/82 (Carmen), **c)** 331/373 (Gabrielle), **d)** 104/1535 (Francisca), **e)** 1401/1902 (Genoveva), **f)** 313/342 (Facunda).

To elucidate the phylogenetic relationship among the members of each family, the six LTR candidates identified in the 4DS and 4DL scaffolds were individually aligned with the members of the respective families identified in wgs by the use of LTR_FINDER and LTR_STRUC and included some of the LTRs identified though BLASTN. For each candidate, the alignments included as outlayers LTRs that, in spite of being members of other families, were close to the new LTR candidates. The phylogenetic trees confirmed the existence of six new LTR retrotransposon families (Figure 4). For RLC_Carmen_JROL01007734-1 and RLC_Facunda_JROL01000922-1 there were included in the alignments the longer retrotransposons identified.

Special attention was centered in the LTR family RLX_Victoria_JROL01006440-1 since most members were identified by LTR_FINDER and/or LTR_STRUC and thus several structural information about them is available. Most of the members of the family ranged in size from 1898 to 3250 bp and carried LTRs of 120 to 1051 bp, whereas one member was 8698 bp in length. Detailed insight in such member revealed that it was not a single retrotransposon but four Victoria LTR retrotransposons in tandem. Complete elements were flanked by 4 to 6-bp target site duplications. BLASTX alignment of the members of the family with retrotransposon proteins across the GyDb and NCBI databases revealed the presence of short fragments of some proteins, such as AP and INT. Since no complete ORF could be identified, it could be deduced that the internal domains of the elements lack coding capability. Regarding the internal region, the primer binding site was complementary to the methionine tRNA in 50% of the sequences, whereas 32% corresponded to other tRNAs and it could not be identified for 18% of the tRNAs. A 15-nt polypurine tract was identified upstream of the 3′LTR. As demonstrated through BLASTX searches in the NCBI and GyDB databases, none of the identified members of the family possess the complete ORFs necessary to be considered an autonomous TE. Thus, taking into account the size of the members, the family was classified as TRIM non-autonomous LTR retrotransposons. The six novel retrotransposon families will be included in the next update of the Plant Genome and Systems Biology Repeat Element Database (PGSB-REdat).

**Figure 5:**
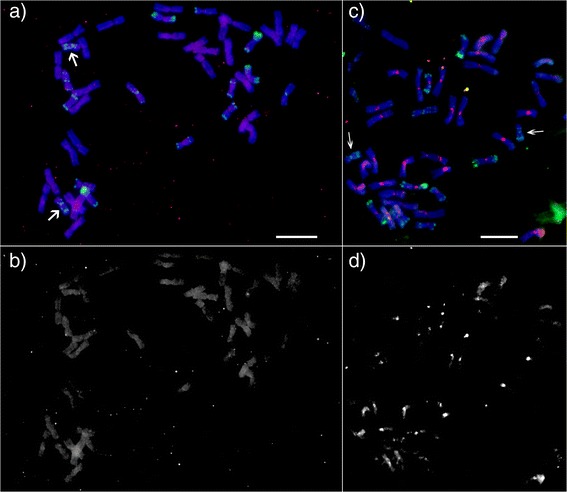
Physical localization of newly identified transposons. The identified retrotransposons were localized on metaphase spreads of vc. Chinese Spring using in situ hybridization with fluorescent labeled probes. Red color was used for the repetitive probes, Green color was used for Afa probe and Blue was stained chromosome DNA using DAPI. Arrows are pointing at 4D chromosome. **a)** Probes of all retrotransposons besides retrotransposon Carmen produced weak disperse signals on all chromosomes. The red signals represent distribution of retrotransposon Victoria. **b)** The red channel of figure A to demonstrate distribution of weak signals of Victoria probe on whole chromosomes. **c)** All probes derived from retrotransposon Carmen gave similar hybridisation pattern on metaphase chromosomes. Surprisingly, the retrotransposon was preferentially localized in centromeric region of all chromosomes with varying intensity. On few chromosomes were observed weak and dispersed signals on distal parts of chromosome arms too. **d)** The red channel of figure C to demonstrate distribution of weak signals of Carmen probe on whole chromosomes. Scale bars represent 10 μm.

The transposon insertion site based markers are specific and highly abundant, especially in large genomes where repetitive sequences represent major portions of genomic sequence, and became popular in plant genetic, physical mapping and diversity assessments. Several approaches were developed to visualize polymorphisms in the insertion sites and the most widely used in wheat are the RJM [20] or ISBP markers [18,19]. In light of this, identification and characterization of any new transposon adds to the pool of possible markers. After identification of six new LTR retrotransposons (Table 6) their DNA was amplified and labeled with fluorescent dye. The resulting probes were hybridized on metaphase chromosomes. Most of the probes (Additional file 7: Table S6) provided weak and mostly randomly distributed unreliable signals on several chromosomes (similar to Figure 5a, data not shown). An exception was probe from LTR retrotransposon Carmen which provided signal in centromeric region of all chromosomes (Figure 5b) with highly varying intensity. Unfortunately, FISH analysis could not provide quantitative data which limits assessment of abundance of the retrotransposon for centromeres of particular chromosomes. Surprisingly, chromosome 4D showed very weak signal for this probe in all metaphase figures analyzed (Figure 5c and d). These findings support previously identified facts that repetitive elements and particularly transposons can, besides their selfish multiplication, play also an important role in evolution of genomes in moderating gene expression and creating new genes by exon reshuffling [52] or are part of important genome structures as centromeres and have ability to specifically target such structures [53,54].

## Conclusion

The present work constitutes the first insight of wheat homeologous group 4 chromosomes repetitive sequences analyzed at the chromosome arm level. Detailed study of repetitive elements becomes more interesting as it has been thought before, since repetitive elements seems to play important roles in genome structure and size variation and also contribute to the evolution of genes and their function. In accordance with results obtained for other grasses, CACTA/En-Spm and *Gypsy* were the most abundant DNA transposons and retrotransposons, respectively, suggestive of their conserved roles in genome regulation. The characterization of the tandem repeat content along the homeologous group 4 allowed creating a list of SSR motifs in wheat chromosomes of the homeologous group 4. Six novel LTR retrotransposon families were characterized, including three *Copias*, one *Gypsy*, and two TRIM LTR retrotransposons. In spite of the extensive research performed in Triticeae genomes and the high number of reported elements, the fact that six new elements could be identified indicates that new families probably remain to be described. However, for more detailed study of quantitative repeatome content and structure a reference sequence is crucial.

## Methods

### Sequences from chromosome 4D

Sequences from *Triticum aestivum* cv. Chinese Spring ditelosomic (DT) lines for the 4D chromosome arms were obtained through Roche 454 sequencing technology and assembled into 8141 and 7077 scaffolds for 4DS and 4DL, respectively, hereafter named 4DS_454_ and 4DL_454_, as described in [24]. Additionally, the sequences belonging to the wheat homeologous group 4 chromosome arms obtained through Illumina sequencing technology were downloaded from the IWGSC website [21] and are referred as 4AS_I_, 4AL_I_, 4BS_I_, 4BL_I_, 4DS_I_ and 4DL_I_. When additional comparisons needed to be done, chromosome arms sequences other than those from the homeologous group 4 were also downloaded from the IWGSC website.

### Identification of repetitive elements

Repetitive sequences were identified using RepeatMasker (RM) [55]. The program inputs were FASTA-formatted archives, whereas the program output consisted of a detailed annotation of the repeats present in the query sequence. Sequence comparisons were performed using the alignment software cross_match (version open-3.3.0) [56].

From the Cross_match output list, the name of the matching interspersed repeat and the class of the repeat were used to classify and count elements belonging to SMALL RNA, satellites, simple repeats and low complexity regions using a homemade Perl script.

TE interspersed repeat family signatures were identified using Mips-REdat_v9.0p database hosted by the MIPS at PlantsDB [57], that contains ~42.000 sequences with total length of ~350 Mb. The sequences with > =95% identity over > =95% of its length were considered as redundant and only the longest element from the clusters was used for further analysis.

The repetitive element classification was performed according to hierarchy as suggested by IWGSC [58]: class, subclass and superfamily. DNA transposons were divided into subclasses based on whether they contained terminal inverted repeats (TIRs) or not. RNA retrotransposons were classified as LTR or Non-LTR retrotransposons on the basis of the presence or absence of LTRs.

### Identification and annotation of novel LTR retrotransposons

The scaffolds obtained from 4DS_454_ and 4DL_454_ sequences were scanned for LTR retrotransposons using LTR_FINDER [46] and LTR_STRUC [47]. The FASTA-formatted scaffolds from the chromosome arm database were used as input data for both programs, whereas the output consisted of putative novel LTR retrotransposon sequences. LTR_FINDER was used with default parameters with the following exceptions: the minimum LTR size was set to 100 and the minimum distance of LTRs (internal domain) was set at 1000 bp. The *Arabidopsis thaliana* (639 tRNAs; Release Feb 2004), *Brachypodium distachyon* JGI v1.08x (661 tRNAs), *Oryza sativa* (764 tRNAs), *Sorghum bicolor* version 1.0 (649 tRNAs) and *Zea mays* version 4a.53 (1168 tRNAs) databases deposited at Genomic tRNA Database [59] were used to predict the tRNA binding sites typical for LTR structure. tRNA genes prediction was performed using the program tRNAscan-SE [60]. Additional de novo LTR transposons identification was based on sequence homology independent structural features search using LTR_STRUC software [47]. The output candidate LTR retrotransposons were extracted from the scaffolds and manually inspected. Candidate LTR retrotransposons were clustered using CD-HIT (ver. 4.5.7, Jan 3 2012 [48]). The candidates were further BLAST aligned against MIPS-REdat and manually checked if they belonged to known families, using criteria proposed by Wicker et al. [6]. Two elements belong to the same family if they are at least 80% identical in at least 80% of their coding regions and internal domains, or within their LTRs, or in both. The LTRs were aligned using ClustalX [61]. Transposon-associated proteins were identified using BLASTX alignments with NCBI [62] and GyDB [49]. Annotation of LTR retrotransposons was performed according to [6]. The copy number of the candidate LTRs retrotransposons was estimated from alignments with survey sequences of all *T. aestivum* chromosome arms deposited at URGI. The alignments showing at least 80% of identity and at least 80% coverage after manually inspection were considered positive hits. Additional copies of the novel LTR retrotransposon were searched in the *T. aestivum* (WGS project accession CALP000000000; [50]) and *Ae. tauschii* (WGS project accession AOCO000000000; [51]) whole genome sequence databases deposited at NCBI.

### Estimation of insertion time

The insertion time of retrotransposons was estimated using the formula T = K/2r [63], where T, K and r are time of divergence, average number of substitutions per aligned site and average synonymous substitution rate, respectively. To estimate the divergence time of LTR retrotransposons, r was set to 1.36x10^-8^ substitutions per site per year [64]. The 5′LTR and 3′LTR of each candidate were aligned using ClustalW [61].

### Phylogenetic tree construction

Phylogenetic analyses were conducted in MEGA4 [61]. Aligned sequences were used to generate trees using the Maximum Parsimony method [65]. The bootstrap consensus tree inferred from 500 replicates [66] is taken to represent the evolutionary history of the LTR analyzed [66]. Branches corresponding to partitions reproduced in less than 50% bootstrap replicates are collapsed. The percentages of replicate trees in which the associated sequence clustered together in the bootstrap test (500 replicates) are shown next to the branches [66]. The MP tree was obtained using the Close-Neighbor-Interchange algorithm with search level 3 [65,66] in which the initial trees were obtained with the random addition of sequences (10 replicates). All positions containing gaps or missing data were eliminated from the dataset (Complete deletion option).

### *In situ* localization of newly identified and highly abundant repetitive elements

Here, it was applied fluorescent in situ hybridization labeling in suspension (FISHIS) [42] that uses an additional chromosome specific fluorescent marker which can quantitatively bind to chromosomes. The FISHIS uses microsatellite markers, but only GAA SSR proved to be applicable for reliable chromosome sorting for 12-13 out of 21 bread wheat chromosomes.

Probes for Afa repeat was labeled with digoxigenin (Roche, Mannheim, Germany) and probes for selected microsatellites (4DL_SSR1, 4DL_SSR2, 4BL_SSR1, and 4D_SSR) with Texas Red (Invitrogen, Camarillo, CA, USA) according to [36]. A 260-bp fragment of the Afa family repeat was prepared using PCR with primers AS-A and AS-B on wheat genomic DNA [67]. The SSR repeats labeled by Texas Red were prepared according to [68] using PCR primers 4DL_SSR1 – (CAGCG)_6_/(CGCTG)_3_, 4DL_SSR2 - (CCGTA)_6_/(TACGG)_4,_ 4BL_SSR1 (CGTAG)_6_/(CTACG)_4_, 4D_SSR - (TTACG)_6_/(CGTAA)_4_. The PCR amplification was carried out in a C-1000 Touch™ thermal cycler (Bio-Rad, USA) in a volume of 15 μl containing 1 μmol/l of each primer, 200 μmol/l of each of the dNTPs, but dATP is supplemented with mixture of dUTP labeled with Texas Red and dATP in ratio 1:2, 1,5 mmol/l of MgCl_2_, 0,5 U OneTaq DNA Polymerase (New England Biolabs, USA) in supplier recommended buffer. The amplification was done by 40 cycles of 30 sec at 95°C, 30 sec at 60°C, and elongation was done 30 sec at 72°C.

Probes for the newly identified transposable elements were labelled directly with Texas red (Invitrogen, Camarillo, CA, USA) using Nick translation approach [69] of PCR product from primers designed for insertion site and internal regions of the transposons. For each transposon two pairs of primers were designed (Additional file 7: Table S6). One of each primers pair was designed directly to the insertion site overlapping host sequence, TSD, and LTR sequence.

The amplicons were designed to be 0,5-4 kb long. Primers were designed using Primer3 software [70]. The amplification was carried out in a C-1000 Touch™ thermal cycler (Bio-Rad, USA) in a volume of 15 μl containing 15 ng of Chinese Spring genomic DNA, 1 μmol/l of each primer, 200 μmol/l of each of the dNTPs, 1,5 mmol/l of MgCl_2_, 0,5 U OneTaq DNA Polymerase (New England Biolabs, USA) in supplier recommended buffer. The PCR products were separated in 1% agarose gel. In case of multiple PCR products, the band of expected size was excised from agarose gel, extracted and used for labeling as described above. The identities of the PCR fragments were verified by Sanger sequencing from both corresponding primers.

Chromosome localization of the probes was performed using FISH on wheat metaphase chromosomes (cv. Chinese Spring). Chromosomes were isolated from the meristematic tissue of the root tips treated with ice water for two days and slides were prepared according to [71]. The quality of chromosome spreads was checked under the microscope and the best slides were used for FISH. Post-fixation was performed according to [72].

Hybridization mixture consisting of 40% formamide, 250 ng of calf thymus DNA, 2x SSC, 15 ng *Afa* probe, 60 ng transposable element probe and 50% dextran sulphate up to final 25 μl was applied onto the slides. The slides were denatured at 80°C for 2.5 min and incubated in humid chamber at 37°C overnight. After the hybridization, slides were stringently washed as described in [73]. The signals of Texas Red labelled probes were observed directly. Digoxigenin-labelled probes were detected using anti-digoxigenin-FITC (Roche, Mannheim, Germany) in the concentration recommended by manufacturer. Chromosome DNA was counterstained with 4′,6′-diamidino-2-phenylindole (DAPI) in Vectashield (Vector Laboratories, USA).

The preparations were evaluated using Axio Imager Z.2 Zeiss microscope (Zeiss, Oberkochen, Germany) equipped with Cool Cube 1 (Metasystems, Altlussheim, Germany) camera and appropriate filter sets. The capture of fluorescence signals and merging the layers were performed with ISIS software (Metasystems, Germany) and the final image adjustment was done in Adobe Photoshop 6.0.

### Availability of supporting data

The data sets supporting the results of this article are included within the article as Additional file 8 (File 1 and Table S7) that comprises a fasta-formatted file with the nucleotidic sequences of members of the six novel retrotransposon families sequences and table with the main features of each individual sequence. Furthermore, the six novel retrotransposon families will be included in the next update of the Plant Genome and Systems Biology Repeat Element Database (PGSB-REdat).

## Additional files

Additional file 1: Table S1. Satellites identified in the homeologous group 4 chromosome arms from *T. aestivum.*
Additional file 2: Table S2. Microsatellites identified in the homeologous group 4 chromosome arms from *T. aestivum.*
Additional file 3: Table S3. Low complexity elements identified in the homeologous group 4 chromosome arms from *T. aestivum.*
Additional file 4: Figure S1. Comparison among the size of the scaffolds obtained from 4DS454 and 4DL454 and the contigs obtained from 4DSI and 4DLI. Frequency histogram were constructed showing the sequences size in abscises and the observed frequency of each size in ordinates (JPG extension). Additional file 5: Table S4. Description of the main features of the members of the new LTR retrotransposon families identified using LTR_FINDER and/or LTR_STRUC. Additional file 6: Table S5. Novel LTR retrotransposons taxonomy and family members. Additional file 7: Table S6. PCR primers and conditions to produce FISH probes from the newly identified retrotransposons. Additional file 8:
**File 1.** Contains the fasta-formatted nucleotidic sequences of all the new LTR retrotransposon families’ members described in this manuscript (fasta extension). **Table S7.** Consist of a list of the members of the new LTR retrotransposon families detailing: the bioinformatics methodology that allowed they discovery, complementary structural features and states if they are complete or fragmented elements (Microsoft Word Document).
