# Supplementary material for: Characterization of repetitive DNA landscape in wheat homeologous group 4 chromosomes
Source: BMC Genomics. 2015 May 12;16(1):375. doi: 10.1186/s12864-015-1579-0 (PMC4440537; doi:10.1186/s12864-015-1579-0)
Supplement: Additional file 1: Table S1. — Satellites identified in the homeologous group 4 chromosome arms from T. aestivum. [file 12864_2015_1579_MOESM1_ESM.docx]

**Table S1: Satellites identified in the homeologous group 4 chromosome arms from *T. aestivum.*** Satellites were classified according to the nucleotide composition of the repetitive motif. The motifs are shown according to its frequency (#) per chromosome arm (4AS_I_, 4BS_I_, 4DS_I_, 4AL_I_, 4BL_I_, 4DL_I,_ 4DS_454_ and 4DL_454_).

| **4AS_I_** | **#** |  | **4AL_I_** | **#** |  | **4BS_I_** | **#** |  | **4BL_I_** | **#** |  | **4DS_I_** | **#** |  | **4DL_I_** | **#** |  | **4DS_454_** | **#** |  | **4DL_454_** | **#** |
| --- | --- | --- | --- | --- | --- | --- | --- | --- | --- | --- | --- | --- | --- | --- | --- | --- | --- | --- | --- | --- | --- | --- |
| TREP71 | 123 |  | TREP71 | 298 |  | TREP107 | 2248 |  | TREP107 | 1433 |  | TREP84 | 173 |  | TREP92 | 405 |  | Secale_cereale_D1100 | 7 |  | trep73 | 5 |
| TREP72 | 117 |  | TREP72 | 237 |  | TREP84 | 1104 |  | TREP84 | 883 |  | Afa_PJ | 75 |  | TREP84 | 374 |  | trep73 | 5 |  | Secale_cereale_D1100 | 4 |
| TREP84 | 110 |  | TREP13 | 221 |  | TREP71 | 145 |  | TREP72 | 127 |  | TREP71 | 71 |  | TREP71 | 191 |  | trep67 | 2 |  | trep67 | 3 |
| TREP13 | 81 |  | TREP106 | 181 |  | TREP13 | 115 |  | TREP71 | 97 |  | TREP13 | 54 |  | TREP13 | 170 |  | trep69 | 2 |  | trep69 | 3 |
| SAT-1_SB | 66 |  | TREP84 | 154 |  | TREP72 | 99 |  | SAT-1_SB | 76 |  | TREP72 | 49 |  | TREP72 | 139 |  | Beta_vulgaris_150 | 1 |  | atr0015 | 1 |
| TREP67 | 57 |  | Afa_PJ | 107 |  | SAT-1_SB | 83 |  | TREP106 | 74 |  | TREP107 | 34 |  | TREP67 | 125 |  | Hordeum_vulgare_Crep1 | 1 |  | Hordeum_vulgare_Crep1 | 1 |
| TREP107 | 56 |  | SAT-1_SB | 81 |  | TREP106 | 46 |  | TREP13 | 72 |  | Afa_LR | 24 |  | Afa_PJ | 106 |  | Hordeum_vulgare_Crep2 | 1 |  |  |  |
| TREP106 | 44 |  | TREP67 | 64 |  | Afa_LR | 24 |  | TREP38 | 65 |  | TREP106 | 22 |  | TAIL4_LR | 96 |  | Poaceae_Af | 1 |  |  |  |
| TAIL5_TA | 36 |  | TREP84 | 58 |  | TAIL4_LR | 21 |  | Afa_PJ | 52 |  | TREP67 | 16 |  | TAIL_PJ | 81 |  | Triticum_aestivum_320 | 1 |  |  |  |
| Afa_LR | 27 |  | TREP107 | 53 |  | TREP37 | 21 |  | Afa_LR | 36 |  | SAT-1_SB | 8 |  | Afa_LR | 78 |  |  |  |  |  |  |
| Afa_PJ | 21 |  | tRNASAT-1_ZM | 45 |  | TAIL5_TA | 20 |  | TREP37 | 32 |  | Afa_SC | 7 |  | TREP107 | 71 |  |  |  |  |  |  |
| TREP33 | 17 |  | Afa_LR | 38 |  | Afa_PJ | 17 |  | TAIL5_TA | 26 |  | Afa_TA | 7 |  | TREP106 | 70 |  |  |  |  |  |  |
| TAIL4_LR | 14 |  | TAIL5_TA | 26 |  | TREP33 | 15 |  | TAIL4_LR | 17 |  | TREP33 | 5 |  | SAT-1_SB | 47 |  |  |  |  |  |  |
| TAIL_TA | 7 |  | Afa_HV | 23 |  | TREP67 | 12 |  | TREP33 | 13 |  | Afa_ASQ | 4 |  | SUBTEL_sa | 37 |  |  |  |  |  |  |
| Afa_AS | 6 |  | TAIL4_LR | 13 |  | Afa_TA | 6 |  | Afa_AS | 8 |  | Afa_HV | 4 |  | TAIL_TA | 30 |  |  |  |  |  |  |
| Afa_HV | 5 |  | Afa_TU | 13 |  | Afa_TU | 5 |  | Afa_TA | 7 |  | Afa_AS | 3 |  | SPELT1_AS | 12 |  |  |  |  |  |  |
| Afa_TA | 5 |  | TREP33 | 12 |  | tRNASAT-1_ZM | 4 |  | Afa_SC | 6 |  | Afa_TD | 3 |  | Afa_TA | 11 |  |  |  |  |  |  |
| Afa_TU | 5 |  | Afa_SC | 12 |  | Afa_AS | 3 |  | Afa_TU | 6 |  | Afa_TM | 2 |  | TAIL_TU | 11 |  |  |  |  |  |  |
| TAIL_AL | 5 |  | Afa_ASQ | 12 |  | Afa_HV | 3 |  | REP2_SB | 6 |  | CENSAT_ZM | 2 |  | Afa_ASQ | 10 |  |  |  |  |  |  |
| TAIL_S | 5 |  | Afa_AS | 9 |  | TAIL_AS | 3 |  | Afa_ASQ | 4 |  | TAIL5_TA | 2 |  | tRNASAT-1_ZM | 10 |  |  |  |  |  |  |
| REP2_SB | 3 |  | REP2_SB | 8 |  | Afa_ASQ | 2 |  | Afa_HV | 4 |  | tRNASAT-1_ZM | 2 |  | REP2_SB | 9 |  |  |  |  |  |  |
| tRNASAT-1_ZM | 3 |  | TAIL_TA | 6 |  | Afa_SC | 2 |  | TREP67 | 4 |  | Afa_AC | 1 |  | Afa_SC | 8 |  |  |  |  |  |  |
| Afa_ASQ | 2 |  | Afa_TM | 6 |  | Afa_TM | 2 |  | TREP92 | 4 |  | SUBTEL_sa | 1 |  | TAIL_TM | 8 |  |  |  |  |  |  |
| CENSAT_ZM | 2 |  | Afa_TA | 6 |  | REP2_SB | 2 |  | Afa_AC | 3 |  | TAIL_AS | 1 |  | TAIL5_TA | 8 |  |  |  |  |  |  |
| TREP38 | 2 |  | Afa_TD | 5 |  | SUBTEL_sa | 2 |  | Afa_TM | 3 |  | TAIL_S | 1 |  | Afa_HV | 4 |  |  |  |  |  |  |
| TREP92 | 2 |  | TREP92 | 3 |  | TAIL_TA | 2 |  | TAIL_S | 3 |  | TAIL_TU | 1 |  | HSATII | 4 |  |  |  |  |  |  |
| Afa_AC | 1 |  | TAIL_AS | 3 |  | TREP38 | 2 |  | TAIL_TA | 3 |  | TREP37 | 1 |  | TREP33 | 4 |  |  |  |  |  |  |
| Afa_SC | 1 |  | TAIL_AL | 3 |  | TREP92 | 2 |  | TREP82 | 3 |  | TREP92 | 1 |  | Afa_AS | 3 |  |  |  |  |  |  |
| Afa_TD | 1 |  | TAIL_S | 2 |  | Afa_TD | 1 |  | Afa_TD | 2 |  |  |  |  | Afa_TU | 3 |  |  |  |  |  |  |
| SUBTEL_sa | 1 |  | TAIL_PJ | 2 |  | TAIL_LM | 1 |  | TAIL_LM | 2 |  |  |  |  | CENSAT_ZM | 3 |  |  |  |  |  |  |
| TAIL_AS | 1 |  | SUBTEL_sa | 2 |  | TAIL_PJ | 1 |  | TAIL_PJ | 2 |  |  |  |  | TAIL_ASQ | 3 |  |  |  |  |  |  |
| TAIL_PJ | 1 |  | CENSAT_ZM | 2 |  | TAIL_S | 1 |  | tRNASAT-1_ZM | 2 |  |  |  |  | Afa_AC | 2 |  |  |  |  |  |  |
| TAIL_TM | 1 |  | CENSATC4_ZM | 1 |  |  |  |  | SUBTEL_sa | 1 |  |  |  |  | TAIL_S | 2 |  |  |  |  |  |  |
|  |  |  |  |  |  |  |  |  | TAIL_AL | 1 |  |  |  |  | (CATTC)n | 1 |  |  |  |  |  |  |
|  |  |  |  |  |  |  |  |  | TAIL_TU | 1 |  |  |  |  | Afa_TM | 1 |  |  |  |  |  |  |
|  |  |  |  |  |  |  |  |  | TREP578 | 1 |  |  |  |  | CENSATC4_ZM | 1 |  |  |  |  |  |  |
|  |  |  |  |  |  |  |  |  | ZB47CEN | 1 |  |  |  |  | TAIL_AL | 1 |  |  |  |  |  |  |
|  |  |  |  |  |  |  |  |  |  |  |  |  |  |  | TAIL_AS | 1 |  |  |  |  |  |  |
|  |  |  |  |  |  |  |  |  |  |  |  |  |  |  | ZB47CEN | 1 |  |  |  |  |  |  |
