# Supplementary material for: Characterization of repetitive DNA landscape in wheat homeologous group 4 chromosomes
Source: BMC Genomics. 2015 May 12;16(1):375. doi: 10.1186/s12864-015-1579-0 (PMC4440537; doi:10.1186/s12864-015-1579-0)
Supplement: Additional file 2: Table S2. — Microsatellites identified in the homeologous group 4 chromosome arms from T. aestivum. [file 12864_2015_1579_MOESM2_ESM.docx]

**Table S2. Microsatellites identified in the homeologous group 4 chromosome arms from *T. aestivum*.** Microsatellites were classified according to the nucleotide composition of the repetitive motif. The motifs are shown according to its frequency (#) per chromosome arm (4AS_I_, 4AL_I_, 4BS_I_, 4BL_I_, 4DS_I_, 4DL_I,_ 4DS_454_ and 4DL_454_). The bolded SSRs are chromosome specific sequences and were subjected to physical localization on metaphase chromosomes using FISH.

| **4AS_I_** | **#** |  | **4AL_I_** | **#** |  | **4BS_I_** | **#** |  | **4BL_I_** | **#** |  | **4DS_I_** | **#** |  | **4DL_I_** | **#** |  | **4DS** | **#** |  | **4DL** | **#** |
| --- | --- | --- | --- | --- | --- | --- | --- | --- | --- | --- | --- | --- | --- | --- | --- | --- | --- | --- | --- | --- | --- | --- |
| (TC)n/(GA)n | 2032 |  | (GA)n/(TC)n | 2703 |  | (GA)n/(TC)n | 2706 |  | (GA)n/(TC)n | 2337 |  | (GA)n/(TC)n | 972 |  | (CCG)n/(CGG)n | 19459 |  | (TC)n/(GA)n | 170 |  | (TC)n/(GA)n | 116 |
| (CA)n/(TG)n | 1277 |  | (CA)n/(TG)n | 1828 |  | (CA)n/(TG)n | 1814 |  | (CA)n/(TG)n | 1429 |  | (CA)n/(TG)n | 679 |  | (CA)n/(TG)n | 4902 |  | (CA)n/(TG)n | 143 |  | (CA)n/(TG)n | 116 |
| (CCG)n/(CGG)n | 1120 |  | (CCG)n/(CGG)n | 1236 |  | (CCG)n/(CGG)n | 908 |  | (CCG)n/(CGG)n | 682 |  | (CCG)n/(CGG)n | 583 |  | (GA)n/(TC)n | 4009 |  | (CCG)n/(CGG)n | 94 |  | (CAA)n/(TTG)n | 39 |
| (CAA)n/(TTG)n | 599 |  | (CAA)n/(TTG)n | 687 |  | (CAG)n/(CTG)n | 800 |  | (CAG)n/(CTG)n | 609 |  | (CAG)n/(CTG)n | 473 |  | (C)n/(G)n | 2102 |  | (TA)n | 61 |  | (CCG)n/(CGG)n | 37 |
| (TA)n | 563 |  | (CAG)n/(CTG)n | 644 |  | (ATGGTG)n/(CACCAT)n | 697 |  | (ATGGTG)n/(CACCAT)n | 577 |  | (GAA)n/(TTC)n | 243 |  | (CAG)n/(CTG)n | 1852 |  | (GAA)n/(TTC)n | 51 |  | (GAA)n/(TTC)n | 37 |
| (CAG)n/(CTG)n | 551 |  | (ATG)n/(CAT)n | 633 |  | (ATG)n/(CAT)n | 623 |  | (ATG)n/(CAT)n | 514 |  | (ATG)n/(CAT)n | 216 |  | (GGA)n/(TCC)n | 1441 |  | (CAA)n/(TTG)n | 45 |  | (TA)n | 36 |
| (ATG)n/(CAT)n | 455 |  | (GAA)n/(TTC)n | 524 |  | (GAA)n/(TTC)n | 487 |  | (GAA)n/(TTC)n | 449 |  | (ATGGTG)n/(CACCAT)n | 174 |  | (CCCTAA)n/(TTAGGG)n | 829 |  | (CAT)n/(ATG)n | 38 |  | (TAA)n/(TTA)n | 26 |
| (ATGGTG)n/(CACCAT)n | 432 |  | (ATGGTG)n/(CACCAT)n | 506 |  | (CAA)n/(TTG)n | 460 |  | (CAA)n/(TTG)n | 338 |  | (CAA)n/(TTG)n | 171 |  | (CACG)n/(CGTG)n | 793 |  | (TAA)n/(TTA)n | 38 |  | (CAT)n/(ATG)n | 25 |
| (GAA)n/(TTC)n | 394 |  | (TA)n | 218 |  | (CACG)n/(CGTG)n | 359 |  | (CACG)n/(CGTG)n | 273 |  | (GGA)n/(TCC)n | 170 |  | (CCGCG)n/(CGCGG)n | 638 |  | (GGA)n/(CCT)n | 34 |  | (GGA)n/(CCT)n | 18 |
| (CCA)n/(TGG)n | 212 |  | (GGA)n/(TCC)n | 343 |  | (TA)n/(TA)n | 307 |  | (C)n/(G)n | 268 |  | (CACG)n/(CGTG)n | 160 |  | (CCA)n/(TGG)n | 600 |  | (CTG)n/(CAG)n | 29 |  | (TGG)n/(CCA)n | 15 |
| (CACG)n/(CGTG)n | 208 |  | (CCA)n/(TGG)n | 280 |  | (GGA)n/(TCC)n | 295 |  | (GGA)n/(TCC)n | 246 |  | (CGA)n/(TCG)n | 148 |  | (CGA)n/(TCG)n | 595 |  | (TGG)n/(CCA)n | 28 |  | (TAAA)n/(TTTA)n | 12 |
| (TAA)n/(TTA)n | 197 |  | (CACG)n/(CGTG)n | 271 |  | (GGGAGA)n/(TCTCCC)n | 195 |  | (GGGAGA)n/(TCTCCC)n | 176 |  | (TA)n | 121 |  | (GAA)n/(TTC)n | 584 |  | (TCTA)n/(TAGA)n | 17 |  | (TTTC)n/(GAAA)n | 12 |
| (GGA)n/(TCC)n | 179 |  | (TAGA)n/(TCTA)n | 216 |  | (CCA)n/(TGG)n | 189 |  | (CCA)n/(TGG)n | 174 |  | (CCA)n/(TGG)n | 90 |  | (TA)n | 576 |  | trep1706 | 15 |  | (CATA)n/(TATG)n | 11 |
| (CGA)n/(TCG)n | 149 |  | (CATG)n | 104 |  | (CATG)n/(CATG)n | 171 |  | (CATG)n/(CATG)n | 116 |  | (GGGAGA)n/(TCTCCC)n | 90 |  | (GAAA)n/(TTTC)n | 494 |  | (TAAAA)n/(TTTTA)n | 15 |  | (TCTA)n/(TAGA)n | 11 |
| (CTA)n/(TAG)n | 137 |  | (CGA)n/(TCG)n | 201 |  | (CTA)n/(TAG)n | 142 |  | (CTA)n/(TAG)n | 98 |  | (CTA)n/(TAG)n | 70 |  | (CCGAG)n/(CTCGG)n | 477 |  | (TAAA)n/(TTTA)n | 14 |  | (TAAAAA)n/(TTTTTA)n | 11 |
| (TAAA)n/(TTTA)n | 121 |  | (CATA)n/(TATG)n |  |  | (CGA)n/(TCG)n | 133 |  | (GAAAA)n/(TTTTC)n | 96 |  | (TAA)n/(TTA)n | 70 |  | (CCCCG)n/(CGGGG)n | 473 |  | (GAAAA)n/(TTTTC)n | 13 |  | (CTG)n/(CAG)n | 10 |
| (GGGAGA)n/(TCTCCC)n | 103 |  | (TCCA)n/(TGGA)n | 161 |  | (TAAA)n/(TTTA)n | 114 |  | (CATA)n/(TATG)n | 93 |  | (CATG)n | 57 |  | (CAA)n/(TTG)n | 455 |  | (CTA)n/(TAG)n | 12 |  | trep1706 | 10 |
| (TTAA)n/(TTAA)n | 93 |  | (GGGAGA)n/(TCTCCC)n | 156 |  | (TCCA)n/(TGGA)n | 113 |  | (CGA)n/(TCG)n | 91 |  | (TAAA)n/(TTTA)n | 47 |  | (ATG)n/(CAT)n | 393 |  | (TCCA)n/(TGGA)n | 11 |  | (TAAAA)n/(TTTTA)n | 10 |
| (ATTG)n/(CAAT)n | 89 |  | (TAAA)n/(TTTA)n | 147 |  | (CATA)n/(TATG)n | 105 |  | (TAAA)n/(TTTA)n | 89 |  | (TAAAA)n/(TTTTA)n | 39 |  | (CCTCG)n/(CGAGG)n | 386 |  | (TTTC)n/(GAAA)n | 11 |  | (G)n/(C)n | 9 |
| (CATA)n/(TATG)n | 78 |  | (CTA)n/(TAG)n | 141 |  | (GAAAA)n/(TTTTC)n | 97 |  | (CGAG)n/(CTCG)n | 85 |  | (ATTG)n/(CAAT)n | 37 |  | (CCTA)n/(TAGG)n | 359 |  | (CCCCG)n/(CGGGG)n | 11 |  | (GAAAA)n/(TTTTC)n | 9 |
| (CATG)n/(CATG)n | 78 |  | (TAA)n/(TTA)n | 107 |  | (CGAG)n/(CTCG)n | 95 |  | (TCCA)n/(TGGA)n | 84 |  | (CATA)n/(TATG)n | 37 |  | (GGGAGA)n/(TCTCCC)n | 338 |  | (CATA)n/(TATG)n | 10 |  | (CTA)n/(TAG)n | 8 |
| (TAAAA)n/(TTTTA)n | 73 |  | (GAAAA)n/(TTTTC)n | 101 |  | (CCCCG)n/(CGGGG)n | 93 |  | (TA)n/(TA)n | 76 |  | (CCCCG)n/(CGGGG)n | 37 |  | (TAAA)n/(TTTA)n | 309 |  | (CATG)n | 10 |  | (CTCG)n/(CGAG)n | 8 |
| (TAGA)n/(TCTA)n | 60 |  | (TTAA)n | 46 |  | (TTAA)n/(TTAA)n | 87 |  | (TAGA)n/(TCTA)n | 69 |  | (TAGA)n/(TCTA)n | 36 |  | (TTAA)n | 277 |  | (GGAGA)n/(TCTCC)n | 10 |  | (ATGGTG)n/(CACCAT)n | 7 |
| (GAAAA)n/(TTTTC)n | 56 |  | (ATTG)n/(CAAT)n | 79 |  | (TAA)n/(TTA)n | 79 |  | (TAAAAA)n/(TTTTTA)n | 57 |  | (CGAG)n/(CTCG)n | 35 |  | (ATGGTG)n/(CACCAT)n | 263 |  | (G)n/(C)n | 9 |  | (TCCA)n/(TGGA)n | 6 |
| (CCCCG)n/(CGGGG)n | 55 |  | (CCCCG)n/(CGGGG)n | 79 |  | (TAGA)n/(TCTA)n | 78 |  | (CAAG)n/(CTTG)n | 55 |  | (GAAAA)n/(TTTTC)n | 29 |  | (TAA)n/(TTA)n | 259 |  | (CGA)n/(TCG)n | 9 |  | (GGGGA)n/(TCCCC)n | 6 |
| (TCCA)n/(TGGA)n | 48 |  | (CATATA)n/(TATATG)n | 67 |  | (CAAG)n/(CTTG)n | 59 |  | (TAA)n/(TTA)n | 50 |  | (TAAAAA)n/(TTTTTA)n | 26 |  | (GGGA)n/(TCCC)n | 255 |  | (CAAAAA)n/(TTTTTG)n | 9 |  | (CATG)n | 5 |
| (CCGCG)n/(CGCGG)n | 41 |  | (TAAAA)n/(TTTTA)n | 63 |  | (AACTG)n/(CAGTT)n | 53 |  | (GGAGAA)n/(TTCTCC)n | 48 |  | (TCCA)n/(TGGA)n | 24 |  | (CG)n | 235 |  | (TAAAAA)n/(TTTTTA)n | 9 |  | (TTTG)n/(CAAA)n | 5 |
| (CGAG)n/(CTCG)n | 40 |  | (CAAG)n/(CTTG)n | 61 |  | (GGAGAA)n/(TTCTCC)n | 51 |  | (CCCTAA)n/(TTAGGG)n | 47 |  | (CCCTAA)n/(TTAGGG)n | 22 |  | (CGAG)n/(CTCG)n | 196 |  | (GGGAGA)n/(TCTCCC)n | 8 |  | TRSiTRTA00000006 | 5 |
| (GAAA)n/(TTTC)n | 39 |  | (CGAG)n/(CTCG)n | 55 |  | (TAAAA)n/(TTTTA)n | 47 |  | (A)n/(T)n | 44 |  | (CCGCG)n/(CGCGG)n | 22 |  | (CATG)n | 159 |  | (TCCC)n/(GGGA)n | 7 |  | (GAGAA)n/(TTCTC)n | 5 |
| (CAAG)n/(CTTG)n | 38 |  | (CCTCG)n/(CGAGG)n | 54 |  | (CCTCG)n/(CGAGG)n | 46 |  | (CCCCG)n/(CGGGG)n | 44 |  | (GGAGA)n/(TCTCC)n | 22 |  | (GGAGA)n/(TCTCC)n | 158 |  | (TTTG)n/(CAAA)n | 7 |  | (A)n/(T)n | 4 |
| (AACTG)n/(CAGTT)n | 36 |  | (CCCTAA)n/(TTAGGG)n | 53 |  | (GAAA)n/(TTTC)n | 43 |  | (GAAA)n/(TTTC)n | 39 |  | (AACTG)n/(CAGTT)n | 17 |  | (CATA)n/(TATG)n | 148 |  | (CCTCG)n/(CGAGG)n | 7 |  | (CACG)n/(CGTG)n | 4 |
| (CCCCCA)n/(TGGGGG)n | 32 |  | (CTAG)n | 26 |  | (ATTG)n/(CAAT)n | 41 |  | (ATTG)n/(CAAT)n | 38 |  | (CAAA)n/(TTTG)n | 17 |  | (AGGGGG)n/(CCCCCT)n | 145 |  | TRSiTRTA00000019 | 6 |  | (TCCC)n/(GGGA)n | 4 |
| (GGAA)n/(TTCC)n | 31 |  | (GAAA)n/(TTTC)n | 51 |  | (CCCTAA)n/(TTAGGG)n | 35 |  | (TAAAA)n/(TTTTA)n | 37 |  | (CAAAA)n/(TTTTG)n | 17 |  | (CTA)n/(TAG)n | 143 |  | TRSiTRTA00000006 | 5 |  | TRSiTRTA00000019 | 4 |
| (CATATA)n/(TATATG)n | 30 |  | (GGGGA)n/(TCCCC)n | 51 |  | (CTAG)n/(CTAG)n | 17 |  | (GGAGA)n/(TCTCC)n | 33 |  | (CAAG)n/(CTTG)n | 16 |  | (CACGA)n/(TCGTG)n | 130 |  | (CCCTAA)n/(TTAGGG)n | 5 |  | (GGAGAA)n/(TTCTCC)n | 4 |
| (CCTCG)n/(CGAGG)n | 30 |  | (AACTG)n/(CAGTT)n | 46 |  | (GGGA)n/(TCCC)n | 33 |  | (CATATA)n/(TATATG)n | 31 |  | (CAGAG)n/(CTCTG)n | 16 |  | (AGGTG)n/(CACCT)n | 128 |  | (A)n/(T)n | 4 |  | (CAATC)n/(GATTG)n | 4 |
| (TAAAAA)n/(TTTTTA)n | 30 |  | (CCGCG)n/(CGCGG)n | 46 |  | (TAAAAA)n/(TTTTTA)n | 33 |  | (GGGA)n/(TCCC)n | 31 |  | (CCTCG)n/(CGAGG)n | 16 |  | (CCCCCG)n/(CGGGGG)n | 128 |  | (CACG)n/(CGTG)n | 4 |  | (CGA)n/(TCG)n | 3 |
| (GGGGA)n/(TCCCC)n | 28 |  | (TAAAAA)n/(TTTTTA)n | 45 |  | (GGAGA)n/(TCTCC)n | 31 |  | (GGAA)n/(TTCC)n | 30 |  | (GAAA)n/(TTTC)n | 16 |  | (CAACC)n/(GGTTG)n | 119 |  | (ATGGTG)n/(CACCAT)n | 4 |  | (TTCA)n/(TGAA)n | 3 |
| (AAATG)n/(CATTT)n | 25 |  | (GGGA)n/(TCCC)n | 44 |  | (A)n/(T)n | 28 |  | (GAGAA)n/(TTCTC)n | 29 |  | (GGAA)n/(TTCC)n | 16 |  | (GGGGA)n/(TCCCC)n | 105 |  | (CAGAGA)n/(TCTCTG)n | 4 |  | (TTCC)n/(GGAA)n | 3 |
| (CCCTAA)n/(TTAGGG)n | 24 |  | (GGAGA)n/(TCTCC)n | 43 |  | (CATATA)n/(TATATG)n | 28 |  | (AACTG)n/(CAGTT)n | 26 |  | (TTAA)n | 16 |  | (CGAGA)n/(TCTCG)n | 104 |  | (CATATA)n/(TATATG)n | 4 |  | (AGGGGG)n/(CCCCCT)n | 3 |
| (CAAA)n/(TTTG)n | 23 |  | (CAAA)n/(TTTG)n | 39 |  | (GGGGA)n/(TCCCC)n | 28 |  | (CAAA)n/(TTTG)n | 25 |  | (CTAA)n/(TTAG)n | 15 |  | (ATTG)n/(CAAT)n | 99 |  | (CACCC)n/(GGGTG)n | 4 |  | (CCCTAA)n/(TTAGGG)n | 3 |
| (CAAAA)n/(TTTTG)n | 23 |  | (GGAGAA)n/(TTCTCC)n | 38 |  | (GAGAA)n/(TTCTC)n | 23 |  | (CAGC)n/(GCTG)n | 24 |  | (GGAGAA)n/(TTCTCC)n | 15 |  | (TAAAA)n/(TTTTA)n | 99 |  | (GAGAA)n/(TTCTC)n | 4 |  | (GGGAGA)n/(TCTCCC)n | 3 |
| (CAGC)n/(GCTG)n | 22 |  | (GAGAA)n/(TTCTC)n | 37 |  | (CAAA)n/(TTTG)n | 22 |  | (CCTCG)n/(CGAGG)n | 22 |  | (GGGA)n/(TCCC)n | 15 |  | (AGATG)n/(CATCT)n | 94 |  | (GGGGA)n/(TCCCC)n | 4 |  | (CACTC)n/(GAGTG)n | 3 |
| (TATAA)n/(TTATA)n | 22 |  | (GGAA)n/(TTCC)n | 36 |  | (CCGCG)n/(CGCGG)n | 22 |  | (CATAG)n/(CTATG)n | 20 |  | (CATGC)n/(GCATG)n | 14 |  | (CACCC)n/(GGGTG)n | 94 |  | (CAGC)n/(GCTG)n | 3 |  | (CCCCG)n/(CGGGG)n | 3 |
| (CAAAAA)n/(TTTTTG)n | 21 |  | (CAAAAA)n/(TTTTTG)n | 33 |  | (CG)n/(CG)n | 22 |  | (AAATG)n/(CATTT)n | 18 |  | (GGGGA)n/(TCCCC)n | 14 |  | (TAGA)n/(TCTA)n | 87 |  | (CTAA)n/(TTAG)n | 3 |  | (GGAGA)n/(TCTCC)n | 3 |
| (AGATG)n/(CATCT)n | 20 |  | (AAATG)n/(CATTT)n | 32 |  | (GGAA)n/(TTCC)n | 22 |  | (CAGAG)n/(CTCTG)n | 18 |  | (GAGAA)n/(TTCTC)n | 13 |  | (GAAAA)n/(TTTTC)n | 83 |  | (CTCA)n | 3 |  | (ATTG)n/(CAAT)n | 2 |
| (A)n/(T)n | 19 |  | (CAAAA)n/(TTTTG)n | 26 |  | (AAATG)n/(CATTT)n | 20 |  | (CATGC)n/(GCATG)n | 18 |  | (CAAAAA)n/(TTTTTG)n | 12 |  | (CAGA)n/(TCTG)n | 77 |  | (TTCC)n/(GGAA)n | 3 |  | (TTGG)n/(CCAA)n | 2 |
| (GAGAA)n/(TTCTC)n | 18 |  | (CG)n | 13 |  | (CAAAAA)n/(TTTTTG)n | 20 |  | (CAGAGA)n/(TCTCTG)n | 16 |  | (A)n/(T)n | 10 |  | (CAGG)n/(CCTG)n | 77 |  | trep106 | 3 |  | trep106 | 2 |
| (GGAGAA)n/(TTCTCC)n | 18 |  | (CAGAGA)n/(TCTCTG)n | 25 |  | (CCCCCA)n/(TGGGGG)n | 20 |  | (CCCCAA)n/(TTGGGG)n | 16 |  | (AGGGGG)n/(CCCCCT)n | 9 |  | (CAGGG)n/(CCCTG)n | 76 |  | FRSiOTOT00000010 | 3 |  | (CAAAAA)n/(TTTTTG)n | 2 |
| (GGGA)n/(TCCC)n | 17 |  | (CACTC)n/(GAGTG)n | 24 |  | (AGGGGG)n/(CCCCCT)n | 18 |  | (CCCCCA)n/(TGGGGG)n | 16 |  | (CATAG)n/(CTATG)n | 9 |  | (CACAC)n/(GTGTG)n | 75 |  | (CCCCCA)n/(GGGGGT)n | 3 |  | (CATATA)n/(TATATG)n | 2 |
| (CTAG)n/(CTAG)n | 16 |  | (CACGC)n/(GCGTG)n | 22 |  | (CAGAGA)n/(TCTCTG)n | 18 |  | (GGGGA)n/(TCCCC)n | 16 |  | (CATATA)n/(TATATG)n | 9 |  | (CACCG)n/(CGGTG)n | 73 |  | (CCCCCG)n/(CGGGGG)n | 3 |  | (CCCCCG)n/(CGGGGG)n | 2 |
| (ATATG)n/(CATAT)n | 14 |  | (CAGAG)n/(CTCTG)n | 22 |  | (CAAAA)n/(TTTTG)n | 17 |  | (TTAA)n/(TTAA)n | 15 |  | (CG)n | 9 |  | (CCCCAA)n/(TTGGGG)n | 73 |  | (CAGAG)n/(CTCTG)n | 3 |  | (AAATG)n/(CATTT)n | 2 |
| (GGAGA)n/(TCTCC)n | 14 |  | (CCCCCA)n/(TGGGGG)n | 20 |  | (CAGC)n/(GCTG)n | 17 |  | (CCCGG)n/(CCGGG)n | 15 |  | (CTAAA)n/(TTTAG)n | 9 |  | (TCCA)n/(TGGA)n | 72 |  | (CAGCC)n/(GGCTG)n | 3 |  | (AGTAG)n/(CTACT)n | 2 |
| (CACGC)n/(GCGTG)n | 13 |  | (A)n/(T)n | 19 |  | (AGATG)n/(CATCT)n | 16 |  | (TGAA)n/(TTCA)n | 15 |  | (CAGC)n/(GCTG)n | 8 |  | (CGGAG)n/(CTCCG)n | 68 |  | (CCCGG)n/(CCGGG)n | 3 |  | (ATTTG)n/(CAAAT)n | 2 |
| (CACTC)n/(GAGTG)n | 12 |  | (CAATC)n/(GATTG)n | 18 |  | (CACCC)n/(GGGTG)n | 16 |  | (CAAAA)n/(TTTTG)n | 14 |  | (CTAG)n | 8 |  | (CCCTAG)n/(CTAGGG)n | 67 |  | (CTCGG)n/(CCGAG)n | 3 |  | (CAAAA)n/(TTTTG)n | 2 |
| (AGGGGG)n/(CCCCCT)n | 12 |  | (CCCCAA)n/(TTGGGG)n | 17 |  | (CAGAG)n/(CTCTG)n | 16 |  | (CAAAAA)n/(TTTTTG)n | 14 |  | (ATCTG)n/(CAGAT)n | 7 |  | (GAGAA)n/(TTCTC)n | 61 |  | (ATTG)n/(CAAT)n | 2 |  | (CAACC)n/(GGTTG)n | 2 |
| (CAGG)n/(CCTG)n | 12 |  | (AGGGGG)n/(CCCCCT)n | 16 |  | (CAATG)n/(CATTG)n | 15 |  | (CTAG)n/(CTAG)n | 13 |  | (ATTAG)n/(CTAAT)n | 7 |  | (TATAA)n/(TTATA)n | 60 |  | (CAGG)n/(CCTG)n | 2 |  | (CACAG)n/(CTGTG)n | 2 |
| (CTAA)n/(TTAG)n | 11 |  | (CTAA)n/(TTAG)n | 15 |  | (CCCCAA)n/(TTGGGG)n | 15 |  | (AATTG)n/(CAATT)n | 13 |  | (CACGC)n/(GCGTG)n | 7 |  | (A)n/(T)n | 59 |  | (CCCA)n/(TGGG)n | 2 |  | (CACCC)n/(GGGTG)n | 2 |
| (CCCCAA)n/(TTGGGG)n | 10 |  | (CTAAA)n/(TTTAG)n | 15 |  | (CCCGG)n/(CCGGG)n | 15 |  | (CACTC)n/(GAGTG)n | 13 |  | (CAGAGA)n/(TCTCTG)n | 7 |  | (ACCTG)n/(CAGGT)n | 55 |  | (CCGG)n | 2 |  | (CACTG)n/(CAGTG)n | 2 |
| (CCCGA)n/(TCGGG)n | 10 |  | (CTTAA)n/(TTAAG)n | 15 |  | (CATAG)n/(CTATG)n | 14 |  | (CCGCG)n/(CGCGG)n | 13 |  | (TATAA)n/(TTATA)n | 7 |  | (CCCG)n/(CGGG)n | 53 |  | (CGAA)n/(TTCG)n | 2 |  | (CAGAG)n/(CTCTG)n | 2 |
| (CCCGG)n/(CCGGG)n | 10 |  | (AGATG)n/(CATCT)n | 14 |  | (CAAAC)n/(GTTTG)n | 12 |  | (AGATG)n/(CATCT)n | 12 |  | (AGATG)n/(CATCT)n | 6 |  | (TAAAAA)n/(TTTTTA)n | 53 |  | (TTAA)n | 2 |  | (CAGCC)n/(GGCTG)n | 2 |
| (ACTAG)n/(CTAGT)n | 9 |  | (CACCC)n/(GGGTG)n | 14 |  | (CCCCAG)n/(CTGGGG)n | 12 |  | (CAATC)n/(GATTG)n | 12 |  | (ATTTG)n/(CAAAT)n | 6 |  | (CCCCAG)n/(CTGGGG)n | 52 |  | atr0046 | 2 |  | (CAGGG)n/(CCCTG)n | 2 |
| (CAATA)n/(TATTG)n | 9 |  | (CACTA)n/(TAGTG)n | 14 |  | (CTAA)n/(TTAG)n | 12 |  | (CAGG)n/(CCTG)n | 12 |  | (CAGCC)n/(GGCTG)n | 6 |  | (GGAA)n/(TTCC)n | 51 |  | TRSiTRTA00000008 | 2 |  | (CTCGG)n/(CCGAG)n | 2 |
| (CACTA)n/(TAGTG)n | 9 |  | (CGGA)n/(TCCG)n | 14 |  | (CATGC)n/(GCATG)n | 11 |  | (CG)n/(CG)n | 11 |  | (CCCCCA)n/(TGGGGG)n | 6 |  | (CAAG)n/(CTTG)n | 50 |  | (AGGGGG)n/(CCCCCT)n | 2 |  | (GCTTG)n/(CAAGC)n | 2 |
| (CAACC)n/(GGTTG)n | 8 |  | (GGAAA)n/(TTTCC)n | 14 |  | (CGGAG)n/(CTCCG)n | 11 |  | (CAATG)n/(CATTG)n | 11 |  | (CCGAG)n/(CTCGG)n | 6 |  | (CAGAGA)n/(TCTCTG)n | 50 |  | (GGAGAA)n/(TTCTCC)n | 2 |  | (TATAA)n/(TTATA)n | 2 |
| (CACAG)n/(CTGTG)n | 8 |  | (ATATG)n/(CATAT)n | 13 |  | (CACGA)n/(TCGTG)n | 10 |  | (CACGA)n/(TCGTG)n | 11 |  | (CCGG)n | 6 |  | (TGAA)n/(TTCA)n | 50 |  | (AATAG)n/(CTATT)n | 2 |  | (GC)n | 1 |
| (CAGAG)n/(CTCTG)n | 8 |  | (CCCGA)n/(TCGGG)n | 13 |  | (CACTC)n/(GAGTG)n | 10 |  | (CTAA)n/(TTAG)n | 11 |  | (CCTAA)n/(TTAGG)n | 6 |  | (ACTG)n/(CAGT)n | 49 |  | (AGATG)n/(CATCT)n | 2 |  | trep71 | 1 |
| (CAGAGA)n/(TCTCTG)n | 8 |  | (TGAA)n/(TTCA)n | 13 |  | (CTAAA)n/(TTTAG)n | 10 |  | (ATATG)n/(CATAT)n | 10 |  | (CGGAG)n/(CTCCG)n | 6 |  | (CACGC)n/(GCGTG)n | 49 |  | (ATTTG)n/(CAAAT)n | 2 |  | (ACTG)n | 1 |
| (CATAG)n/(CTATG)n | 8 |  | (CAGC)n/(GCTG)n | 12 |  | (GGGAA)n/(TTCCC)n | 10 |  | (AGGGGG)n/(CCCCCT)n | 9 |  | (AGTAG)n/(CTACT)n | 5 |  | (CATATA)n/(TATATG)n | 48 |  | (CAAAA)n/(TTTTG)n | 2 |  | (CAGG)n/(CCTG)n | 1 |
| (CGAA)n/(TTCG)n | 8 |  | (CATAG)n/(CTATG)n | 12 |  | (CACGC)n/(GCGTG)n | 9 |  | (CACGC)n/(GCGTG)n | 9 |  | (CAATA)n/(TATTG)n | 5 |  | (GGAGAA)n/(TTCTCC)n | 48 |  | (CAAAG)n/(CTTTG)n | 2 |  | (CCCG)n | 1 |
| (CACCC)n/(GGGTG)n | 7 |  | (CCGG)n | 6 |  | (CAGCC)n/(GGCTG)n | 9 |  | (ATTCG)n/(CGAAT)n | 8 |  | (CACAC)n/(GTGTG)n | 5 |  | (CAGAG)n/(CTCTG)n | 41 |  | (CAATA)n/(TATTG)n | 2 |  | (CCGG)n | 1 |
| (CCCCAG)n/(CTGGGG)n | 7 |  | (CGAA)n/(TTCG)n | 12 |  | (CATAA)n/(TTATG)n | 9 |  | (CACCG)n/(CGGTG)n | 8 |  | (CGGA)n/(TCCG)n | 5 |  | (CCGAA)n/(TTCGG)n | 41 |  | (CAATG)n/(CATTG)n | 2 |  | (CGGA)n/(TCCG)n | 1 |
| (CCCCCG)n/(CGGGGG)n | 7 |  | (CTCA)n/(TGAG)n | 12 |  | (TATAA)n/(TTATA)n | 9 |  | (CATCC)n/(GGATG)n | 8 |  | (AAATG)n/(CATTT)n | 4 |  | **(CAGCG)n/(CGCTG)n** | 38 |  | (CACTC)n/(GAGTG)n | 2 |  | (CTAG)n | 1 |
| (TGAA)n/(TTCA)n | 7 |  | (CATTC)n/(GAATG)n | 11 |  | (CCGG)n/(CCGG)n | 8 |  | (CGAA)n/(TTCG)n | 8 |  | (ACTAG)n/(CTAGT)n | 4 |  | (AACTG)n/(CAGTT)n | 37 |  | (CCGCG)n/(CGCGG)n | 2 |  | (CAGA)n | 1 |
| (CAATC)n/(GATTG)n | 6 |  | (CCCGG)n/(CCGGG)n | 11 |  | (GGAAA)n/(TTTCC)n | 8 |  | (ACATG)n/(CATGT)n | 7 |  | (ATTCG)n/(CGAAT)n | 4 |  | (CCCA)n/(TGGG)n | 37 |  | (GCTTG)n/(CAAGC)n | 2 |  | (CGAT)n | 1 |
| (CACTG)n/(CAGTG)n | 6 |  | (AATTG)n/(CAATT)n | 10 |  | (CACAC)n/(GTGTG)n | 7 |  | (ACCG)n/(CGGT)n | 7 |  | (CACCC)n/(GGGTG)n | 4 |  | (CTAAA)n/(TTTAG)n | 37 |  | (GC)n | 1 |  | trep587 | 1 |
| (CAGGG)n/(CCCTG)n | 6 |  | (CAAAC)n/(GTTTG)n | 10 |  | (CACAG)n/(CTGTG)n | 7 |  | (ATTAG)n/(CTAAT)n | 7 |  | (CAGG)n/(CCTG)n | 4 |  | (CAAA)n/(TTTG)n | 35 |  | trep71 | 1 |  | GRSiTRTA00000001 | 1 |
| (CATGC)n/(GCATG)n | 6 |  | (CACAC)n/(GTGTG)n | 10 |  | (CGAA)n/(TTCG)n | 7 |  | (ATTTG)n/(CAAAT)n | 7 |  | (CCCGG)n/(CCGGG)n | 4 |  | (CCCCCA)n/(TGGGGG)n | 34 |  | (ACTG)n | 1 |  | ORSiTRTA00000003 | 1 |
| (CCGAA)n/(TTCGG)n | 6 |  | (CAGGC)n/(GCCTG)n | 10 |  | (CTCA)n/(TGAG)n | 7 |  | (CAAAC)n/(GTTTG)n | 7 |  | (CGAA)n/(TTCG)n | 4 |  | (CACAG)n/(CTGTG)n | 33 |  | (CCCG)n | 1 |  | ZRSiTRTM00000002 | 1 |
| (CG)n | 6 |  | (CCGGA)n/(TCCGG)n | 10 |  | (TGAA)n/(TTCA)n | 7 |  | (CACCC)n/(GGGTG)n | 7 |  | (CTCA)n/(TGAG)n | 4 |  | (CCCGAA)n/(TTCGGG)n | 32 |  | (CGGA)n/(TCCG)n | 1 |  | (CAGAGA)n/(TCTCTG)n | 1 |
| (CGGAG)n/(CTCCG)n | 6 |  | (CCTAA)n/(TTAGG)n | 10 |  | (ACCG)n/(CGGT)n | 6 |  | (CAGCC)n/(GGCTG)n | 7 |  | (ACCG)n/(CGGT)n | 3 |  | (CCCGA)n/(TCGGG)n | 31 |  | (CGGA)n/(TCCG)n | 1 |  | (CCCCAA)n/(TTGGGG)n | 1 |
| (GGGAA)n/(TTCCC)n | 6 |  | (CGGAG)n/(CTCCG)n | 10 |  | (ACTAG)n/(CTAGT)n | 6 |  | (CCCCCG)n/(CGGGGG)n | 7 |  | (ACTTG)n/(CAAGT)n | 3 |  | (CAGC)n/(GCTG)n | 29 |  | (CGGT)n | 1 |  | (TGGGGG)n/(CCCCCA)n | 1 |
| (AATAG)n/(CTATT)n | 5 |  | (GGGAA)n/(TTCCC)n | 10 |  | (ATTAG)n/(CTAAT)n | 6 |  | (CCCGA)n/(TCGGG)n | 7 |  | (AGTTG)n/(CAACT)n | 3 |  | (ACATG)n/(CATGT)n | 28 |  | (CTAG)n | 1 |  | (AACTG)n/(CAGTT)n | 1 |
| (AATTG)n/(CAATT)n | 5 |  | (TCCGG)n/(CCGGA)n | 10 |  | (ATTCG)n/(CGAAT)n | 6 |  | (CGGA)n/(TCCG)n | 7 |  | (ATATG)n/(CATAT)n | 3 |  | (CTAA)n/(TTAG)n | 26 |  | (CTCG)n/(CGAG)n | 1 |  | (AATTG)n/(CAATT)n | 1 |
| (ATCTG)n/(CAGAT)n | 5 |  | (ATGTG)n/(CACAT)n | 9 |  | (CAATC)n/(GATTG)n | 6 |  | (ATCG)n/(CGAT)n | 6 |  | (C)n/(G)n | 3 |  | (CAAAA)n/(TTTTG)n | 25 |  | (TTCA)n/(TGAA)n | 1 |  | (AGATG)n/(CATCT)n | 1 |
| (ATTCG)n/(CGAAT)n | 5 |  | (ATTAG)n/(CTAAT)n | 9 |  | (CACCG)n/(CGGTG)n | 6 |  | (ATCTG)n/(CAGAT)n | 6 |  | (CAAAC)n/(GTTTG)n | 3 |  | (CGAA)n/(TTCG)n | 25 |  | (TTGG)n/(CCAA)n | 1 |  | (AGGTG)n/(CACCT)n | 1 |
| (CAAAC)n/(GTTTG)n | 5 |  | (CAGCC)n/(GGCTG)n | 9 |  | (CGGA)n/(TCCG)n | 6 |  | (CATAA)n/(TTATG)n | 6 |  | (CAAGC)n/(GCTTG)n | 3 |  | (CACTC)n/(GAGTG)n | 24 |  | ORSiTRTA00000003 | 1 |  | (CAAGA)n/(TCTTG)n | 1 |
| (CACGA)n/(TCGTG)n | 5 |  | (CATGC)n/(GCATG)n | 9 |  | (ACATG)n/(CATGT)n | 5 |  | (CCCCAG)n/(CTGGGG)n | 6 |  | (CAATC)n/(GATTG)n | 3 |  | (CGGA)n/(TCCG)n | 22 |  | ORSiTRTM00000001 | 1 |  | (CACCG)n/(CGGTG)n | 1 |
| (CAGCC)n/(GGCTG)n | 5 |  | (CCAA)n/(TTGG)n | 9 |  | (ACTG)n/(CAGT)n | 5 |  | (CCTAA)n/(TTAGG)n | 6 |  | (CACCG)n/(CGGTG)n | 3 |  | (AGCTG)n/(CAGCT)n | 21 |  | TRSiTRTM00000005 | 1 |  | (CAGAT)n/(ATCTG)n | 1 |
| (CATAA)n/(TTATG)n | 5 |  | (CCGAG)n/(CTCGG)n | 9 |  | (ATATG)n/(CATAT)n | 5 |  | (CTAAA)n/(TTTAG)n | 6 |  | (CACGA)n/(TCGTG)n | 3 |  | (CAGCC)n/(GGCTG)n | 21 |  | (CCCCAA)n/(TTGGGG)n | 1 |  | (CATAT)n/(ATATG)n | 1 |
| (CTAAA)n/(TTTAG)n | 5 |  | (TATAA)n/(TTATA)n | 9 |  | (CAACC)n/(GGTTG)n | 5 |  | (CTCA)n/(TGAG)n | 6 |  | (CACTC)n/(GAGTG)n | 3 |  | (CAGCT)n/(AGCTG)n | 21 |  | (CTAGGG)n/(CCCTAG)n | 1 |  | (CATCC)n/(GGATG)n | 1 |
| (CTCA)n/(TGAG)n | 5 |  | (ATTCG)n/(CGAAT)n | 8 |  | (CAAGA)n/(TCTTG)n | 5 |  | (GGAAA)n/(TTTCC)n | 6 |  | (CCCCCG)n/(CGGGGG)n | 3 |  | (CATCC)n/(GGATG)n | 21 |  | (CTGGGG)n/(CCCCAG)n | 1 |  | (CCCGG)n/(CCGGG)n | 1 |
| (AGCTG)n/(CAGCT)n | 4 |  | (CACGA)n/(TCGTG)n | 8 |  | (CAAAG)n/(CTTTG)n | 5 |  | (CCGG)n/(CCGG)n | 5 |  | (GCCTAA)n/(TTAGGC)n | 3 |  | (CATGC)n/(GCATG)n | 21 |  | (TGGGGG)n/(CCCCCA)n | 1 |  | (CCCTA)n/(TAGGG)n | 1 |
| (AGTAG)n/(CTACT)n | 4 |  | (ATTTG)n/(CAAAT)n | 7 |  | (ACTTG)n/(CAAGT)n | 4 |  | (CAACC)n/(GGTTG)n | 5 |  | (AATAG)n/(CTATT)n | 2 |  | (GCATG)n/(CATGC)n | 21 |  | (TTAGGC)n/(GCCTAA)n | 1 |  | (CCGCG)n/(CGCGG)n | 1 |
| (ATCG)n/(CGAT)n | 4 |  | (CAGG)n/(CCTG)n | 7 |  | (AGTTG)n/(CAACT)n | 4 |  | (CACAC)n/(GTGTG)n | 5 |  | (AATTG)n/(CAATT)n | 2 |  | (GGATG)n/(CATCC)n | 21 |  | trep1709 | 1 |  | (CCTAT)n/(ATAGG)n | 1 |
| (ATGTG)n/(CACAT)n | 4 |  | (CATAA)n/(TTATG)n | 7 |  | (ATGTG)n/(CACAT)n | 4 |  | (CACTG)n/(CAGTG)n | 5 |  | (ATCG)n/(CGAT)n | 2 |  | (GGCTG)n/(CAGCC)n | 21 |  | (AAATG)n/(CATTT)n | 1 |  | (CCTCG)n/(CGAGG)n | 1 |
| (ATTTG)n/(CAAAT)n | 4 |  | (CCCCAG)n/(CTGGGG)n | 7 |  | (ATTTG)n/(CAAAT)n | 4 |  | (CCGGA)n/(TCCGG)n | 5 |  | (ATGTG)n/(CACAT)n | 2 |  | (AAATG)n/(CATTT)n | 20 |  | (AACTG)n/(CAGTT)n | 1 |  | (CTAAT)n/(ATTAG)n | 1 |
| (CACCG)n/(CGGTG)n | 4 |  | (CCCCCG)n/(CGGGGG)n | 7 |  | (CAAGG)n/(CCTTG)n | 4 |  | (GGGAA)n/(TTCCC)n | 5 |  | (CAACC)n/(GGTTG)n | 2 |  | (CAAAAA)n/(TTTTTG)n | 20 |  | (AATTG)n/(CAATT)n | 1 |  | (CTTAA)n/(TTAAG)n | 1 |
| (CATCC)n/(GGATG)n | 4 |  | (CTATA)n/(TATAG)n | 7 |  | (CAGG)n/(CCTG)n | 4 |  | (ACTAG)n/(CTAGT)n | 4 |  | (CAATG)n/(CATTG)n | 2 |  | (CACTG)n/(CAGTG)n | 20 |  | (ACCTG)n/(CAGGT)n | 1 |  | (GGAAA)n/(TTTCC)n | 1 |
| (CATTA)n/(TAATG)n | 4 |  | (ATCTG)n/(CAGAT)n | 6 |  | (CAGGC)n/(GCCTG)n | 4 |  | (CACAG)n/(CTGTG)n | 4 |  | (CAGGC)n/(GCCTG)n | 2 |  | (GGGAA)n/(TTCCC)n | 20 |  | (ACTAG)n/(CTAGT)n | 1 |  | (GGGAA)n/(TTCCC)n | 1 |
| (CCTAA)n/(TTAGG)n | 4 |  | (CAACC)n/(GGTTG)n | 6 |  | (CAGGG)n/(CCCTG)n | 4 |  | (CAGA)n/(TCTG)n | 4 |  | (CATAA)n/(TTATG)n | 2 |  | (ATCTG)n/(CAGAT)n | 19 |  | (AGCTG)n/(CAGCT)n | 1 |  | (GTCTG)n/(CAGAC)n | 1 |
| (CGGA)n/(TCCG)n | 4 |  | (CAAGC)n/(GCTTG)n | 6 |  | (CATTC)n/(GAATG)n | 4 |  | (CAGGC)n/(GCCTG)n | 4 |  | (CCCCAA)n/(TTGGGG)n | 2 |  | (CATAG)n/(CTATG)n | 18 |  | (AGGTG)n/(CACCT)n | 1 |  | (GTGTG)n/(CACAC)n | 1 |
| (CTATA)n/(TATAG)n | 4 |  | (CAATA)n/(TATTG)n | 6 |  | (CCAA)n/(TTGG)n | 4 |  | (CAGGG)n/(CCCTG)n | 4 |  | (CCCGA)n/(TCGGG)n | 2 |  | (CCTAA)n/(TTAGG)n | 18 |  | (AGTTG)n/(CAACT)n | 1 |  |  |  |
| (CTTAA)n/(TTAAG)n | 4 |  | (CATCC)n/(GGATG)n | 6 |  | (CCCA)n/(TGGG)n | 4 |  | (CCAA)n/(TTGG)n | 4 |  | (CCCTAG)n/(CTAGGG)n | 2 |  | (CCCGG)n/(CCGGG)n | 17 |  | (ATTCG)n/(CGAAT)n | 1 |  |  |  |
| (G)n/(C)n | 4 |  | (CTTA)n/(TAAG)n | 6 |  | (CCCGA)n/(TCGGG)n | 4 |  | (CGGAG)n/(CTCCG)n | 4 |  | (CGACG)n/(CGTCG)n | 2 |  | (AATTG)n/(CAATT)n | 16 |  | (CAAAC)n/(GTTTG)n | 1 |  |  |  |
| (GGAAA)n/(TTTCC)n | 4 |  | (ACCG)n/(CGGT)n | 5 |  | (CCGAG)n/(CTCGG)n | 4 |  | (ACCTG)n/(CAGGT)n | 3 |  | (CGGGA)n/(TCCCG)n | 2 |  | (ATTAG)n/(CTAAT)n | 16 |  | (CAACC)n/(GGTTG)n | 1 |  |  |  |
| (ACATG)n/(CATGT)n | 3 |  | (ACCTG)n/(CAGGT)n | 5 |  | (AGCTG)n/(CAGCT)n | 3 |  | (AGGTG)n/(CACCT)n | 3 |  | (GGGAA)n/(TTCCC)n | 2 |  | (CAAGG)n/(CCTTG)n | 16 |  | (CAAGA)n/(TCTTG)n | 1 |  |  |  |
| (ACCTG)n/(CAGGT)n | 3 |  | (ACTAG)n/(CTAGT)n | 5 |  | (AGTAG)n/(CTACT)n | 3 |  | (ATGTG)n/(CACAT)n | 3 |  | (AAGTG)n/(CACTT)n | 1 |  | (CAATG)n/(CATTG)n | 16 |  | (CAAGT)n/(ACTTG)n | 1 |  |  |  |
| (ACTG)n/(CAGT)n | 3 |  | (ACTG)n/(CAGT)n | 5 |  | (ATCTG)n/(CAGAT)n | 3 |  | (CAAGG)n/(CCTTG)n | 3 |  | (ACCTG)n/(CAGGT)n | 1 |  | (CCGG)n | 16 |  | (CAATC)n/(GATTG)n | 1 |  |  |  |
| (CAAAG)n/(CTTTG)n | 3 |  | (CAAGA)n/(TCTTG)n | 5 |  | (CAAGC)n/(GCTTG)n | 3 |  | (CAATA)n/(TATTG)n | 3 |  | (ACTCG)n/(CGAGT)n | 1 |  | (CCGGA)n/(TCCGG)n | 14 |  | (CACCG)n/(CGGTG)n | 1 |  |  |  |
| (CAATG)n/(CATTG)n | 3 |  | (CACAG)n/(CTGTG)n | 5 |  | (CAATA)n/(TATTG)n | 3 |  | (CACAA)n/(TTGTG)n | 3 |  | (ACTG)n/(CAGT)n | 1 |  | (ATATG)n/(CATAT)n | 13 |  | (CACAT)n/(ATGTG)n | 1 |  |  |  |
| (CACAC)n/(GTGTG)n | 3 |  | (CCCA)n/(TGGG)n | 5 |  | (CACTA)n/(TAGTG)n | 3 |  | (CACTA)n/(TAGTG)n | 3 |  | (AGCTG)n/(CAGCT)n | 1 |  | (CGACG)n/(CGTCG)n | 13 |  | (CAGGC)n/(GCCTG)n | 1 |  |  |  |
| (CAGA)n/(TCTG)n | 3 |  | (CCTAG)n/(CTAGG)n | 5 |  | (CACTG)n/(CAGTG)n | 3 |  | (CATTC)n/(GAATG)n | 3 |  | (AGGTG)n/(CACCT)n | 1 |  | (CTAG)n | 13 |  | (CAGGG)n/(CCCTG)n | 1 |  |  |  |
| (CGGGA)n/(TCCCG)n | 3 |  | (ACATG)n/(CATGT)n | 4 |  | (CAGTA)n/(TACTG)n | 3 |  | (AAGTG)n/(CACTT)n | 2 |  | (CAAAG)n/(CTTTG)n | 1 |  | (ATGTG)n/(CACAT)n | 12 |  | (CATCC)n/(GGATG)n | 1 |  |  |  |
| (ACCG)n/(CGGT)n | 2 |  | (AGGTG)n/(CACCT)n | 4 |  | (CCCCCG)n/(CGGGGG)n | 3 |  | (AATAG)n/(CTATT)n | 2 |  | (CAAGG)n/(CCTTG)n | 1 |  | (CAAGA)n/(TCTTG)n | 12 |  | (CGGAG)n/(CTCCG)n | 1 |  |  |  |
| (AGGTG)n/(CACCT)n | 2 |  | (AGTAG)n/(CTACT)n | 4 |  | (CTTAA)n/(TTAAG)n | 3 |  | (ACTG)n/(CAGT)n | 2 |  | (CACAA)n/(TTGTG)n | 1 |  | (CATCG)n/(CGATG)n | 12 |  | (CGTAA)n/(TTACG)n | 1 |  |  |  |
| (CAAGA)n/(TCTTG)n | 2 |  | (CAAGG)n/(CCTTG)n | 4 |  | (AATAG)n/(CTATT)n | 2 |  | (AGTAG)n/(CTACT)n | 2 |  | (CACAG)n/(CTGTG)n | 1 |  | (ACTCG)n/(CGAGT)n | 11 |  | (CTAAA)n/(TTTAG)n | 1 |  |  |  |
| (CACAA)n/(TTGTG)n | 2 |  | (CAGA)n/(TCTG)n | 4 |  | (AATTG)n/(CAATT)n | 2 |  | (CAAAG)n/(CTTTG)n | 2 |  | (CACTA)n/(TAGTG)n | 1 |  | (AGTAG)n/(CTACT)n | 11 |  | (GAATG)n/(CATTC)n | 1 |  |  |  |
| (CAGAC)n/(GTCTG)n | 2 |  | (CAGGG)n/(CCCTG)n | 4 |  | (ACTCG)n/(CGAGT)n | 2 |  | (CAACG)n/(CGTTG)n | 2 |  | (CAGA)n/(TCTG)n | 1 |  | (CAATC)n/(GATTG)n | 11 |  | (GGGAA)n/(TTCCC)n | 1 |  |  |  |
| (CAGGC)n/(GCCTG)n | 2 |  | (CCTA)n/(TAGG)n | 4 |  | (AGGTG)n/(CACCT)n | 2 |  | (CAAGC)n/(GCTTG)n | 2 |  | (CAGAA)n/(TTCTG)n | 1 |  | (CCAA)n/(TTGG)n | 11 |  | (GTGTG)n/(CACAC)n | 1 |  |  |  |
| (CATTC)n/(GAATG)n | 2 |  | (AGCTG)n/(CAGCT)n | 3 |  | (ATCG)n/(CGAT)n | 2 |  | (CAGTA)n/(TACTG)n | 2 |  | (CAGAC)n/(GTCTG)n | 1 |  | (ATTTG)n/(CAAAT)n | 10 |  |  |  |  |  |  |
| (CCAA)n/(TTGG)n | 2 |  | (ATAGG)n/(CCTAT)n | 3 |  | (CAACG)n/(CGTTG)n | 2 |  | (CAGTC)n/(GACTG)n | 2 |  | (CAGGG)n/(CCCTG)n | 1 |  | (GGAAA)n/(TTTCC)n | 9 |  |  |  |  |  |  |
| (CCCA)n/(TGGG)n | 2 |  | (C)n/(G)n | 3 |  | (CAGA)n/(TCTG)n | 2 |  | (CATTA)n/(TAATG)n | 2 |  | (CAGTA)n/(TACTG)n | 1 |  | (ACCG)n/(CGGT)n | 8 |  |  |  |  |  |  |
| (CCCGAA)n/(TTCGGG)n | 2 |  | (CAATG)n/(CATTG)n | 3 |  | (CAGGA)n/(TCCTG)n | 2 |  | (CCCA)n/(TGGG)n | 2 |  | (CATCC)n/(GGATG)n | 1 |  | (ATCG)n/(CGAT)n | 8 |  |  |  |  |  |  |
| (CCCTAG)n/(CTAGGG)n | 2 |  | (CACTG)n/(CAGTG)n | 3 |  | (CATAC)n/(GTATG)n | 2 |  | (CCCG)n/(CGGG)n | 2 |  | (CATTA)n/(TAATG)n | 1 |  | (CACTA)n/(TAGTG)n | 8 |  |  |  |  |  |  |
| (CCGAG)n/(CTCGG)n | 2 |  | (CAGAC)n/(GTCTG)n | 3 |  | (CCGAA)n/(TTCGG)n | 2 |  | (CCGAG)n/(CTCGG)n | 2 |  | (CATTC)n/(GAATG)n | 1 |  | (CAGGC)n/(GCCTG)n | 8 |  |  |  |  |  |  |
| (CCGG)n | 2 |  | (CGAGA)n/(TCTCG)n | 3 |  | (CCGGA)n/(TCCGG)n | 2 |  | (CCTA)n/(TAGG)n | 2 |  | (CCAA)n/(TTGG)n | 1 |  | (ACGTG)n/(CACGT)n | 7 |  |  |  |  |  |  |
| (CCGGA)n/(TCCGG)n | 2 |  | (CAGTC)n/(GACTG)n | 3 |  | (CCTAA)n/(TTAGG)n | 2 |  | (CGAGA)n/(TCTCG)n | 2 |  | (CCCA)n/(TGGG)n | 1 |  | (CCCTA)n/(TAGGG)n | 7 |  |  |  |  |  |  |
| (CGAAG)n/(CTTCG)n | 2 |  | (GCCTAA)n/(TTAGGC)n | 3 |  | (CGACG)n/(CGTCG)n | 2 |  | (CGGGA)n/(TCCCG)n | 2 |  | (CCCG)n/(CGGG)n | 1 |  | (CAAAC)n/(GTTTG)n | 6 |  |  |  |  |  |  |
| (CGAGA)n/(TCTCG)n | 2 |  | (CCGAA)n/(TTCGG)n | 3 |  | (CGAGA)n/(TCTCG)n | 2 |  | (CTAAG)n/(CTTAG)n | 2 |  | (CCCGAA)n/(TTCGGG)n | 1 |  | (CAATA)n/(TATTG)n | 6 |  |  |  |  |  |  |
| (CGGAA)n/(TTCCG)n | 2 |  | (CACAA)n/(TTGTG)n | 3 |  | (CTTA)n/(TAAG)n | 2 |  | (CTATA)n/(TATAG)n | 2 |  | (CCGAA)n/(TTCGG)n | 1 |  | (CACAA)n/(TTGTG)n | 6 |  |  |  |  |  |  |
| (CTAAG)n/(CTTAG)n | 2 |  | (AAGTG)n/(CACTT)n | 2 |  | (GCCTAA)n/(TTAGGC)n | 2 |  | (CTTAA)n/(TTAAG)n | 2 |  | (CCGGA)n/(TCCGG)n | 1 |  | (CAGTA)n/(TACTG)n | 6 |  |  |  |  |  |  |
| (CTTA)n/(TAAG)n | 2 |  | (AATAG)n/(CTATT)n | 2 |  | (CAGAC)n/(GTCTG)n | 1 |  | (TTAAA)n/(TTTAA)n | 2 |  | (CGAAA)n/(TTTCG)n | 1 |  | (CCTAG)n/(CTAGG)n | 6 |  |  |  |  |  |  |
| (GCCTAA)n/(TTAGGC)n | 2 |  | (ACTCG)n/(CGAGT)n | 2 |  | (CCCTAG)n/(CTAGGG)n | 1 |  | (ACTCG)n/(CGAGT)n | 1 |  | (CGTAA)n/(TTACG)n | 1 |  | (CAAGC)n/(GCTTG)n | 5 |  |  |  |  |  |  |
| (TTAAA)n/(TTTAA)n | 2 |  | (ATCG)n/(CGAT)n | 2 |  | (CTTAT)n/(ATAAG)n | 1 |  | (ACTTG)n/(CAAGT)n | 1 |  | (CTAAG)n/(CTTAG)n | 1 |  | (CATAA)n/(TTATG)n | 5 |  |  |  |  |  |  |
| (ACTTG)n/(CAAGT)n | 1 |  | (CAAAG)n/(CTTTG)n | 2 |  | (CTTTA)n/(TAAAG)n | 1 |  | (AGCTG)n/(CAGCT)n | 1 |  | (CTTA)n/(TAAG)n | 1 |  | (CTATA)n/(TATAG)n | 5 |  |  |  |  |  |  |
| (ATAAG)n/(CTTAT)n | 1 |  | (CAACG)n/(CGTTG)n | 2 |  | (GGATG)n/(CATCC)n | 1 |  | (CAAGA)n/(TCTTG)n | 1 |  | (CTTAA)n/(TTAAG)n | 1 |  | (CTTA)n/(TAAG)n | 5 |  |  |  |  |  |  |
| (ATAGG)n/(CCTAT)n | 1 |  | (CACCG)n/(CGGTG)n | 2 |  | (TAATG)n/(CATTA)n | 1 |  | (CAGAA)n/(TTCTG)n | 1 |  | (GGAAA)n/(TTTCC)n | 1 |  | (CTTAA)n/(TTAAG)n | 5 |  |  |  |  |  |  |
| (ATTAG)n/(CTAAT)n | 1 |  | (CAGGA)n/(TCCTG)n | 2 |  | (TAGG)n/(CCTA)n | 1 |  | (CAGAC)n/(GTCTG)n | 1 |  | (TGAA)n/(TTCA)n | 1 |  | (TTAAA)n/(TTTAA)n | 5 |  |  |  |  |  |  |
| (CAAGC)n/(GCTTG)n | 1 |  | (CATTA)n/(TAATG)n | 2 |  | (TAGGG)n/(CCCTA)n | 1 |  | (CATAC)n/(GTATG)n | 1 |  |  |  |  | (ACTAG)n/(CTAGT)n | 4 |  |  |  |  |  |  |
| (CAAGG)n/(CCTTG)n | 1 |  | (CCCG)n/(CGGG)n | 2 |  | (TCCCG)n/(CGGGA)n | 1 |  | (CATCG)n/(CGATG)n | 1 |  |  |  |  | (CAAAG)n/(CTTTG)n | 4 |  |  |  |  |  |  |
| (CAGAA)n/(TTCTG)n | 1 |  | (CCCTA)n/(TAGGG)n | 2 |  | (TTAAA)n/(TTTAA)n | 1 |  | (CCCGAA)n/(TTCGGG)n | 1 |  |  |  |  | (CAGAA)n/(TTCTG)n | 4 |  |  |  |  |  |  |
| (CAGTC)n/(GACTG)n | 1 |  | (CTAAG)n/(CTTAG)n | 2 |  |  |  |  | (CCCTA)n/(TAGGG)n | 1 |  |  |  |  | (CAGAC)n/(GTCTG)n | 4 |  |  |  |  |  |  |
| (CATAC)n/(GTATG)n | 1 |  | (CTTTA)n/(TAAAG)n | 2 |  |  |  |  | (CCGAA)n/(TTCGG)n | 1 |  |  |  |  | (CAGTC)n/(GACTG)n | 4 |  |  |  |  |  |  |
| (CCCTA)n/(TAGGG)n | 1 |  | (ACGTG)n/(CACGT)n | 1 |  |  |  |  | (CGAAA)n/(TTTCG)n | 1 |  |  |  |  | (CGATA)n/(TATCG)n | 4 |  |  |  |  |  |  |
| (CGAAA)n/(TTTCG)n | 1 |  | (ACTTG)n/(CAAGT)n | 1 |  |  |  |  | (CGAAG)n/(CTTCG)n | 1 |  |  |  |  | (AATAG)n/(CTATT)n | 3 |  |  |  |  |  |  |
| (CGACG)n/(CGTCG)n | 1 |  | (AGTTG)n/(CAACT)n | 1 |  |  |  |  | **(CGTAG)n/(CTACG)**n | 1 |  |  |  |  | (ACTTG)n/(CAAGT)n | 3 |  |  |  |  |  |  |
| (CGATA)n/(TATCG)n | 1 |  | (CAGTA)n/(TACTG)n | 1 |  |  |  |  | (CGTCG)n/(CGACG)n | 1 |  |  |  |  | (ATAGG)n/(CCTAT)n | 3 |  |  |  |  |  |  |
| (CTTTA)n/(TAAAG)n | 1 |  | (CCCGAA)n/(TTCGGG)n | 1 |  |  |  |  | (GCCTAA)n/(TTAGGC)n | 1 |  |  |  |  | (ATTCG)n/(CGAAT)n | 3 |  |  |  |  |  |  |
|  |  |  | (CGAAA)n/(TTTCG)n | 1 |  |  |  |  | (TATAA)n/(TTATA)n | 1 |  |  |  |  | (CATAC)n/(GTATG)n | 3 |  |  |  |  |  |  |
|  |  |  | (CGATA)n/(TATCG)n | 1 |  |  |  |  |  |  |  |  |  |  | (CGAAA)n/(TTTCG)n | 3 |  |  |  |  |  |  |
|  |  |  |  |  |  |  |  |  |  |  |  |  |  |  | (CGGGA)n/(TCCCG)n | 3 |  |  |  |  |  |  |
|  |  |  |  |  |  |  |  |  |  |  |  |  |  |  | (CTAAG)n/(CTTAG)n | 3 |  |  |  |  |  |  |
|  |  |  |  |  |  |  |  |  |  |  |  |  |  |  | (CTTTA)n/(TAAAG)n | 3 |  |  |  |  |  |  |
|  |  |  |  |  |  |  |  |  |  |  |  |  |  |  | (ATAAG)n/(CTTAT)n | 2 |  |  |  |  |  |  |
|  |  |  |  |  |  |  |  |  |  |  |  |  |  |  | (CATTA)n/(TAATG)n | 2 |  |  |  |  |  |  |
|  |  |  |  |  |  |  |  |  |  |  |  |  |  |  | (CGGAA)n/(TTCCG)n | 2 |  |  |  |  |  |  |
|  |  |  |  |  |  |  |  |  |  |  |  |  |  |  | (AAGTG)n/(CACTT)n | 1 |  |  |  |  |  |  |
|  |  |  |  |  |  |  |  |  |  |  |  |  |  |  | (CAACG)n/(CGTTG)n | 1 |  |  |  |  |  |  |
|  |  |  |  |  |  |  |  |  |  |  |  |  |  |  | (CAGGA)n/(TCCTG)n | 1 |  |  |  |  |  |  |
|  |  |  |  |  |  |  |  |  |  |  |  |  |  |  | (CATTC)n/(GAATG)n | 1 |  |  |  |  |  |  |
|  |  |  |  |  |  |  |  |  |  |  |  |  |  |  | **(CCGTA)n/(TACGG)n** | 1 |  |  |  |  |  |  |
|  |  |  |  |  |  |  |  |  |  |  |  |  |  |  | **(CGTAA)n/(TTACG**)n | 1 |  |  |  |  |  |  |
