# Supplementary material for: Characterization of repetitive DNA landscape in wheat homeologous group 4 chromosomes
Source: BMC Genomics. 2015 May 12;16(1):375. doi: 10.1186/s12864-015-1579-0 (PMC4440537; doi:10.1186/s12864-015-1579-0)
Supplement: Additional file 3: Table S3. — Low complexity elements identified in the homeologous group 4 chromosome arms from T. aestivum. [file 12864_2015_1579_MOESM3_ESM.docx]

**Table S3. Low complexity elements identified in the homeologous group 4 chromosome arms from *T. aestivum.*** Low complexity elements were classified according to the nucleotide composition of the repetitive motif. For each chromosome arm (4AS_I_, 4AL_I_, 4BS_I_, 4BL_I_, 4DS_I_, 4DL_I,_ 4DS_454_ and 4DL_454_) the motifs are sorted according to the number of times that they appear (#).

| **4AS_I_** | # |  | **4AL_I_** | # |  | **4BS_I_** | # |  | **4BL_I_** | # |  | **4DS_I_** | # |  | **4DL_I_** | # |  | **4DS_454_** | # |  | **4DL_454_** | # |
| --- | --- | --- | --- | --- | --- | --- | --- | --- | --- | --- | --- | --- | --- | --- | --- | --- | --- | --- | --- | --- | --- | --- |
| AT_rich | 7727 |  | AT_rich | 5153 |  | AT_rich | 4099 |  | AT_rich | 4958 |  | GC_rich | 18086 |  | AT_rich | 2707 |  | A-rich | 199 |  | A-rich | 144 |
| A-rich | 1507 |  | GC_rich | 901 |  | T-rich | 962 |  | GC_rich | 933 |  | AT_rich | 7474 |  | GC_rich | 798 |  | AT_rich | 542 |  | AT_rich | 525 |
| T-rich | 1473 |  | A-rich | 890 |  | A-rich | 951 |  | A-rich | 895 |  | C-rich | 2319 |  | A-rich | 534 |  | C-rich | 49 |  | C-rich | 33 |
| GC_rich | 1194 |  | GA-rich | 524 |  | GC_rich | 769 |  | T-rich | 879 |  | G-rich | 2202 |  | T-rich | 482 |  | CT-rich | 56 |  | CT-rich | 35 |
| CT-rich | 783 |  | T-rich | 942 |  | GA-rich | 509 |  | GA-rich | 622 |  | CT-rich | 1504 |  | CT-rich | 308 |  | GA-rich | 49 |  | GA-rich | 45 |
| GA-rich | 749 |  | CT-rich | 482 |  | CT-rich | 501 |  | CT-rich | 621 |  | GA-rich | 1500 |  | GA-rich | 297 |  | GC_rich | 171 |  | GC_rich | 68 |
| C-rich | 544 |  | C-rich | 391 |  | C-rich | 339 |  | C-rich | 469 |  | T-rich | 1383 |  | G-rich | 198 |  | G-rich | 42 |  | G-rich | 25 |
| G-rich | 521 |  | G-rich | 353 |  | G-rich | 336 |  | G-rich | 445 |  | A-rich | 1375 |  | C-rich | 190 |  | polypurine | 1 |  | polypyrimidine | 1 |
| Polypyrimidine | 25 |  | polypyrimidine | 22 |  | polypyrimidine | 20 |  | polypyrimidine | 16 |  | polypurine | 25 |  | polypurine | 7 |  | polypyrimidine | 4 |  | T-rich | 237 |
| Polypurine | 15 |  | polypurine | 11 |  | polypurine | 17 |  | polypurine | 9 |  | polypyrimidine | 23 |  | polypyrimidine | 3 |  | T-rich | 200 |  |  |  |
|  |  |  |  |  |  |  |  |  |  |  |  |  |  |  |  |  |  |  |  |  |  |  |
