# Supplementary figures and images for: Characterization of repetitive DNA landscape in wheat homeologous group 4 chromosomes
Source: BMC Genomics. 2015 May 12;16(1):375. doi: 10.1186/s12864-015-1579-0 (PMC4440537; doi:10.1186/s12864-015-1579-0)

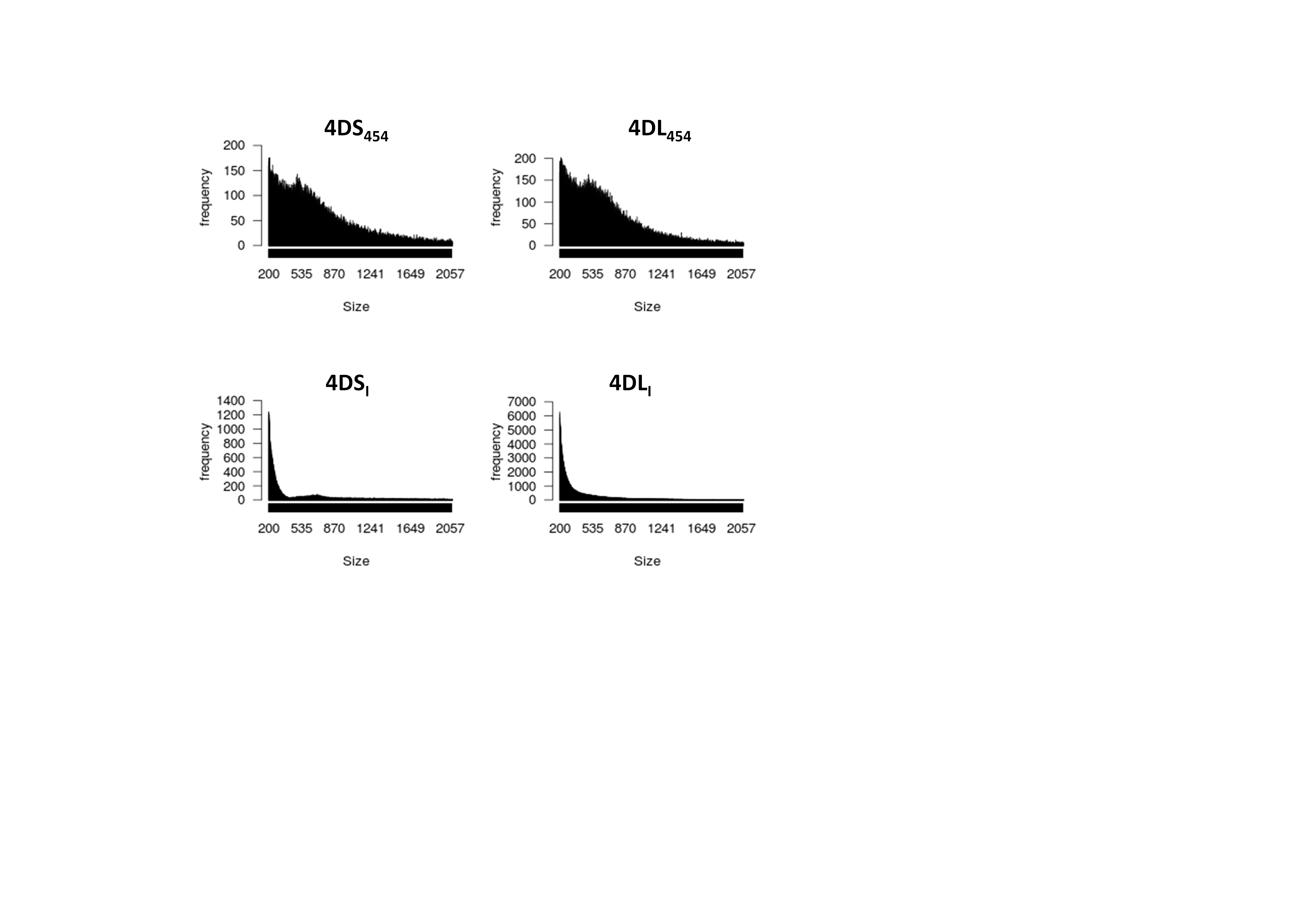

Supplement: Additional file 4: Figure S1. — Comparison among the size of the scaffolds obtained from 4DS454 and 4DL454 and the contigs obtained from 4DSI and 4DLI. Frequency histogram were constructed showing the sequences size in abscises and the observed frequency of each size in ordinates (JPG extension). [file 12864_2015_1579_MOESM4_ESM.jpg]
