# Supplementary material for: Characterization of repetitive DNA landscape in wheat homeologous group 4 chromosomes
Source: BMC Genomics. 2015 May 12;16(1):375. doi: 10.1186/s12864-015-1579-0 (PMC4440537; doi:10.1186/s12864-015-1579-0)
Supplement: Additional file 5: Table S4. — Description of the main features of the members of the new LTR retrotransposon families identified using LTR_FINDER and/or LTR_STRUC. [file 12864_2015_1579_MOESM5_ESM.docx]

**Table S4. Description of the main features of the members of the new LTR retrotransposon families identified using LTR_FINDER and/or LTR_STRUC.** TSD: target site duplication; PBS: primer binding site; PPT: polypurine tract.

| **SCAFFOLD** | **LTR retrotransposon size** | **LTR region similarity** | **5'-LTR** | **3'-LTR** | **TSD** | **orientation** | **PBS** | **PPT** |
| --- | --- | --- | --- | --- | --- | --- | --- | --- |
|  |  |  | **size** | **size** |  |  |  |  |
| RLC_Genoveva_AOCO010237191-1 | 5182 | 0.922 | 217 | 205 | TGAAT | - | - | GAGGAAGAGGGAGGC |
| RLC_Carmen_CALP010001681-1 | 5157 | 0.954 | 217 | 218 | GCTGG | - | - | GGAGGAAGAAGATGG |
| RLC_Carmen_AOCO010088545-1 | 5114 | 0.959 | 219 | 217 | CCAGC | + | MetCAT | CCATCTTCTTCCTCC |
| RLC_Carmen_AOCO010057678-1 | 5172 | 1 | 225 | 225 | ATTCT | + | ArgTCT | CCTTCTTCTTCCTCT |
| RLC_Facunda_AOCO010288406-1 | 2699 | 0.896 | 130 | 140 | CCTAT | - | MetCAT | ATTGATACTGCGCGCGGGAGGGGGGG |
| RLG_Francisca_AOCO010454749-1 | 5279 | 0.966 | 321 | 321 | CTGTC | + | SerGCT | TCTCCTGGTCCTCCC |
| RLG_Francisca_AOCO010144477-1 | 5065 | 0.959 | 319 | 320 | CAAAT | - | SerGCT | GGGAGGACCAGGAGA |
| RLX_Gabrielle_AOCO010200744-1 | 2644 | 0.934 | 680 | 685 | ACATT | - | MetCAT | ATAGCTTCGTTCCAAGAAGGAGGGGA |
| RLX_Victoria_AOCO010100749-1 | 1939 | 0.955 | 419 | 419 | ACAAG | + | MetCAT | TGCACCCCCTTCTCC |
| RLX_Victoria_AOCO010785725-1 | 2990 | 0.974 | 938 | 939 | AGAGA | - | MetCAT | TCAAGGAAAGGAGGA |
| RLX_Victoria_CALP010032496-1 | 2775 | 0.94 | 766 | 768 | NOT FOUND | + | SerGCT | GCCACCTTCTTCTCT |
| RLX_Victoria_AOCO010317797-1 | 3009 | 0.969 | 940 | 940 | NOT FOUND | - | MetCAT | AGGAAGGGAGGATGA |
| RLX_Victoria_CALP010004242-1 | 2803 | 0.973 | 849 | 850 | GGGGC | - | MetCAT | TCAAGGGCGAGAGGA |
| RLX_Victoria_AOCO010035872-1 | 2829 | 0.965 | 860 | 860 | AAAA | - | MetCAT | TCAAGGGCGAGAGGA |
| RLX_Victoria_AOCO010202150-1 | 8698 | 0.948 | 2512 | 2527 | GTTT | - | LysCTT | GGAAGGGCACGTGAA |
| RLX_Victoria_AOCO010325327-1 | 3046 | 0.966 | 968 | 967 | AGAGG | - | GlnTTG | TCAAGGAAGGGAGGA |
| RLX_Victoria_AOCO010103576-1 | 3062 | 0.973 | 978 | 978 | CGCGG | + | HisGTG | TCAAGGAAGGGAGGA |
| RLX_Victoria_AOCO010132854-1 | 2895 | 0.957 | 894 | 890 | GGTGA | - | MetCAT | TCAACGGAGGAAGGA |
| RLX_Victoria_AOCO010565844-1 | 3147 | 0.928 | 1038 | 997 | CTTGT | - | MetCAT | TCAAGGAAGGGAGGA |
| RLX_Victoria_CALP010145898-1 | 2087 | 0.951 | 122 | 120 | NOT FOUND | + | ProCGG | TTTTTCTGTATATCT |
| RLX_Victoria_AOCO010703147-1 | 3068 | 0.984 | 978 | 978 | AAACG | - | TyrGTA | TCAAGGAAGGGAGGA |
| RLX_Victoria_AOCO010667853-1 | 2918 | 0.972 | 903 | 904 | CCCGC | - | MetCAT | TGAAGAGAGGGAGGA |
| RLX_Victoria_AOCO010632979-1 | 2346 | 0.968 | 219 | 219 | NOT FOUND | - | ThrCGT | AACAACACAGTAAAA |
| RLX_Victoria_AOCO010269025-1 | 3112 | 0.972 | 996 | 989 | CGCTA | + | MetCAT | TCCTCCCTTCCTTGA |
| RLX_Victoria_AOCO010281794-1 | 2671 | 0.95 | 807 | 801 | AGTGC | + | MetCAT | TTTTCTGCTCCGCCT |
| RLX_Victoria_AOCO010465341-1 | 3022 | 0.969 | 956 | 955 | TGGCC | + | MetCAT | TCCTCCCTCCCTTCA |
| RLX_Victoria_CALP010055710-1 | 2682 | 0.973 | 588 | 587 | NOT FOUND | + | MetCAT | TCCTCCCTCCCTTCA |
| RLX_Victoria_AOCO010584881-1 | 3106 | 0.971 | 1003 | 1003 | TCACA | - | MetCAT | TGAAGCGAGGGAGGA |
| RLX_Victoria_AOCO010536538-1 | 2896 | 0.955 | 906 | 896 | CCACC | + | MetCAT | TCCTCCCTCTCTTCA |
| RLX_Victoria_AOCO010374238-1 | 3078 | 0.934 | 981 | 988 | TAGGA | + | MetCAT | TCCCTCCCTTGAAGC |
| RLX_Victoria_AOCO010428135-1 | 3032 | 0.95 | 974 | 972 | CACCT | - | MetCAT | TGAAGGAAGGGAGGA |
| RLX_Victoria_AOCO010600519-1 | 2448 | 0.955 | 672 | 671 | NOT FOUND | - | MetCAT | TCAAGAAAGGGAGGA |
| RLX_Victoria_AOCO010189842-1 | 2816 | 0.959 | 874 | 871 | AAGGT | + | HisGTG | TCCTCCCTTCCTTGA |
| RLX_Victoria_AOCO010154687-1 | 2787 | 0.953 | 857 | 848 | ACTTA | - | MetCAT | TCAAGGGCGAGAGGA |
| RLX_Victoria_AOCO010079434-1 | 3250 | 0.941 | 956 | 953 | AGAGAG | - | AsnGTT | TGAAGGGAGGAAGGA |
| RLX_Victoria_AOCO010373096-1 | 3096 | 0.968 | 994 | 991 | GAGAC | - | SerAGA | TCAAGAAAGGGAGGA |
| RLX_Victoria_CALP010071114-1 | 2184 | 0.937 | 229 | 237 | NOT FOUND | + | TyrATA | TCTTGTGTATCATTT |
| RLX_Victoria_AOCO010261338-1 | 2633 | 0.952 | 614 | 618 | GTTT | - | ArgCCG | GAGAGGAAGGTTATA |
| RLX_Victoria_AOCO010112050-1 | 3078 | 0.934 | 981 | 988 | TAGGA | + | MetCAT | TCCCTCCCTTGAAGC |
