# Supplementary material for: Characterization of repetitive DNA landscape in wheat homeologous group 4 chromosomes
Source: BMC Genomics. 2015 May 12;16(1):375. doi: 10.1186/s12864-015-1579-0 (PMC4440537; doi:10.1186/s12864-015-1579-0)
Supplement: Additional file 6: Table S5. — Novel LTR retrotransposons taxonomy and family members. [file 12864_2015_1579_MOESM6_ESM.docx]

**Table S5. Novel LTR retrotransposons taxonomy and family members.**

| **LTR**  **retrotransposon** | **RLC_Genoveva_JROL01007197** | **RLC_Carmen_JROL01007734** | **RLC_Facunda_JROL01000922** | **RLG_Francisca_JROL01008273** |
| --- | --- | --- | --- | --- |
| Other members (software) | RLC_Genoveva_AOCO010237191-1 | RLC_Carmen_CALP010001681-1  RLC_Carmen_AOCO010088545-1  RLC_Carmen_AOCO010057678-1 | RLC_Facunda_AOCO010288406-1 | RLG_Francisca_AOCO010454749-1  RLG_Francisca_AOCO010144477-1 |
| Other members (wgs BLAST) | RLC_Genoveva_AOCO010066794-1  RLC_Genoveva_AOCO010066793-1  RLC_Genoveva_AOCO010233966-1  RLC_Genoveva_CALP010001044-1 | RLC_Carmen_AOCO010608854-1  RLC_Carmen_AOCO010690661-1  RLC_Carmen_CALP010028816-1  RLC_Carmen_AOCO010256456-1  RLC_Carmen_CALP010306555-1  RLC_Carmen_CALP010308935-1  RLC_Carmen_CALP010045119-1  RLC_Carmen_CALP011468448-1  RLC_Carmen_CALP011191886-1  RLC_Carmen_CALP010103792-1  RLC_Carmen_CALP010067260-1  RLC_Carmen_CALP010446884-1  RLC_Carmen_CALP010317155-1  RLC_Carmen_CALP010163694-1  RLC_Carmen_CALP010008428-1  RLC_Carmen_CALP010357964-1  RLC_Carmen_CALP011255795-1  RLC_Carmen_CALP011598660-1  RLC_Carmen_CALP010236408-1  RLC_Carmen_CALP011765918-1  RLC_Carmen_CALP010332330-1  RLC_Carmen_AOCO010608853-1  RLC_Carmen_CALP011001485-1  RLC_Carmen_CALP011112138-1  RLC_Carmen_CALP011000205-1  RLC_Carmen_CALP010121574-1  RLC_Carmen_CALP012884601-1  RLC_Carmen_CALP011662724-1  RLC_Carmen_CALP011659433-1  RLC_Carmen_CALP012348547-1  RLC_Carmen_CALP012055131-1  RLC_Carmen_CALP010644204-1  RLC_Carmen_CALP010464375-1  RLC_Carmen_CALP010224113-1  RLC_Carmen_CALP012501138-1  RLC_Carmen_CALP013294378-1  RLC_Carmen_AOCO010440397-1  RLC_Carmen_CALP011828547-1  RLC_Carmen_CALP010165878-1  RLC_Carmen_CALP011654708-1  RLC_Carmen_CALP011233976-1  RLC_Carmen_CALP013303800-1  RLC_Carmen_CALP012552884-1  RLC_Carmen_AOCO010339139-1  RLC_Carmen_AOCO010690664-1  RLC_Carmen_CALP010889185-1  RLC_Carmen_CALP012239291-1  RLC_Carmen_AOCO010339138-1  RLC_Carmen_CALP010093698-1  RLC_Carmen_CALP010736972-1 | RLC_Facunda_AOCO010338758-1  RLC_Facunda_AOCO010323995-1  RLC_Facunda_CALP010045365-1  RLC_Facunda_CALP010039896-1  RLC_Facunda_AOCO010700758-1  RLC_Facunda_AOCO010700757-1  RLC_Facunda_AOCO010483637-1  RLC_Facunda_AOCO010021163-1  RLC_Facunda_AOCO010639276-1  RLC_Facunda_CALP010148002-1  RLC_Facunda_AOCO010173361-1  RLC_Facunda_AOCO010507906-1  RLC_Facunda_CALP010167398-1  RLC_Facunda_AOCO010438937-1  RLC_Facunda_AOCO010631395-1  RLC_Facunda_AOCO010590568-1  RLC_Facunda_AOCO010421870-1  RLC_Facunda_AOCO010672136-1  RLC_Facunda_AOCO010051481-1  RLC_Facunda_CALP010205893-1  RLC_Facunda_AOCO010239426-1  RLC_Facunda_AOCO010491830-1  RLC_Facunda_AOCO010665249-1  RLC_Facunda_AOCO010717991-1  RLC_Facunda_AOCO010781008-1  RLC_Facunda_AOCO010027701-1  RLC_Facunda_AOCO010054899-1  RLC_Facunda_AOCO010037719-1  RLC_Facunda_AOCO010060755-1  RLC_Facunda_AOCO010308360-1  RLC_Facunda_AOCO010032711-1  RLC_Facunda_AOCO010060912-1  RLC_Facunda_AOCO010395056-1  RLC_Facunda_AOCO010684240-1  RLC_Facunda_AEOM01238425-1  RLC_Facunda_AOCO010616852-1  RLC_Facunda_AOCO010043189-1  RLC_Facunda_AOCO010649532-1  RLC_Facunda_AOCO010707907-1  RLC_Facunda_AOCO010111008-1  RLC_Facunda_AOCO010176863-1  RLC_Facunda_AOCO010587922-1  RLC_Facunda_AOCO010412837-1  RLC_Facunda_AOCO010603788-1  RLC_Facunda_AOCO010629011-1  RLC_Facunda_CALP010296744-1  RLC_Facunda_AOCO010249574-1  RLC_Facunda_AOCO010610278-1  RLC_Facunda_AOCO010068876-1  RLC_Facunda_AOCO010007830-1  RLC_Facunda_AOCO010011508-1  RLC_Facunda_AOCO010191627-1  RLC_Facunda_AOCO010292561-1  RLC_Facunda_AOCO010841206-1  RLC_Facunda_CALP010342614-1  RLC_Facunda_CALP010431080-1  RLC_Facunda_CALP010435951-1  RLC_Facunda_AOCO010021571-1  RLC_Facunda_AOCO010636304-1  RLC_Facunda_AOCO010582471-1  RLC_Facunda_AOCO010716822-1  RLC_Facunda_AOCO010653612-1  RLC_Facunda_AOCO010636834-1  RLC_Facunda_CALP010480293-1  RLC_Facunda_AOCO010025613-1  RLC_Facunda_AOCO010317688-1  RLC_Facunda_AOCO010570347-1  RLC_Facunda_AOCO010674073-1  RLC_Facunda_AOCO010778529-1  RLC_Facunda_CALP010527190-1  RLC_Facunda_AOCO010821728-1  RLC_Facunda_AOCO010590567-1  RLC_Facunda_AOCO010498211-1  RLC_Facunda_AOCO010508171-1  RLC_Facunda_AOCO010239865-1  RLC_Facunda_AOCO010692732-1  RLC_Facunda_CALP010166116-1  RLC_Facunda_AOCO010237062-1  RLC_Facunda_AOCO010021572-1  RLC_Facunda_AOCO010339256-1  RLC_Facunda_AOCO010105934-1  RLC_Facunda_AOCO010545762-1  RLC_Facunda_AOCO010688677-1  RLC_Facunda_AOCO010937765-1  RLC_Facunda_AOCO010134283-1  RLC_Facunda_AOCO010483471-1  RLC_Facunda_AOCO010503571-1  RLC_Facunda_AOCO010282311-1  RLC_Facunda_AOCO010632460-1  RLC_Facunda_CALP010660128-1  RLC_Facunda_AOCO010283993-1  RLC_Facunda_AOCO010916949-1  RLC_Facunda_AOCO010803563-1  RLC_Facunda_AOCO010579688-1  RLC_Facunda_AOCO010201733-1  RLC_Facunda_AOCO010120669-1  RLC_Facunda_AOCO010292560-1  RLC_Facunda_AOCO010317687-1  RLC_Facunda_AOCO010664168-1  RLC_Facunda_AOCO010381216-1  RLC_Facunda_CALP010689395-1  RLC_Facunda_AOCO010325142-1  RLC_Facunda_AOCO010438938-1  RLC_Facunda_AOCO010552457-1  RLC_Facunda_AOCO010668208-1  RLC_Facunda_AOCO010788709-1  RLC_Facunda_AOCO010215994-1  RLC_Facunda_AOCO010392677-1  RLC_Facunda_AOCO010379024-1  RLC_Facunda_CALP010775656-1  RLC_Facunda_CALP010778523-1  RLC_Facunda_AOCO010416663-1  RLC_Facunda_CALP010781314-1  RLC_Facunda_AOCO010799006-1  RLC_Facunda_AOCO010228532-1  RLC_Facunda_AOCO010237773-1  RLC_Facunda_AOCO010239784-1  RLC_Facunda_AOCO010284354-1  RLC_Facunda_AOCO010145279-1  RLC_Facunda_AOCO010175032-1  RLC_Facunda_AOCO010481068-1  RLC_Facunda_AOCO010622941-1  RLC_Facunda_AOCO010142555-1  RLC_Facunda_AOCO010672444-1  RLC_Facunda_AOCO010340314-1  RLC_Facunda_CALP010833900-1  RLC_Facunda_AOCO010205433-1  RLC_Facunda_AOCO010665250-1  RLC_Facunda_AOCO010296411-1  RLC_Facunda_AOCO010430070-1  RLC_Facunda_AOCO010609284-1  RLC_Facunda_CALP010862251-1  RLC_Facunda_AOCO010285134-1  RLC_Facunda_CALP010877442-1  RLC_Facunda_CALP010881281-1  RLC_Facunda_AOCO010196662-1  RLC_Facunda_AOCO010558470-1  RLC_Facunda_CALP010889023-1  RLC_Facunda_AOCO010184525-1  RLC_Facunda_AOCO010864958-1  RLC_Facunda_AOCO010732959-1  RLC_Facunda_AOCO010121158-1  RLC_Facunda_AOCO010325070-1  RLC_Facunda_AOCO010864957-1  RLC_Facunda_CALP010657372-1  RLC_Facunda_AOCO010320908-1  RLC_Facunda_AOCO010471507-1  RLC_Facunda_CALP010951044-1  RLC_Facunda_AOCO010803562-1  RLC_Facunda_CALP010968694-1  RLC_Facunda_CALP010981426-1  RLC_Facunda_AOCO010315091-1  RLC_Facunda_AEOM01280901-1  RLC_Facunda_CALP011003546-1  RLC_Facunda_AOCO010055959-1  RLC_Facunda_AOCO010531973-1  RLC_Facunda_AOCO010278615-1  RLC_Facunda_AOCO010364409-1  RLC_Facunda_AOCO010148031-1  RLC_Facunda_AOCO010682987-1  RLC_Facunda_AOCO010483387-1  RLC_Facunda_AOCO010392678-1  RLC_Facunda_AOCO010337734-1  RLC_Facunda_CALP011206695-1  RLC_Facunda_CALP010246332-1  RLC_Facunda_AOCO010306757-1  RLC_Facunda_AOCO010292158-1  RLC_Facunda_AOCO010503007-1  RLC_Facunda_AOCO010496849-1  RLC_Facunda_AOCO010778235-1  RLC_Facunda_AOCO010674074-1  RLC_Facunda_CALP011356663-1  RLC_Facunda_AOCO010105933-1  RLC_Facunda_AOCO010438322-1  RLC_Facunda_AOCO010056355-1  RLC_Facunda_AOCO010489717-1  RLC_Facunda_AOCO010067994-1  RLC_Facunda_AOCO010059447-1  RLC_Facunda_AOCO010501503-1  RLC_Facunda_CALP011056667-1  RLC_Facunda_AOCO010652134-1  RLC_Facunda_CALP011457748-1  RLC_Facunda_AOCO010383957-1  RLC_Facunda_AOCO010333244-1  RLC_Facunda_CALP010253422-1  RLC_Facunda_CALP010704921-1  RLC_Facunda_AOCO010210244-1  RLC_Facunda_CALP011540241-1  RLC_Facunda_AOCO010769610-1  RLC_Facunda_AOCO010041712-1  RLC_Facunda_CALP011623892-1  RLC_Facunda_AOCO010379179-1  RLC_Facunda_CALP011680601-1  RLC_Facunda_AOCO010811368-1 | RLG_Francisca_AEOM01028590-1  RLG_Francisca_AEOM01048149-1  RLG_Francisca_AEOM01071161-1  RLG_Francisca_AEOM01094980-1  RLG_Francisca_CALP010003649-1  RLG_Francisca_CALP010061300-1  RLG_Francisca_CALP010090108-1  RLG_Francisca_CALP010153829-1  RLG_Francisca_CALP010289309-1  RLG_Francisca_CALP010296358-1  RLG_Francisca_CALP010313935-1  RLG_Francisca_CALP010450630-1  RLG_Francisca_CALP010532755-1  RLG_Francisca_CALP010535643-1  RLG_Francisca_CALP010594168-1  RLG_Francisca_CALP010619063-1  RLG_Francisca_CALP010635319-1  RLG_Francisca_CALP010691093-1  RLG_Francisca_CALP010869653-1  RLG_Francisca_CALP010903443-1  RLG_Francisca_CALP011534613-1  RLG_Francisca_CALP011637278-1  RLG_Francisca_CALP011637439-1  RLG_Francisca_CALP011856515-1  RLG_Francisca_CALP012053594-1  RLG_Francisca_CALP012062839-1  RLG_Francisca_CALP012632193-1  RLG_Francisca_CALP013495848-1  RLG_Francisca_CALP013648311-1  RLG_Francisca_CALP013685092-1  RLG_Francisca_CALP013813545-1 |

| **LTR retrotransposon** | **RLX_Victoria_JROL01006440** | **RLX_Gabrielle_JROL01007833** |
| --- | --- | --- |
| Other members  (software) | See Supplementary Table 5 | RLC_Gabrielle_AOCO010200744-1 |
| Other members  (wgs BLAST) | RLX_Victoria_CALP010064643-1  RLX_Victoria_AOCO010372201-1  RLX_Victoria_AOCO010278508-1 | RLC_Gabrielle_CALP010466105-1  RLC_Gabrielle_CALP010124160-1  RLC_Gabrielle_CALP010723531-1  RLC_Gabrielle_CALP013409816-1  RLC_Gabrielle_CALP012559367-1  RLC_Gabrielle_CALP013361237-1  RLC_Gabrielle_CALP010625057-1  RLC_Gabrielle_CALP010054491-1  RLC_Gabrielle_CALP010532956-1 |
