# Supplementary material for: Characterization of repetitive DNA landscape in wheat homeologous group 4 chromosomes
Source: BMC Genomics. 2015 May 12;16(1):375. doi: 10.1186/s12864-015-1579-0 (PMC4440537; doi:10.1186/s12864-015-1579-0)
Supplement: Additional file 7: Table S6. — PCR primers and conditions to produce FISH probes from the newly identified retrotransposons. [file 12864_2015_1579_MOESM7_ESM.docx]

**Table S6. PCR primers and conditions to produce FISH probes from the newly identified retrotransposons.**

| **Primer pair** | **Primer seq.** | **Optimal Ta [°C]** | **Amplicon size [bp]** |
| --- | --- | --- | --- |
| Francisca_F2/R2 | TTTTGCTGCCAAGCTGTATG GGCGACAGTGTCAGCGACT | 66 | 3385 |
| Facunda_F2/R2 | GGGCTTGTTCAAGCATGG AGTTGTGTTCGGCaGGTAGG | 60 | 1768 |
| Genoveva_F2/R1 | TCAATTATTGCAACCCCTCA CCTGAATACCCATTTGCAATC | 61 | 2001 |
| Victoria_F1/R1 | CAAGTGATGAAGCACGGACT TCAACATGGTGAGCATCGAG | 68 | 1352 |
| Victoria_F2/R2 | TTAAATTACCACTCATCACTTTCATTG CTCACTCAAACTATGGTACAAAGAACT | 63 | 1520 |
| Carmen_F1/R2 | GGGCACCTGTGTTGGAAATA GGGGCACATAAAATGACCAC | 65 | 2998 |
| Carmen_F3/R2 | ACTACTTGCTGCTCCCTCCA  GGGGCACATAAAATGACCAC | 65 | 553 |
