# Supplementary material for: Characterization of repetitive DNA landscape in wheat homeologous group 4 chromosomes
Source: BMC Genomics. 2015 May 12;16(1):375. doi: 10.1186/s12864-015-1579-0 (PMC4440537; doi:10.1186/s12864-015-1579-0)
Supplement: Additional file 8: — File 1. Contains the fasta-formatted nucleotidic sequences of all the new LTR retrotransposon families’ members described in this manuscript (fasta extension). Table S7. Consist of a list of the members of the new LTR retrotransposon families detailing: the bioinformatics methodology that allowed they discovery, complementary structural features and states if they are complete or fragmented elements (Microsoft Word Document). [file 12864_2015_1579_MOESM8_ESM.docx]

| **Identified by** | **Name** | **Length** | **5’LTR** | **3’LTR** | **TSD** | **CDS** | **Comment** |
| --- | --- | --- | --- | --- | --- | --- | --- |
| LTR finder | RLC_Carmen_JROL00000000 | 4597 | 1 - 131 | 4467 - 4597 | - | 298 - 1111 | Complete element |
| LTR finder | RLC_Carmen_AOCO010057678-1 | 5172 | 1 - 225 | 4948 - 5172 | ATTCT | 375 - 4951 | Complete element |
| LTR finder | RLC_Carmen_AOCO010088545-1 | 5114 | 1 - 217 | 4896 - 5114 | CCAGC | 393 - 4899 | Complete element |
| LTR finder | RLC_Carmen_CALP010001681-1 | 5157 | 1 - 217 | 4940 - 5157 | GCTGG | 399 - 4898 | Complete element |
| BLAST search | RLC_Carmen_AOCO010339138-1 | 2714 | - | - | - | 154 - 2710 | Fragmented element |
| BLAST search | RLC_Carmen_AOCO010339139-1 | 842 | - | - | - | 117 - 742 | Fragmented element |
| BLAST search | RLC_Carmen_AOCO010440397-1 | 1473 | - | - | - | 37 - 1472 | Fragmented element |
| BLAST search | RLC_Carmen_CALP010008428-1 | 2166 | - | - | - | 148 - 1574 | Fragmented element |
| BLAST search | RLC_Carmen_CALP010045119-1 | 1836 | - | - | - | 10 - 1792 | Fragmented element |
| BLAST search | RLC_Carmen_CALP010067260-1 | 1774 | - | - | - | 3 - 1770 | Fragmented element |
| BLAST search | RLC_Carmen_CALP010103792-1 | 1861 | - | - | - | 121 - 1859 | Fragmented element |
| BLAST search | RLC_Carmen_CALP010121574-1 | 692 | - | - | - | 15 - 685 | Fragmented element |
| BLAST search | RLC_Carmen_CALP010163694-1 | 1611 | - | - | - | 2- 1572 | Fragmented element |
| BLAST search | RLC_Carmen_CALP010165878-1 | 881 | - | - | - | 123 - 715 | Fragmented element |
| BLAST search | RLC_Carmen_CALP010224113-1 | 633 | - | - | - | 3 - 617 | Fragmented element |
| BLAST search | RLC_Carmen_CALP010306555-1 | 1673 | - | - | - | 318 - 1672 | Fragmented element |
| BLAST search | RLC_Carmen_CALP010308935-1 | 1666 | - | - | - | 5 - 1375 | Fragmented element |
| BLAST search | RLC_Carmen_CALP010317155-1 | 1651 | .- | - | - | 66 - 788 | Fragmented element |
| BLAST search | RLC_Carmen_CALP010357964-1 | 1580 | - | - | - | 406 -1579 | Fragmented element |
| BLAST search | RLC_Carmen_CALP010446884-1 | 1444 | - | - | - | 7 - 1443 | Fragmented element |
| BLAST search | RLC_Carmen_CALP010464375-1 | 750 | - | - | - | 1 - 583 | Fragmented element |
| BLAST search | RLC_Carmen_CALP010644204-1 | 602 | - | - | - | 19 - 600 | Fragmented element |
| BLAST search | RLC_Carmen_CALP010736972-1 | 851 | - | - | - | 292 - 850 | Fragmented element |
| BLAST search | RLC_Carmen_CALP010889185-1 | 808 | - | - | - | 3 - 798 | Fragmented element |
| BLAST search | RLC_Carmen_CALP011001485-1 | 660 | - | - | - | 109 - 656 | Fragmented element |
| BLAST search | RLC_Carmen_CALP011112138-1 | 968 | - | - | - | 6 - 967 | Fragmented element |
| BLAST search | RLC_Carmen_CALP011191886-1 | 933 | - | - | - | 1 - 908 | Fragmented element |
| BLAST search | RLC_Carmen_CALP011233976-1 | 588 | - | - | - | 34 - 586 | Fragmented element |
| BLAST search | RLC_Carmen_CALP011255795-1 | 907 | - | - | - | 196 - 817 | Fragmented element |
| BLAST search | RLC_Carmen_CALP011468448-1 | 831 | - | - | - | 14 - 811 | Fragmented element |
| BLAST search | RLC_Carmen_CALP011598660-1 | 795 | - | - | - | 1 - 773 | Fragmented element |
| BLAST search | RLC_Carmen_CALP011654708-1 | 599 | - | - | - | 3 - 368 | Fragmented element |
| BLAST search | RLC_Carmen_CALP011659433-1 | 781 | - | - | - | 54 - 494 | Fragmented element |
| BLAST search | RLC_Carmen_CALP011662724-1 | 779 | - | - | - | 103 - 704 | Fragmented element |
| BLAST search | RLC_Carmen_CALP011765918-1 | 754 | - | - | - | 6 - 754 | Fragmented element |
| BLAST search | RLC_Carmen_CALP011828547-1 | 614 | - | - | - | 3 - 421 | Fragmented element |
| BLAST search | RLC_Carmen_CALP012055131-1 | 638 | - | - | - | 2 - 625 | Fragmented element |
| BLAST search | RLC_Carmen_CALP012239291-1 | 647 | - | - | - | 39 -487 | Fragmented element |
| BLAST search | RLC_Carmen_CALP012348547-1 | 625 | - | - | - | 23 - 449 | Fragmented element |
| BLAST search | RLC_Carmen_CALP012501138-1 | 602 | - | - | - | 29 - 597 | Fragmented element |
| BLAST search | RLC_Carmen_CALP012552884-1 | 596 | - | - | - | 15 - 575 | Fragmented element |
| BLAST search | RLC_Carmen_CALP012884601-1 | 544 | - | - | - | no | Fragmented element |
| BLAST search | RLC_Carmen_CALP013294378-1 | 495 | - | - | - | 7 - 490 | Fragmented element |
| BLAST search | RLC_Carmen_CALP013303800-1 | 494 | - | - | - | 4 - 492 | Fragmented element |
| BLAST search | RLC_Carmen_AOCO010608854-1 | 2952 | 1 - 132 | - | - | 299 - 2809 | Partial element, truncated 3´end |
| BLAST search | RLC_Carmen_AOCO010690664-1 | 571 | 1 - 129 |  |  | 278 - 569 | Partial element, truncated 3´end |
| BLAST search | RLC_Carmen_CALP010028816-1 | 2936 | 1 - 123 | - | - | 272 - 2675 | Partial element, truncated 3´end |
| BLAST search | RLC_Carmen_CALP010236408-1 | 985 | 1 - 127 | - | - | 275 - 906 | Partial element, truncated 3´end |
| BLAST search | RLC_Carmen_CALP010332330-1 | 1172 | 24 - 190 |  |  | 335 - 982 | Partial element, truncated 3´end |
| BLAST search | RLC_Carmen_CALP011000205-1 | 910 | 1 - 163 |  |  | 307 - 908 | Partial element, truncated 3´end |
| BLAST search | RLC_Carmen_AOCO010256456-1 | 2568 | - | 2420 - 2568 | - | 002 - 2564 | Partial element, truncated 5´end |
| BLAST search | RLC_Carmen_AOCO010608853-1 | 1957 | - | 1827 - 1957 |  | 77 - 1631 | Partial element, truncated 5´end |
| BLAST search | RLC_Carmen_AOCO010690661-1 | 4574 | - | 4446 - 4574 | - | 1535 - 4308 | Partial element, truncated 5´end |
| BLAST search | RLC_Carmen_CALP010093698-1 | 1793 | - | 1665 - 1793 |  | 602 - 1549 | Partial element, truncated 5´end |
| LTR finder | RLC_Genoveva_JROL01007197 | 5132 | 1 - 215 | 4918 - 5132 | GAGGC | 319 - 4876 | Complete element |
| LTR finder | RLC_Genoveva_AOCO010237191-1 | 5182 | 1 -217 | 4978 - 5182 | TGAAT | 375 - 4936 | Complete element |
| LTR finder | RLC_Genoveva_AOCO010066794-1 | 2956 | 1- 215 | - | - | 319 - 2943 | Partial element, truncated 3´end |
| BLAST search | RLC_Genoveva_AOCO010066793-1 | 1952 | - | 1738 - 1952 | - | 253 - 1696 | Partial element, truncated 5´end |
| BLAST search | RLC_Genoveva_AOCO010233966-1 | 4538 | 1 -205 | - | - | 361 - 4538 | Partial element, truncated 3´end |
| BLAST search | RLC_Genoveva_CALP010001044-1 | 3910 | 1 -187 | - | - | 344 - 3907 | Partial element, truncated 3´end |
| LTR-FINDER | RLG_Francisca_JROL01008273 | 5279 | 02- 321 | 4960 - 5279 | CTGTC | 497- 4962 | Complete element |
| LTR-FINDER | RLG_Francisca_AOCO010454749-1 | 5278 | 01 - 320 | 4960 - 5279 | CTGTC | 496 - 4961 | Complete element |
| LTR-FINDER | RLG_Francisca_AOCO010144477-1 | 5065 | 04 - 320 | 4750 - 5065 | CAAAT | 844 - 4783 | Complete element |
| BLAST search | RLG_Francisca_CALP010003649-1 | 4700 | 01 - 320 |  |  | 850 - 4700 | Partial element, truncated 3´end |
| BLAST search | RLG_Francisca_CALP010061300-1 | 2745 | - | - |  | 02 - 1743 | Fragmented element |
| BLAST search | RLG_Francisca_CALP010153829-1 | 2109 | - | - |  | 61 - 2109 | Fragmented element |
| BLAST search | RLG_Francisca_CALP010090108-1 | 1912 | - | - |  | 221 - 1910 | Fragmented element |
| BLAST search | RLG_Francisca_CALP010296358-1 | 1688 | - | - |  | 03 - 1684 | Fragmented element |
| BLAST search | RLG_Francisca_AEOM01028590-1 | 1560 | 01 - 322 | - |  | 435 - 1553 | Partial element, truncated 3´end |
| BLAST search | RLG_Francisca_CALP010313935-1 | 1526 | 01 - 320 | - |  | 901 - 1503 | Partial element, truncated 3´end |
| BLAST search | RLG_Francisca_CALP010289309-1 | 1474 | 01 - 319 | - |  | 720 - 1448 | Partial element, truncated 3´end |
| BLAST search | RLG_Francisca_CALP010450630-1 | 1447 | - | - |  | 22 -1208 | Fragmented element |
| BLAST search | RLG_Francisca_AEOM01048149-1 | 1391 | - | - |  | 01 - 1391 | Fragmented element |
| BLAST search | RLG_Francisca_CALP010532755-1 | 1352 | - | - |  | 24 - 1350 | Fragmented element |
| BLAST search | RLG_Francisca_CALP010594168-1 | 1290 | - | - |  | 03 - 1290 | Fragmented element |
| BLAST search | RLG_Francisca_CALP010619063-1 | 1266 | 02 - 297 | - |  | 824 - 1263 | Partial element, truncated 3´end |
| BLAST search | RLG_Francisca_CALP010691093-1 | 1207 | 001 - 57 | - |  | 585 - 1008 | Partial element, truncated 3´end |
| BLAST search | RLG_Francisca_CALP010903443-1 | 1069 | - | - |  | 03 - 1046 | Fragmented element |
| BLAST search | RLG_Francisca_CALP010635319-1 | 830 | - | 513 - 829 |  | 04 - 636 | Partial element, truncated 5´end |
| BLAST search | RLG_Francisca_CALP011534613-1 | 814 | - | - |  | 27 - 813 | Fragmented element |
| BLAST search | RLG_Francisca_CALP010535643-1 | 800 | - | 484 - 800 |  | 03 - 609 | Partial element, truncated 5´end |
| BLAST search | RLG_Francisca_CALP011637439-1 | 785 | - | - |  | 51 - 761 | Fragmented element |
| BLAST search | RLG_Francisca_CALP011637278-1 | 783 | - | - |  | 57 - 731 | Fragmented element |
| BLAST search | RLG_Francisca_CALP011856515-1 | 729 | - | - |  | 29 - 724 | Fragmented element |
| BLAST search | RLG_Francisca_CALP012053594-1 | 688 | - | - |  | 398 - 664 | Fragmented element |
| BLAST search | RLG_Francisca_CALP012062839-1 | 682 | - | - |  | 29 - 662 | Fragmented element |
| BLAST search | RLG_Francisca_AEOM01071161-1 | 595 | - | - |  | 10 - 588 | Fragmented element |
| BLAST search | RLG_Francisca_CALP012632193-1 | 580 | - | - |  | 02 - 554 | Fragmented element |
| BLAST search | RLG_Francisca_CALP010869653-1 | 537 | - | - |  | 02 - 446 | Fragmented element |
| BLAST search | RLG_Francisca_CALP013495848-1 | 467 | - | - |  | 03 - 358 | Fragmented element |
| BLAST search | RLG_Francisca_AEOM01094980-1 | 456 | - | - |  | 02 - 454 | Fragmented element |
| BLAST search | RLG_Francisca_CALP013648311-1 | 448 | - | - |  | 03 - 338 | Fragmented element |
| BLAST search | RLG_Francisca_CALP013685092-1 | 445 | - | - |  | 89 - 405 | Fragmented element |
| BLAST search | RLG_Francisca_CALP013813545-1 | 418 | - | - |  | 02 - 418 | Fragmented element |
| LTR STRUC | RLX_Gabrielle_JROL01007833 | 2644 | 1 - 685 | 1965- 2644 | ACATT | 776 - 2151 | Complete element |
| LTR STRUC | RLX_Gabrielle_AOCO010200744-1 | 2644 | 1 - 685 | 1965- 2644 | ACATT | 776 - 2151 | Fragmented element |
| BLAST search | RLX_Gabrielle_CALP010466105-1 | 1419 | 1 -308 | - | - | 496 - 1404 | Fragmented element |
| BLAST search | RLX_Gabrielle_CALP010124160-1 | 2098 | 1 - 464 | 1638 - 2097 | - | 776 - 2151 | Complete element |
| BLAST search | RLX_Gabrielle_CALP010723531-1 | 1185 | - | - | - | 7 - 326 | Fragmented element |
| BLAST search | RLX_Gabrielle_CALP013409816-1 | 483 | - | - | - | 78 - 356 | Fragmented element |
| BLAST search | RLX_Gabrielle_CALP012559367-1 | 596 | - | - | - | 3 - 419 | Fragmented element |
| BLAST search | RLX_Gabrielle_CALP013361237-1 | 488 | - | - | - | 10 - 411 | Fragmented element |
| BLAST search | RLX_Gabrielle_CALP010625057-1 | 640 | 1 - 640 | - | - | - | Partial element, truncated 3´end |
| BLAST search | RLX_Gabrielle_CALP010054491-1 | 725 | - | - | - | 310 - 534 | Fragmented element |
| BLAST search | RLX_Gabrielle_CALP010532956-1 | 904 | - | - | - | 3 - 224 | Fragmented element |
| LTR_FINDER | RLX_Victoria_AOCO010202150-1 | 8698 | 001 - 2527 | 6188 - 8698 | GTTT | 490 - 7652 | Complete element, partial coding capacity |
| LTR_FINDER | RLX_Victoria_AOCO010079434-1 | 3250 | 01- 951 | 2295 - 3246 | AGAGAG | 1067 - 2340 | Complete element, partial coding capacity |
| LTR_FINDER | RLX_Victoria_AOCO010565844-1 | 3147 | 01 - 997 | 2110 - 3147 | CTTGT | 1094 - 2026 | Complete element, partial coding capacity |
| LTR_FINDER | RLX_Victoria_AOCO010269025-1 | 3011 | 01 - 895 | 2124 - 3011 | CGCTA | 1118 - 2039 | Complete element, partial coding capacity |
| LTR_FINDER | RLX_Victoria_AOCO010584881-1 | 3106 | 01 - 1003 | 2104 - 3106 | TCACA | 1115 - 2317 | Complete element, partial coding capacity |
| LTR_FINDER | RLX_Victoria_AOCO010373096-1 | 3096 | 03 - 991 | 2105 - 3096 | GAGAC | 1114 - 2016 | Complete element, partial coding capacity |
| LTR_FINDER | RLX_Victoria_AOCO010374238-1 | 3103 | 003 - 991 | 2105 - 3096 | TAGGA | 1290 - 2094 | Complete element, partial coding capacity |
| LTR_FINDER | RLX_Victoria_AOCO010112050-1 | 3078 | 01 - 975 | 2098 - 3072 | TAGGA | 1103 - 2019 | Complete element, partial coding capacity |
| LTR_FINDER | RLX_Victoria_AOCO010703147-1 | 3068 | 01 - 978 | 2091 - 3068 | AAACG | 1098 - 2002 | Complete element, partial coding capacity |
| LTR_FINDER | RLX_Victoria_AOCO010103576-1 | 3062 | 01 -978 | 2085 - 3062 | CGCGG | 1098 - 2002 | Complete element, partial coding capacity |
| LTR_FINDER | RLX_Victoria_AOCO010325327-1 | 3046 | 01 - 967 | 2079 - 3046 | AGAGG | 1087 - 1995 | Complete element, partial coding capacity |
| LTR_FINDER | RLX_Victoria_AOCO010428135-1 | 3030 | 03 - 970 | 2061 - 3030 | CACCT | 1247 - 2158 | Complete element, partial coding capacity |
| LTR_FINDER | RLX_Victoria_AOCO010465341-1 | 3021 | 01 - 955 | 2068 - 3021 | TGGCC | 1076 - 2008 | Complete element, partial coding capacity |
| LTR_FINDER | RLX_Victoria_AOCO010317797-1 | 3009 | 01 - 940 | 2070 - 3009 | - | 1066 - 1972 | Complete element, partial coding capacity |
| LTR_FINDER | RLX_Victoria_AOCO010785725-1 | 2989 | 01 - 938 | 2053 - 2989 | AGAGA | 1058 - 1529 | Complete element, partial coding capacity |
| LTR_FINDER | RLX_Victoria_AOCO010667853-1 | 2898 | 01 - 884 | 2016 - 2898 | CCCGC | 1004 - 1909 | Complete element, partial coding capacity |
| LTR_FINDER | RLX_Victoria_AOCO010536538-1 | 2896 | 01 - 906 | 2001 - 2896 | CCACC | 1105 - 1914 | Complete element, partial coding capacity |
| LTR_FINDER | RLX_Victoria_AOCO010132854-1 | 2895 | 01 - 890 | 2002 - 2895 | GGTGA | 1010 - 1921 | Complete element, partial coding capacity |
| LTR_FINDER | RLX_Victoria_AOCO010035872-1 | 2829 | 01- 860 | 1970 - 2819 | AAAA | 987 - 1886 | Complete element, partial coding capacity |
| LTR_FINDER | RLX_Victoria_AOCO010189842-1 | 2816 | 01 - 874 | 1946 - 2816 | AAGGT | 994 - 1862 | Complete element, partial coding capacity |
| LTR_FINDER | RLX_Victoria_CALP010004242-1 | 2802 | 01 - 849 | 1955 - 2802 | GGGGC | 977 -1962 | Complete element, partial coding capacity |
| LTR_FINDER | RLX_Victoria_AOCO010154687-1 | 2787 | 01 - 848 | 1931 - 2787 | ACTTA | 973 - 1847 | Complete element, partial coding capacity |
| LTR_FINDER | RLX_Victoria_CALP010032496-1 | 2775 | 01 - 761 | 2008 - 2764 | - | 1026 - 1933 | Complete element, partial coding capacity |
| LTR_FINDER | RLX_Victoria_CALP010055710-1 | 2682 | 01 - 588 | 2096 - 2682 | - | 1618 - 2009 | Complete element, partial coding capacity |
| LTR_FINDER | RLX_Victoria_AOCO010281794-1 | 2671 | 01 - 807 | 1871 - 2671 | AGTGC | 937 - 1886 | Complete element, partial coding capacity |
| LTR_FINDER | RLX_Victoria_AOCO010261338-1 | 2633 | 23 -616 | 2035 - 2631 | GTTT | 1043 - 1948 | Complete element, partial coding capacity |
| LTR_FINDER | RLX_Victoria_AOCO010600519-1 | 2448 | 01 -671 | 1777 - 2448 | - | 816 - 1680 | Complete element, partial coding capacity |
| LTR_FINDER | RLX_Victoria_AOCO010632979-1 | 2366 | 01 - 219 | 2128 - 2346 | - | 339 - 1247 | Complete element, partial coding capacity |
| LTR_FINDER | RLX_Victoria_CALP010071114-1 | 2184 | 01 - 215 | 1956 - 2169 | - | 643 - 1539 | Complete element, partial coding capacity |
| LTR_FINDER | RLX_Victoria_CALP010145898-1 | 2087 | 01 - 122 | 1968 - 2087 | - | 347 - 1245 | Complete element, partial coding capacity |
| LTR_FINDER | RLX_Victoria_AOCO010100749-1 | 1939 | 01- 419 | 1521 - 1939 | ACAAG | 529 - 1437 | Complete element, partial coding capacity |
| LTR_FINDER | RLX_Victoria_ JROL01006440 | 1895 | 01 - 372 | 1524 - 1895 | - | 419 - 1396 | Complete element, partial coding capacity |
| BLAST | RLX_Victoria_AOCO010278508-1 | 3145 | 01 - 1048 | 2095 - 3142 | - | 177 - 2717 | Complete element, partial coding capacity |
| BLAST | RLX_Victoria_AOCO010372201-1 | 2481 | 01 - 463 | 2019 - 2481 | - | 1032 - 1901 | Complete element, partial coding capacity |
| BLAST | RLX_Victoria_CALP010064643-1 | 2448 | - | - | - | 1122 - 2027 | Fragmented element |
| LTR-FINDER | RLC_Facunda_ JROL01000922 | 4842 | 1 - 138 | 4704 -4842 | - | 2388 - 4673 | Complete element |
| LTR-FINDER | RLC_Facunda_AOCO010288406-1 | 1552 | - | - | - | 001 -1551 | Fragmented element |
| BLAST search | RLC_Facunda_AOCO010051481-1 | 1914 | 1 -138 | 1776 - 1914 | - | 238 -1719 | Complete element |
| BLAST search | RLC_Facunda_AOCO010674073-1 | 1383 | 01- 167 | 1218 - 1383 | - | 287 - 1069 | Complete element |
| BLAST search | RLC_Facunda_AOCO010338758-1 | 3391 | - | - | - | 64 - 3339 | Fragmented element |
| BLAST search | RLC_Facunda_CALP010045365-1 | 2971 | - | - | - | 341 - 2923 | Fragmented element |
| BLAST search | RLC_Facunda_CALP010039896-1 | 2528 | - | - | - | 298 - 2515 | Fragmented element |
| BLAST search | RLC_Facunda_AOCO010700758-1 | 2460 | - | - | - | 16 - 2090 | Fragmented element |
| BLAST search | RLC_Facunda_AOCO010700757-1 | 2456 | - | - | - | 456 - 2198 | Fragmented element |
| BLAST search | RLC_Facunda_AOCO010021163-1 | 2390 | - | - | - | 003 - 2389 | Fragmented element |
| BLAST search | RLC_Facunda_CALP010148002-1 | 2128 | - | - | - | 009 - 2128 | Fragmented element |
| BLAST search | RLC_Facunda_AOCO010173361-1 | 2081 | - | - | - | 002 - 2079 | Fragmented element |
| BLAST search | RLC_Facunda_CALP010167398-1 | 2055 | - | - | - | 667 - 1993 | Fragmented element |
| BLAST search | RLC_Facunda_AOCO010631395-1 | 1945 | - | - | - | 281 - 1900 | Fragmented element |
| BLAST search | RLC_Facunda_AOCO010590568-1 | 1930 | - | - | - | 73 - 1392 | Fragmented element |
| BLAST search | RLC_Facunda_AOCO010421870-1 | 1924 | - | - | - | 160 - 1785 | Fragmented element |
| BLAST search | RLC_Facunda_AEOM01238425-1 | 1784 | - | - | - | 160 - 1743 | Fragmented element |
| BLAST search | RLC_Facunda_AOCO010043189-1 | 1781 | - | - | - | 007 - 1610 | Fragmented element |
| BLAST search | RLC_Facunda_AOCO010707907-1 | 1775 | - | - | - | 10 - 1644 | Fragmented element |
| BLAST search | RLC_Facunda_AOCO010176863-1 | 1746 | - | - | - | 329 - 1501 | Fragmented element |
| BLAST search | RLC_Facunda_AOCO010412837-1 | 1696 | - | - | - | 31 - 1649 | Fragmented element |
| BLAST search | RLC_Facunda_AOCO010603788-1 | 1696 | - | - | - | 126 - 1347 | Fragmented element |
| BLAST search | RLC_Facunda_CALP010296744-1 | 1679 | - | - | - | 68 -954 | Fragmented element |
| BLAST search | RLC_Facunda_AOCO010191627-1 | 1501 | - | - | - | 76 - 1395 | Fragmented element |
| BLAST search | RLC_Facunda_CALP010342614-1 | 1474 | - | - | - | 707 - 1466 | Fragmented element |
| BLAST search | RLC_Facunda_CALP010431080-1 | 1466 | - | - | - | 30 - 1466 | Fragmented element |
| BLAST search | RLC_Facunda_CALP010435951-1 | 1461 | - | - | - | 38 - 1460 | Fragmented element |
| BLAST search | RLC_Facunda_AOCO010636304-1 | 1454 | - | - | - | 36 - 1346 | Fragmented element |
| BLAST search | RLC_Facunda_AOCO010716822-1 | 1447 | - | - | - | 24 - 1447 | Fragmented element |
| BLAST search | RLC_Facunda_AOCO010653612-1 | 1436 | - | - | - | 76 - 1419 | Fragmented element |
| BLAST search | RLC_Facunda_AOCO010636834-1 | 1408 | - | - | - | 123 - 1210 | Fragmented element |
| BLAST search | RLC_Facunda_CALP010480293-1 | 1407 | - | - | - | 443 - 1406 | Fragmented element |
| BLAST search | RLC_Facunda_AOCO010317688-1 | 1404 | - | - | - | 352 - 1302 | Fragmented element |
| BLAST search | RLC_Facunda_AOCO010778529-1 | 1368 | - | - | - | 137 - 1321 | Fragmented element |
| BLAST search | RLC_Facunda_CALP010527190-1 | 1358 | - | - | - | 413 - 1357 | Fragmented element |
| BLAST search | RLC_Facunda_AOCO010498211-1 | 1343 | - | - | - | 003 - 1204 | Fragmented element |
| BLAST search | RLC_Facunda_AOCO010239865-1 | 1317 | - | - | - | 403 - 1317 | Fragmented element |
| BLAST search | RLC_Facunda_AOCO010491830-1 | 1908 | - | - | - | 001 - 1908 | Fragmented element |
| BLAST search | RLC_Facunda_AOCO010021572-1 | 1297 | - | - | - | 481 - 1296 | Fragmented element |
| BLAST search | RLC_Facunda_AOCO010339256-1 | 1270 | - | - | - | 57 - 1268 | Fragmented element |
| BLAST search | RLC_Facunda_AOCO010105934-1 | 1268 | - | - | - | 503 - 1267 | Fragmented element |
| BLAST search | RLC_Facunda_AOCO010503571-1 | 1254 | - | - | - | 185 - 1246 | Fragmented element |
| BLAST search | RLC_Facunda_CALP010660128-1 | 1231 | - | - | - | 31 - 1229 | Fragmented element |
| BLAST search | RLC_Facunda_AOCO010664168-1 | 1202 | - | - | - | 136 - 1200 | Fragmented element |
| BLAST search | RLC_Facunda_AOCO010325142-1 | 1193 | - | - | - | 02 - 1051 | Fragmented element |
| BLAST search | RLC_Facunda_AOCO010438938-1 | 1183 | - | - | - | 02 - 1097 | Fragmented element |
| BLAST search | RLC_Facunda_AOCO010552457-1 | 1176 | - | - | - | 107 - 1168 | Fragmented element |
| BLAST search | RLC_Facunda_AOCO010215994-1 | 1155 | - | - | - | 492 - 1154 | Fragmented element |
| BLAST search | RLC_Facunda_AOCO010392677-1 | 1148 | - | - | - | 76 - 1146 | Fragmented element |
| BLAST search | RLC_Facunda_AOCO010379024-1 | 1147 | - | - | - | 64 - 1140 | Fragmented element |
| BLAST search | RLC_Facunda_CALP010775656-1 | 1146 | - | - | - | 04 - 543 | Fragmented element |
| BLAST search | RLC_Facunda_CALP010778523-1 | 1145 | - | - | - | 264 - 1143 | Fragmented element |
| BLAST search | RLC_Facunda_AOCO010416663-1 | 1142 | - | - | - | 541 - 1040 | Fragmented element |
| BLAST search | RLC_Facunda_CALP010781314-1 | 1140 | - | - | - | 01 - 963 | Fragmented element |
| BLAST search | RLC_Facunda_AOCO010799006-1 | 1137 | - | - | - | 21 - 1229 | Fragmented element |
| BLAST search | RLC_Facunda_CALP010833900-1 | 1110 | - | - | - | 323 - 1096 | Fragmented element |
| BLAST search | RLC_Facunda_AOCO010665250-1 | 1106 | - | - | - | 229 - 1035 | Fragmented element |
| BLAST search | RLC_Facunda_AOCO010609284-1 | 1095 | - | - | - | 104 - 1094 | Fragmented element |
| BLAST search | RLC_Facunda_CALP010862251-1 | 1095 | - | - | - | 03 - 1095 | Fragmented element |
| BLAST search | RLC_Facunda_AOCO010285134-1 | 1087 | - | - | - | 339 - 1085 | Fragmented element |
| BLAST search | RLC_Facunda_CALP010877442-1 | 1083 | - | - | - | 120 - 1082 | Fragmented element |
| BLAST search | RLC_Facunda_CALP010881281-1 | 1082 | - | - | - | 02 - 1081 | Fragmented element |
| BLAST search | RLC_Facunda_AOCO010558470-1 | 1073 | - | - | - | 100 - 1073 | Fragmented element |
| BLAST search | RLC_Facunda_AOCO010121158-1 | 1049 | - | - | - | 361 - 1047 | Fragmented element |
| BLAST search | RLC_Facunda_AOCO010864957-1 | 1046 | - | - | - | 04 - 945 | Fragmented element |
| BLAST search | RLC_Facunda_CALP010657372-1 | 1045 | - | - | - | 552 -1045 | Fragmented element |
| BLAST search | RLC_Facunda_CALP010951044-1 | 1037 | - | - | - | 26 - 817 | Fragmented element |
| BLAST search | RLC_Facunda_AOCO010803562-1 | 1031 | - | - | - | 03 - 995 | Fragmented element |
| BLAST search | RLC_Facunda_CALP010968694-1 | 1031 | - | - | - | 07 - 1030 | Fragmented element |
| BLAST search | RLC_Facunda_CALP010981426-1 | 1027 | - | - | - | 03 - 1027 | Fragmented element |
| BLAST search | RLC_Facunda_AOCO010315091-1 | 1010 | - | - | - | 544 - 1008 | Fragmented element |
| BLAST search | RLC_Facunda_AEOM01280901-1 | 1009 | - | - | - | 01 - 860 | Fragmented element |
| BLAST search | RLC_Facunda_CALP011003546-1 | 1008 | - | - | - | 498 - 968 | Fragmented element |
| BLAST search | RLC_Facunda_AOCO010055959-1 | 987 | - | - | - | 04 - 984 | Fragmented element |
| BLAST search | RLC_Facunda_AOCO010483387-1 | 950 | - | - | - | 03 - 941 | Fragmented element |
| BLAST search | RLC_Facunda_AOCO010392678-1 | 939 | - | - | - | 124 - 938 | Fragmented element |
| BLAST search | RLC_Facunda_CALP011206695-1 | 926 | - | - | - | 119 - 924 | Fragmented element |
| BLAST search | RLC_Facunda_CALP011457748-1 | 836 | - | - | - | 03 - 834 | Fragmented element |
| BLAST search | RLC_Facunda_CALP010253422-1 | 823 | - | - | - | 03 -688 | Fragmented element |
| BLAST search | RLC_Facunda_CALP011540241-1 | 808 | - | - | - | 03 - 808 | Fragmented element |
| BLAST search | RLC_Facunda_CALP011623892-1 | 785 | - | - | - | 47 - 716 | Fragmented element |
| BLAST search | RLC_Facunda_AOCO010007830-1 | 1526 | - | - | - | 66 - 1381 | Fragmented element |
| BLAST search | RLC_Facunda_AOCO010025613-1 | 1405 | - | - | - | 79 - 1267 | Fragmented element |
| BLAST search | RLC_Facunda_AOCO010570347-1 | 1384 | - | - | - | 002 - 1243 | Fragmented element |
| BLAST search | RLC_Facunda_AOCO010292560-1 | 1210 | - | - | - | 394 - 1209 | Fragmented element |
| BLAST search | RLC_Facunda_CALP011356663-1 | 872 | - | - | - | 108 - 863 | Fragmented element |
| BLAST search | RLC_Facunda_AOCO010056355-1 | 853 | - | - | - | 02 - 853 | Fragmented element |
| BLAST search | RLC_Facunda_AOCO010841206-1 | 1482 | 001 - 257 | - | - | 280 - 1480 | Partial element, truncated 3´end |
| BLAST search | RLC_Facunda_AOCO010483471-1 | 1254 | - | 1116 - 1254 | - | 3 -1515 | Partial element, truncated 5´end |
| BLAST search | RLC_Facunda_AOCO010483637-1 | 2429 | - | 2291 - 2429 | - | 409 - 2290 | Partial element, truncated 5´end |
| BLAST search | RLC_Facunda_AOCO010639276-1 | 2226 | - | 2105 -2226 | - | 002 - 2104 | Partial element, truncated 5´end |
| BLAST search | RLC_Facunda_AOCO010507906-1 | 2078 | - | 1940 - 2078 | - | 272 - 1937 | Partial element, truncated 5´end |
| BLAST search | RLC_Facunda_AOCO010438937-1 | 1958 | - | 1820 - 1958 | - | 215 - 1919 | Partial element, truncated 5´end |
| BLAST search | RLC_Facunda_CALP010205893-1 | 1914 | - | 1786 - 1924 | - | 1- 1886 | Partial element, truncated 5´end |
| BLAST search | RLC_Facunda_AOCO010239426-1 | 1908 | - | 1770 - 1908 | - | 140 - 1739 | Partial element, truncated 5´end |
| BLAST search | RLC_Facunda_AOCO010011508-1 | 1525 | - | 1387 - 1525 | - | 14 -1386 | Partial element, truncated 5´end |
| BLAST search | RLC_Facunda_AOCO010821728-1 | 1345 | - | 1208 -1345 | - | 003 -1205 | Partial element, truncated 5´end |
| BLAST search | RLC_Facunda_AOCO010937765-1 | 1258 | - | 1120 - 1258 | - | 004 -1119 | Partial element, truncated 5´end |
| BLAST search | RLC_Facunda_AOCO010323995-1 | 3075 | - | 2938 - 3075 | - | 16 -2887 | Partial element, truncated 5´end |
| BLAST search | RLC_Facunda_AOCO010672136-1 | 1917 | - | 1779 - 1917 | - | 151 - 1796 | Partial element, truncated 5´end |
| BLAST search | RLC_Facunda_AOCO010665249-1 | 1890 | - | 1752 - 1890 | - | 127 - 1751 | Partial element, truncated 5´end |
| BLAST search | RLC_Facunda_AOCO010717991-1 | 1888 | - | 1750 - 1888 | - | 127 - 1749 | Partial element, truncated 5´end |
| BLAST search | RLC_Facunda_AOCO010781008-1 | 1877 | - | 1748 - 1886 | - | 118 - 1723 | Partial element, truncated 5´end |
| BLAST search | RLC_Facunda_AOCO010027701-1 | 1865 | - | 1727 - 1865 | - | 100 - 1725 | Partial element, truncated 5´end |
| BLAST search | RLC_Facunda_AOCO010054899-1 | 1863 | - | 1725 - 1863 | - | 99 - 1724 | Partial element, truncated 5´end |
| BLAST search | RLC_Facunda_AOCO010037719-1 | 1852 | - | 1715 - 1852 | - | 89 - 117 | Partial element, truncated 5´end |
| BLAST search | RLC_Facunda_AOCO010060755-1 | 1839 | - | 1700 - 1839 | - | 73 - 1699 | Partial element, truncated 5´end |
| BLAST search | RLC_Facunda_AOCO010308360-1 | 1836 | - | 1697 - 1836 | - | 64 - 1695 | Partial element, truncated 5´end |
| BLAST search | RLC_Facunda_AOCO010032711-1 | 1834 | - | 1696 - 1834 | - | 70 - 1695 | Partial element, truncated 5´end |
| BLAST search | RLC_Facunda_AOCO010060912-1 | 1824 | - | 1687 - 1824 | - | 58 - 1648 | Partial element, truncated 5´end |
| BLAST search | RLC_Facunda_AOCO010395056-1 | 1811 | - | 1680 - 1811 | - | 60 -1879 | Partial element, truncated 5´end |
| BLAST search | RLC_Facunda_AOCO010684240-1 | 1793 | - | 1655 - 1793 | - | 32 - 1654 | Partial element, truncated 5´end |
| BLAST search | RLC_Facunda_AOCO010616852-1 | 1783 | - | 1645 - 1783 | - | 21 - 1644 | Partial element, truncated 5´end |
| BLAST search | RLC_Facunda_AOCO010649532-1 | 1779 | - | 1641 - 1779 | - | 21 - 1638 | Partial element, truncated 5´end |
| BLAST search | RLC_Facunda_AOCO010111008-1 | 1772 | - | 1666 - 1751 | - | 40 - 1665 | Partial element, truncated 5´end |
| BLAST search | RLC_Facunda_AOCO010587922-1 | 1742 | - | 1604 - 1742 | - | 003 - 1603 | Partial element, truncated 5´end |
| BLAST search | RLC_Facunda_AOCO010629011-1 | 1687 | - | 1549 - 1687 | - | 003 - 1518 | Partial element, truncated 5´end |
| BLAST search | RLC_Facunda_AOCO010249574-1 | 1632 | - | 1494 - 1632 | - | 009 - 1493 | Partial element, truncated 5´end |
| BLAST search | RLC_Facunda_AOCO010610278-1 | 1534 | - | 1496 - 1534 | - | 60 - 1945 | Partial element, truncated 5´end |
| BLAST search | RLC_Facunda_AOCO010068876-1 | 1529 | - | 1496 - 1529 | - | 2 - 1390 | Partial element, truncated 5´end |
| BLAST search | RLC_Facunda_AOCO010292561-1 | 1489 | - | 1351 - 1489 | - | 1 - 1350 | Partial element, truncated 5´end |
| BLAST search | RLC_Facunda_AOCO010021571-1 | 1459 | - | 1321 - 1459 | - | 1 -1320 | Partial element, truncated 5´end |
| BLAST search | RLC_Facunda_AOCO010582471-1 | 1450 | - | 1312 - 1450 | - | 002 - 1311 | Partial element, truncated 5´end |
| BLAST search | RLC_Facunda_AOCO010590567-1 | 1344 | - | 1205 - 1343 | - | 001 - 1206 | Partial element, truncated 5´end |
| BLAST search | RLC_Facunda_AOCO010508171-1 | 1318 | - | 1181 - 1318 | - | 003 - 1180 | Partial element, truncated 5´end |
| BLAST search | RLC_Facunda_AOCO010692732-1 | 1312 | - | 1174 - 1312 | - | 003 - 1173 | Partial element, truncated 5´end |
| BLAST search | RLC_Facunda_CALP010166116-1 | 1304 | - | 1168 - 1304 | - | 001 - 1167 | Partial element, truncated 5´end |
| BLAST search | RLC_Facunda_AOCO010237062-1 | 1300 | - | 1158 - 1300 | - | 001 - 1155 | Partial element, truncated 5´end |
| BLAST search | RLC_Facunda_AOCO010545762-1 | 1263 | - | 1126 - 1263 | - | 004- 1125 | Partial element, truncated 5´end |
| BLAST search | RLC_Facunda_AOCO010688677-1 | 1258 | - | 1120 - 1258 | - | 004 - 1119 | Partial element, truncated 5´end |
| BLAST search | RLC_Facunda_AOCO010134283-1 | 1257 | - | 1119 - 1257 | - | 004 - 1116 | Partial element, truncated 5´end |
| BLAST search | RLC_Facunda_AOCO010282311-1 | 1236 | - | 1098 - 1236 | - | 002 - 1078 | Partial element, truncated 5´end |
| BLAST search | RLC_Facunda_AOCO010632460-1 | 1234 | - | 1096 - 1234 | - | 001 - 1095 | Partial element, truncated 5´end |
| BLAST search | RLC_Facunda_AOCO010283993-1 | 1220 | - | 1082 - 1220 | - | 02 - 1081 | Partial element, truncated 5´end |
| BLAST search | RLC_Facunda_AOCO010916949-1 | 1216 | - | 1075 - 1213 | - | 01 -1074 | Partial element, truncated 5´end |
| BLAST search | RLC_Facunda_AOCO010803563-1 | 1215 | - | 1074 - 1215 | - | 03 - 1073 | Partial element, truncated 5´end |
| BLAST search | RLC_Facunda_AOCO010579688-1 | 1213 | - | 1075 - 1213 | - | 01 - 1074 | Partial element, truncated 5´end |
| BLAST search | RLC_Facunda_AOCO010201733-1 | 1212 | - | 1074 - 1212 | - | 01 - 1071 | Partial element, truncated 5´end |
| BLAST search | RLC_Facunda_AOCO010120669-1 | 1211 | - | 1072 - 1211 | - | 03 - 1071 | Partial element, truncated 5´end |
| BLAST search | RLC_Facunda_AOCO010317687-1 | 1209 | - | 1081 - 1209 | - | 01 - 1080 | Partial element, truncated 5´end |
| BLAST search | RLC_Facunda_AOCO010381216-1 | 1200 | - | 1082 - 1220 | - | 02 - 1081 | Partial element, truncated 5´end |
| BLAST search | RLC_Facunda_CALP010689395-1 | 1197 | - | 1000 -1183 | - | 167 - 943 | Partial element, truncated 5´end |
| BLAST search | RLC_Facunda_AOCO010668208-1 | 1166 | - | 1030 - 1166 | - | 02 - 1029 | Partial element, truncated 5´end |
| BLAST search | RLC_Facunda_AOCO010788709-1 | 1162 | - | 924 - 1062 | - | 02 - 984 | Partial element, truncated 5´end |
| BLAST search | RLC_Facunda_AOCO010228532-1 | 1131 | - | 992 - 1131 | - | 11 - 991 | Partial element, truncated 5´end |
| BLAST search | RLC_Facunda_AOCO010237773-1 | 1130 | - | 992 - 1130 | - | 11- 991 | Partial element, truncated 5´end |
| BLAST search | RLC_Facunda_AOCO010239784-1 | 1130 | - | 992 - 1130 | - | 11 - 991 | Partial element, truncated 5´end |
| BLAST search | RLC_Facunda_AOCO010284354-1 | 1124 | - | 983 - 1124 | - | 02 - 982 | Partial element, truncated 5´end |
| BLAST search | RLC_Facunda_AOCO010145279-1 | 1121 | - | 983 - 1121 | - | 02- 982 | Partial element, truncated 5´end |
| BLAST search | RLC_Facunda_AOCO010175032-1 | 1121 | - | 983 - 1121 | - | 02- 982 | Partial element, truncated 5´end |
| BLAST search | RLC_Facunda_AOCO010481068-1 | 1121 | - | 983 - 1121 | - | 02- 982 | Partial element, truncated 5´end |
| BLAST search | RLC_Facunda_AOCO010622941-1 | 1121 | - | 983 - 1121 | - | 12 - 982 | Partial element, truncated 5´end |
| BLAST search | RLC_Facunda_AOCO010142555-1 | 1118 | - | 983 - 1118 | - | 02- 982 | Partial element, truncated 5´end |
| BLAST search | RLC_Facunda_AOCO010672444-1 | 1118 | - | 983 - 1118 | - | 02- 982 | Partial element, truncated 5´end |
| BLAST search | RLC_Facunda_AOCO010340314-1 | 1117 | - | 979 - 1117 | - | 04 - 978 | Partial element, truncated 5´end |
| BLAST search | RLC_Facunda_AOCO010205433-1 | 1108 | - | 966 - 1104 | - | 03 - 965 | Partial element, truncated 5´end |
| BLAST search | RLC_Facunda_AOCO010296411-1 | 1104 | - | 966 - 1104 | - | 03 - 965 | Partial element, truncated 5´end |
| BLAST search | RLC_Facunda_AOCO010430070-1 | 1104 | - | 966 - 1104 | - | 03 - 965 | Partial element, truncated 5´end |
| BLAST search | RLC_Facunda_AOCO010196662-1 | 1073 | - | 935 - 1073 | - | 01 - 934 | Partial element, truncated 5´end |
| BLAST search | RLC_Facunda_CALP010889023-1 | 1072 | - | 964 - 1072 | - | 01 - 840 | Partial element, truncated 5´end |
| BLAST search | RLC_Facunda_AOCO010184525-1 | 1068 | - | 930 - 1068 | - | 02 - 929 | Partial element, truncated 5´end |
| BLAST search | RLC_Facunda_AOCO010864958-1 | 1055 | - | 918 - 1055 | - | 06 - 917 | Partial element, truncated 5´end |
| BLAST search | RLC_Facunda_AOCO010732959-1 | 1050 | - | 918 - 1050 | - | 06 - 917 | Partial element, truncated 5´end |
| BLAST search | RLC_Facunda_AOCO010325070-1 | 1048 | - | 915 - 1048 | - | 03 - 914 | Partial element, truncated 5´end |
| BLAST search | RLC_Facunda_AOCO010320908-1 | 1044 | - | 905 - 1043 | - | 02 - 899 | Partial element, truncated 5´end |
| BLAST search | RLC_Facunda_AOCO010471507-1 | 1040 | - | 902 - 1040 | - | 32 - 823 | Partial element, truncated 5´end |
| BLAST search | RLC_Facunda_AOCO010531973-1 | 979 | - | 835 - 973 | - | 04 - 834 | Partial element, truncated 5´end |
| BLAST search | RLC_Facunda_AOCO010278615-1 | 974 | - | 835 - 973 | - | 02 - 834 | Partial element, truncated 5´end |
| BLAST search | RLC_Facunda_AOCO010364409-1 | 971 | - | 833 - 971 | - | 03 - 832 | Partial element, truncated 5´end |
| BLAST search | RLC_Facunda_AOCO010148031-1 | 968 | - | 826 - 968 | - | 02 - 825 | Partial element, truncated 5´end |
| BLAST search | RLC_Facunda_AOCO010682987-1 | 960 | - | 822 - 960 | - | 03 -821 | Partial element, truncated 5´end |
| BLAST search | RLC_Facunda_AOCO010337734-1 | 937 | - | 799 - 937 | - | 01 - 798 | Partial element, truncated 5´end |
| BLAST search | RLC_Facunda_CALP010246332-1 | 914 | - | 776 - 914 | - | 02 - 773 | Partial element, truncated 5´end |
| BLAST search | RLC_Facunda_AOCO010306757-1 | 910 | - | 769 - 910 | - | 01 - 768 | Partial element, truncated 5´end |
| BLAST search | RLC_Facunda_AOCO010292158-1 | 908 | - | 770 - 908 | - | 02 -769 | Partial element, truncated 5´end |
| BLAST search | RLC_Facunda_AOCO010503007-1 | 897 | - | 759 - 897 | - | 03 -758 | Partial element, truncated 5´end |
| BLAST search | RLC_Facunda_AOCO010496849-1 | 892 | - | 754 - 892 | - | 04 - 753 | Partial element, truncated 5´end |
| BLAST search | RLC_Facunda_AOCO010778235-1 | 886 | - | 748 - 886 | - | 49 - 747 | Partial element, truncated 5´end |
| BLAST search | RLC_Facunda_AOCO010674074-1 | 880 | - | 742 - 880 | - | 26 - 738 | Partial element, truncated 5´end |
| BLAST search | RLC_Facunda_AOCO010105933-1 | 869 | - | 731 - 869 | - | 1- 730 | Partial element, truncated 5´end |
| BLAST search | RLC_Facunda_AOCO010438322-1 | 859 | - | 831 - 859 | - | 03 - 791 | Partial element, truncated 5´end |
| BLAST search | RLC_Facunda_AOCO010489717-1 | 850 | - | 712 - 850 | - | 07 - 711 | Partial element, truncated 5´end |
| BLAST search | RLC_Facunda_AOCO010067994-1 | 848 | - | 710 - 848 | - | 11 - 709 | Partial element, truncated 5´end |
| BLAST search | RLC_Facunda_AOCO010059447-1 | 847 | - | 709 - 847 | - | 04 - 708 | Partial element, truncated 5´end |
| BLAST search | RLC_Facunda_AOCO010501503-1 | 843 | - | 705 - 843 | - | 06 - 704 | Partial element, truncated 5´end |
| BLAST search | RLC_Facunda_CALP011056667-1 | 842 | - | 706 - 842 | - | 04 - 619 | Partial element, truncated 5´end |
| BLAST search | RLC_Facunda_AOCO010652134-1 | 842 | - | 705 - 842 | - | 05 - 704 | Partial element, truncated 5´end |
| BLAST search | RLC_Facunda_AOCO010383957-1 | 834 | - | 696 - 834 | - | 03 - 695 | Partial element, truncated 5´end |
| BLAST search | RLC_Facunda_AOCO010333244-1 | 829 | - | 701 - 839 | - | 03 -698 | Partial element, truncated 5´end |
| BLAST search | RLC_Facunda_CALP010704921-1 | 820 | - | 684 - 820 | - | 06 - 683 | Partial element, truncated 5´end |
| BLAST search | RLC_Facunda_AOCO010210244-1 | 811 | - | 671 - 809 | - | 01 - 669 | Partial element, truncated 5´end |
| BLAST search | RLC_Facunda_AOCO010769610-1 | 802 | - | 662 - 802 | - | 02 - 661 | Partial element, truncated 5´end |
| BLAST search | RLC_Facunda_AOCO010041712-1 | 797 | - | 659 - 797 | - | 02 - 658 | Partial element, truncated 5´end |
| BLAST search | RLC_Facunda_AOCO010379179-1 | 784 | - | 646 - 784 | - | 02 - 645 | Partial element, truncated 5´end |
| BLAST search | RLC_Facunda_CALP011680601-1 | 773 | - | 650 - 768 | - | 24 - 649 | Partial element, truncated 5´end |
| BLAST search | RLC_Facunda_AOCO010811368-1 | 750 | - | 599 - 738 | - | 02 - 598 | Partial element, truncated 5´end |
